# New Megastigmane and Polyphenolic Components of Henna Leaves and Their Tumor-Specific Cytotoxicity on Human Oral Squamous Carcinoma Cell Lines

**DOI:** 10.3390/antiox12111951

**Published:** 2023-11-01

**Authors:** Mohamed A. A. Orabi, Esam A. Orabi, Ahmed Abdullah Al Awadh, Mohammed Merae Alshahrani, Basel A. Abdel-Wahab, Hiroshi Sakagami, Tsutomu Hatano

**Affiliations:** 1 Department of Pharmacognosy, College of Pharmacy, Najran University, Najran 66454, Saudi Arabia; 2 Department of Chemistry and Biochemistry, Concordia University, 7141 Sherbrooke Street West, Montréal, QC H4B 1R6, Canada; 3 Department of Clinical Laboratory Sciences, Faculty of Applied Medical Sciences, Najran University, Najran 66454, Saudi Arabia; aaalawadh@nu.edu.sa (A.A.A.A.); mmalshahrani@nu.edu.sa (M.M.A.); 4 Department of Pharmacology, College of Pharmacy, Najran University, Najran 64462, Saudi Arabia; babdelnaem@nu.edu.sa; 5 Meikai University Research Institute of Odontology (M-RIO), 1-1 Keyakidai, Saitama 350-0283, Japan; sakagami@dent.meikai.ac.jp; 6 Graduate School of Medicine, Dentistry and Pharmaceutical Sciences, Okayama University, Tsushima, Okayama 700-8530, Japan; hatano-t@cc.okayama-u.ac.jp

**Keywords:** lythraceae, henna, *Lawsonia inermis*, megastigmane, polyphenols, oral cancer, cytotoxicity, antioxidants, molecular docking

## Abstract

Polyphenols have a variety of phenolic hydroxyl and carbonyl functionalities that enable them to scavenge many oxidants, thereby preserving the human redox balance and preventing a number of oxidative stress-related chronic degenerative diseases. In our ongoing investigation of polyphenol-rich plants in search of novel molecules, we resumed the investigation of *Lawsonia inermis* L. (Lythraceae) or henna, a popular ancient plant with aesthetic and therapeutic benefits. The leaves’ 70% *aq* acetone extract was fractionated on a Diaion HP-20 column with different ratios of H_2_O/an organic solvent. Multistep gel chromatographic fractionation and HPLC purification of the Diaion 75% *aq* MeOH and MeOH fractions led to a new compound (**1**) along with tannin-related metabolites, benzoic acid (**2**), benzyl 6′-*O*-galloyl-*β*-D-glucopyranoside (**3**), and ellagic acid (**4**), which are first isolated from henna. Repeating the procedures on the Diaion 50% *aq* MeOH eluate led to the first-time isolation of two *O*-glucosidic ellagitannins, heterophylliin A (**5**), and gemin D (**6**), in addition to four known *C*-glycosidic ellagitannins, lythracin D (**7**), pedunculagin (**8**), flosin B (**9**), and lagerstroemin (**10**). The compound structures were determined through intensive spectroscopic investigations, including HRESIMS, 1D (^1^H and ^13^C) and 2D (^1^H–^1^H COSY, HSQC, HMBC, and NOESY) NMR, UV, [α]_D_, and CD experiments. The new structure of **1** was determined to be a megastigmane glucoside gallate; its biosynthesis from gallic acid and a *β*-ionone, a degradative product of the common metabolite *β*-carotin, was highlighted. Cytotoxicity investigations of the abundant ellagitannins revealed that lythracin D2 (**7**) and pedunculagin (**8**) are obviously more cytotoxic (tumor specificity = 2.3 and 2.8, respectively) toward oral squamous cell carcinoma cell lines (HSC-2, HSC-4, and Ca9-22) than normal human oral cells (HGF, HPC, and HPLF). In summary, *Lawsonia inermis* is a rich source of anti-oral cancer ellagitannins. Also, the several discovered polyphenolics highlighted here emphasize the numerous biological benefits of henna and encourage further clinical studies to profit from their antioxidant properties against oxidative stress-related disorders.

## 1. Introduction

The study of polyphenolic phytochemicals for drug development has grown significantly in recent decades. Polyphenols can participate in redox activities that are required for various metabolic events due to their many phenolic hydroxyl and carbonyl functionalities. As a result, they can prevent a variety of chronic degenerative illnesses and maintain human homeostasis [1]. The plant polyphenols are widely varied in their chemical structures among different families of the plant kingdom or within the same family, and how they interact—whether specifically or not—with biomolecules relies heavily on both their own physicochemical properties and those of the biomolecule partners [2].

**Figure 1:**
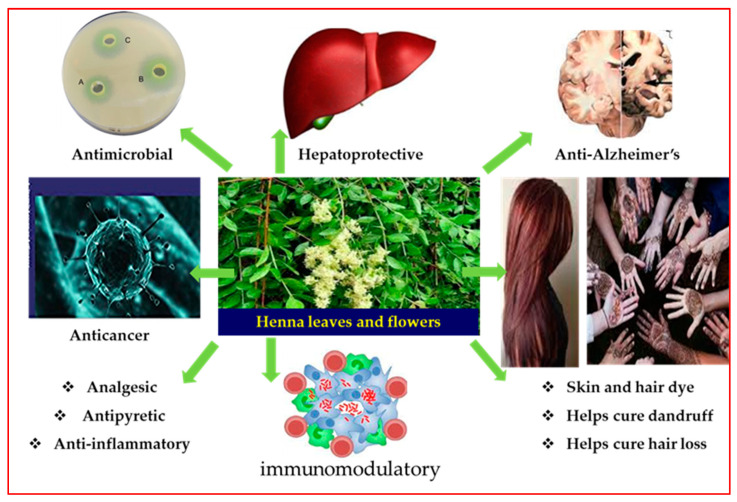
Summary of major bioactivities of henna metabolites.

In our ongoing investigation of polyphenol-rich plants in search of novel molecules, we resumed the investigation of *Lawsonia inermis* L. (Syn. *L. alba*) (Lythraceae), or henna, as one with special healing qualities in ancient medicines. Henna, which refers to the dye prepared from the plant, is naturally grown from Northeast Africa to India and has been extensively used for centuries in the Middle East, Far East, and Northern Africa as a cosmetic dye for nails, hands, hair, and textiles. It has also been used to tackle skin issues, headaches, jaundice, amebiasis, and spleen enlargement [3,4,5]. The plant extracts and purified constituents of henna account for a variety of activities, including anti-Alzheimer’s, antioxidant, hepatoprotective, immunomodulatory, cytotoxic, antibacterial, antifungal, analgesic, anti-inflammatory and antipyretic, hypotensive, sedative, and anticancer effects (Figure 1) [3,6,7,8,9,10]. Oral administration of *L. inermis* leaf extracts significantly suppressed the growth of B16F10 tumors in mice with an increased tumor necrosis area and increased infiltration of mononuclear cells at the site of the tumor [11]. The in vivo antitumor effect of *L. inermis* extracts was directly linked to the enhanced antioxidant activity, which is the main quality of plant polyphenols [11]. In our preceding articles on henna leaf extracts, we demonstrated the occurrence of ellagitannins in abundance and reported on their anticholinesterase and cytotoxic activities [12,13]. The structure and molecular weight of ellagitannins determine their inhibitory capacities against the initiation and spread of tumors, which are subsequently influenced by their antioxidant and binding qualities [12].

The isolation–identification procedures of therapeutic metabolites is a potential research focus for the next steps of our studies. Further phytochemical investigations of different fractions of the *L. inermis* leaf extract may lead to the exploration of novel constituents that could explain the extract’s various biological areas of significance.

In this study, a chromatographic investigation of phenolic-rich fractions of the henna leaf extract led to the isolation of a new megastigmane, lawsoiononoside (**1**), together with the first-time isolation of tannin-related phenolics, benzyl 6′-O-galloyl-*β*-D-glucopyranoside (**2**), benzoic acid (**3**), and ellagic acid (**4**), whereas an extensive investigation of the tannin-rich fractions led to two glucopyranose-type ellagitannins (**5** and **6**) in addition to four known *C*-glycosidic ones (**7**–**10**). The cytotoxicity of the abundant ellagitannins (**8**–**10**) to various human oral cancer and normal cell lines was examined in the present study.

## 2. Materials and Methods

### 2.1. General Experimental Procedures

Electronic circular dichroism (ECD) and ultraviolet (UV) spectra were recorded on JASCO J-720W (JASCO, Tokyo, Japan) and JASCO V-530 (JASCO, Tokyo, Japan) spectrophotometers, respectively. The optical rotation was measured on a JASCO DIP-1000 (JASCO, Tokyo, Japan) digital polarimeter. High-resolution electrospray ionization mass (HRESIMS) spectra were acquired on a Micromass AutoSpec OA-TOF (Manchester, UK) spectrometer. Samples in H_2_O/MeOH (1:1, *v*/*v*) + 0.1% NH_4_OAc were infused into the ESI source at a flow rate of 20 μL/min. The NMR spectra were acquired on a Varian INOVA AS600 (Varian, Palo Alto, CA, USA) instrument (600 MHz for ^1^H and 151 MHz for ^13^C). Chemical shifts are given in *δ* (ppm) relative to that of the solvent signal [(CH_3_)_2_CO-*d*_6_ (*δ*_H_ 2.04; *δ*_C_ 29.8)] on the tetramethylsilane (TMS) scale.

The fractionation and purification procedures were monitored by normal-phase (NP) and reversed-phase (RP) high-performance liquid chromatography (HPLC). The NP-HPLC analyses were performed on a YMC-Pack SIL A-003 (YMC, Kyoto, Japan) column (4.6 × 250 mm) using a mobile phase composed of *n*-hexane/MeOH/tetrahydrofuran/formic acid (55:33:11:1, *v*/*v*) + oxalic acid (450 mg/L). The flow rate was adjusted at 1.5 mL/min at room temperature, and eluates were monitored using a UV detector at 280 nm. The RPHPLC analyses were performed on a YMC-Pack ODS-A A-302 column (4.6 × 150 mm) (YMC, Japan) with a 0.01 M H_3_PO_4_/0.01 M KH_2_PO_4_/MeOH (1:1:0.5, *v*/*v*) mobile phase at a flow rate of 1.0 mL/min at 40 °C and UV detection at 280 nm. Preparative RP-HPLC was performed at 40 °C on a YMC-Pack ODS-A A-324 column (10 × 300 mm) at a flow rate of 2.0 mL/min and the same UV detector. The composition of the mobile phases used in the RPHPLC purifications are specified in the extraction and isolation procedures below. The gels used for column chromatography were Diaion HP-20 (Mitsubishi Chemical, Tokyo, Japan), MCI-gel CHP-20P, Toyopearl HW-40C (TOSOH, Tokyo, Japan), and Sephadex LH-20 (GE Healthcare Bio-Science AB, Sweden).

### 2.2. Plant Material

*L. inermis* leaves were obtained from mature trees around the court collections, Assiut city, Egypt. The plant was authenticated by Professor Salah M. I. El-Najjar, Department of Botany, Assiut University, Egypt. A specimen numbered Li-05013 was kept in the department of Pharmacognosy, Al-Azhar University, Assiut, Egypt.

### 2.3. Extraction and Isolation

Dry *L. inermis* powdered leaves (200 g) were defatted by repeated steeping in *n*-hexane for 1.5 L × 3 consecutive days. The defatted marc was squeezed well and left to dry from the *n*-hexane, and then it was homogenized in 70% *aq* acetone (4 × 1.5 L). The solvent was dried off under vacuum, and the solute was coarsely fractionated on a Diaion HP-20 column (10 × 81 cm, i.d.) with H_2_O (3 L), 50% *aq* MeOH (4.5 L), 75% *aq* MeOH (3 L), MeOH (3 L), and 70% *aq* (CH_3_)_2_CO (2 L), successively. Each eluate was dried at 40 °C under vacuum to afford the dry fraction weights of 37.7 g, 17.24 g, 1.6 g, 0.43 g, and 0.066 g, respectively. The different eluates, except for the 50% *aq* MeOH eluate, revealed plant metabolites with t*_R_* < 4 min in the NP-HPLC chromatograms [14].

The MeOH fraction (0.43 g) from the Diaion column was dissolved in MeOH. The MeOH-soluble part was vacuum-dried (115 mg) and then dissolved in a 15% *aq* MeOH. The soluble part was chromatographed on an ODS column (2.2 × 40 cm, i.d.) with *aq* MeOH (15%, 25%, and 35%) and MeOH. The 25% and 35% *aq* MeOH eluates afforded benzoic acid (**3**, 10 mg) and ellagic acid (**4**, 14 mg), respectively.

The Diaion 75% *aq* MeOH fraction (1.6 g) was dissolved in H_2_O, and the H_2_O-soluble part was applied to an MCI-gel CHP-20P column (1.1 × 37 cm, i.d.) and eluted with H_2_O and H_2_O/MeOH (9:1, 8:2, 7:3, 6:4, 5:5, 0:10, *v*/*v*). The H_2_O/MeOH (6:4, *v*/*v*) eluate (210 mg) was further worked on an ODS column (2.2 × 40 cm, i.d.), and eluted with H_2_O and H_2_O/MeOH (9:1, 8:2, 7.5:2.5, and 5:5, *v*/*v*), collecting 700 drops/fraction. Fractions 58–65 (7.3 mg) from the H_2_O/MeOH (8:2, *v*/*v*) eluate were further purified using preparative RP-HPLC with [H_2_O/CH_3_CN (8:2, *v*/*v*) + 1% CH_3_COOH)] and yielded pure lawsoiononoside (**1**, 1.3 mg) and benzyl 6′-*O*-galloyl-β-D-glucopyranoside (**2**, 1.8 mg).

A part (10 g) of the diaion 50% *aq* MeOH fraction was further fractionated on a Toyopearl HW-40C column (2.2 × 72 cm) eluted with *aq* EtOH (50%, 60%, and 70%), 70% *aq* EtOH/70% *aq* CH_3_)_2_CO gradients (9:1, 8:2, 7:3, 6:4, and 5:5, *v*/*v*), and finally 70% *aq* CH_3_)_2_CO, successively. The Toyopearl factions (1000 drops/fraction) were analyzed via NP-HPLC and/or RPHPLC, and the fractions with similar chromatographic profiles were combined.

Toyopearl fractions T90–T105 (35 mg), eluted with 50% *aq* EtOH, were chromatographed on an MCI-gel CHP-20P column (1.1 × 37 cm) using H_2_O and then H_2_O/MeOH (9:1, 8.5:1.5, 8:2, 7.5:2.5, 7:3, 6.5:3.5, 5:5, and 0:10) as mobile phases. The early eluate with H_2_O/MeOH (8:2, *v*/*v*) afforded crude (40 mg) and pure gemin D (**6**, 3.5 mg). Toyopearl fractions T105–T124 (87 mg), eluted with 50% *aq* EtOH, were purified on an MCI-gel CHP-20P column (1.1 × 37 cm) with the same elution mode. The H_2_O/MeOH (8.5:1.5, *v*/*v*) eluate afforded pedunculagin (**8**, 22.9 mg), while the H_2_O/MeOH (6.5:3.5, *v*/*v*) eluate afforded heterophylliin A (**5**, 2.6 mg). Using the same MCI-gel CHP-20P column and eluants, the Toyopearl fractions T156–T183 (259 mg) afforded flosin B (**9**, 8.1 mg) in the early eluate with H_2_O/MeOH (7.5:2.5, *v*/*v*). The H_2_O-insoluble part of the Toyopearl fractions T240–T347 (347 mg) afforded lagerstroemin (**10**, 46.8 mg). The H_2_O-soluble part was then chromatographed using the same MCI-gel CHP-20P column (1.1 × 37 cm) with the same elution profile. The early eluate with H_2_O/MeOH (8.5:1.5, *v*/*v*) afforded lythracin D (**8**, 13.4 mg).

### 2.4. Spectroscopic Data of Isolated Compounds

- Lawsoiononoside (**1**): Colorless gummy solid, [α]^27^_D_: −125.6 (*c* 1.0, MeOH); UV λ_max_ (MeOH) nm (log ε): 221 (4.2), 240 (4.0), 277 (3.6); ECD (MeOH) [*θ*] (nm): +2.0 × 10^3^ (222), −2.1 × 10^3^ (332), +0.3 × 10^3^ (376); NMR data; HRESIMS *m*/*z* 563.2072 [M + Na]^+^ (calcd for C_26_H_36_O_12_Na, 563.2099) and *m*/*z* 539.2128 [M − H]^−^ (calcd for C_26_H_35_O_12_, 539.2134).
- Benzoic acid (**2**): Colorless crystalline solid, ^1^H NMR (600 MHz, DMSO-*d*_6_) *δ*_H_ 12.9 (br.s, 1H, COOH), 7.95 (dd, *J* = 1.8, 7.2 Hz, 2H, H-2/H-6), 7.62 (tt, *J* = 1.8, 7.2 Hz, 1H, H-4), and 7.50 (dd, *J* = 7.2, 7.2 Hz, 2H, H-3/H-5); ^13^C NMR (151 MHz, DMSO-*d*_6_) *δ*_C_ 167.23 (C-7), 132.89 (C-4), 130.7 (C-1), 129.2 (C-2/C-6), 128.5 (C-3/C-5) [15].
- Benzyl 6′-*O*-galloyl-β-D-glucopyranoside (**4**): White amorphous powder, ^1^H NMR (600 MHz, Me_2_CO-*d*_6_: D_2_O; 9:1): *δ*_H_ 7.33 (2H, dd, *J* = 1.2, 7.8 Hz, H-2/H-6), 7.26 (2H, dt, *J* = 1.2, 7.8 Hz, H-3/H-5), 7.20 (1H, dt, *J* = 1.2, 7.8 Hz, H-4), 7.13 (2H, s, gal H-2″/H-6″), 4.79, 4.59 (each 1H, d, *J* = 12 Hz, H-7), 4.55 [1H, dd, *J* = 1.8, 12 Hz, glc H-6′], 4.39 (1H, d, *J* = 8.4 Hz, glc H-1′), 4.34 (1H, dd, *J* = 6.6, 12.6 Hz, glc H-6′), 3.29 (1H, dd, *J* = 8, 9 Hz, glc H-2′), 3.44 (1H, t, *J* = 9 Hz, glc H-3′), 3.44 (1H, t, *J* = 9 Hz, glc H-4′), 3.57 (1H, ddd, *J* = 1.8, 6, 9 Hz, glc H-5′); ^13^C NMR (151 MHz, Me_2_CO-*d*_6_: D_2_O; 9:1) *δ*_C_: 167.2 (gal C-7″), 146.0 (2C, gal C-3″/C-5″), 138.6 (gal C-4″), 137.7 (C-1), 128.9 (2C, C-3/C-5), 128.8 (2C, C-2/C-6), 128.2 (C-4), 121.0 (gal C-1″), 109.7 (2C, gal C-2″/C-6″), 102.7 (glc C-1″), 77.5 (glc C-3′), 74.8 (glc C-5′), 74.5 (glc C-2′), 71.2 (glc C-4′), 70.9 (C-7′), 64.5 (glc C-6′); ESIMS *m*/*z* 421 [M − H]^−^ [16].
- Ellagic acid (**4**): Pale-yellow amorphous powder, ^1^H NMR (600 MHz, DMSO-*d*_6_) *δ*_H_ 10.69 (br. s, OH), 7.50 (s, 2H, H-2/H-2′); ^13^C NMR (151MHz, DMSO-*d*_6_) *δ*_C_: 159.5 (2C, C-7, C-7′), 148.5 (2C, C-4/C-4′), 140.00 (2C, C-2/C-2′), 136.8 (2C, C-3/C-3′), 112.7 (2C, C-6/C-6′), 110.6 (2C, C-5/C-5′), 108.0 (2C, C-1/C-1′); ESIMS *m*/*z* 301 [M − H]^−^ [17].
- Heterophylliin A (**5**): Off-white amorphous powder; ^1^H NMR (600 MHz, (CH_3_)_2_CO-*d*_6_: D_2_O; 9:1) *δ*_H_ 7.23, 7.03 (each 2H, s, gal H-2/H-6), 6.61, 6.48 (each 1H, s, HHDP H-3, H-3′), 6.39 (1H, d, *J* = 4.2 Hz, glc H-1), 5.64 (1H, t, *J* = 10.2 Hz, glc H-3), 5.22 (1H, dd, *J* = 13.2, 6.6 Hz, glc H-6), 5.05 (1H, t, *J* = 10.2 Hz, glc H-4), 4.55 (1H, br. dd, *J* = 6.6, 10.2 Hz, glc H-5), 4.20 (1H, dd, *J* = 4.2, 10.2 Hz, glc H-2), Hz, 3.75 (1H, br. d, *J* = 13.2 Hz, glc H-6) [18].
- Gemin D (**6**): Off-white amorphous powder, ^1^H NMR (600 MHz, (CH_3_)_2_CO-*d*_6_: D_2_O; 9:1) (α- and β-anomers) *δ*_H_: 7.01, 7.00 (each s, 2H in total, gal H-2/H-6), 6.59, 6.58 (each s, 1H in total, HHDP H-3), 6.44, 6.43 (each s, 1H in total, HHDP H-3ʹ), 5.46, 5.28 (1H in total, each t, *J* = 10.2 Hz, glc H-3α, β), 5.24 (1/2H, d, *J* = 4.2 Hz, glc H-1α), 5.21, 5.18 (1H in total, each dd, *J* = 6.6, 10.2 Hz, H-6α, β), 4.95, 4.92 (1H in total, each t, *J* = 10.2 Hz, H-4α, β), 4.72 (1/2H, d, *J* = 7.2 Hz, H-1β), 4.52, 4.06 (1H in total, each ddd, *J* = 1.2, 6.6, 10.2 Hz, H-5α, β), 3.81 (1/2H, dd, *J* = 4.2, 10.2 Hz, H-2α), 3.78, 3.71 (1H in total, each dd, *J* = 1.2, 13.2 Hz, H-6α, β), 3.57 (1/2H, dd, *J* = 7.2, 10.2 Hz, H-2β) [19].
- Lythracin D (**7**): Off-white amorphous powder, ^1^H NMR (600 MHz, (CH_3_)_2_CO-*d*_6_: D_2_O; 9:1) *δ*_H_: 7.08, 6.57, 6.60 (each 1H, s, valoneoyl-H), 6.73, (1H, s, flavogallonyl-H), 6.91, 6.61, 6.54 (each 1H, s, HHDP-H); 4.90 (1H, d, *J* = 1.8 Hz, glc-1 H-1), 4.81 (1H, t, *J* = 1.8 Hz, glc-1 H-2), 5.08 (1H, t, *J* = 1.8 Hz, glc-1 H-3), 5.62 (1H, dd, *J* = 1.8, 8.4 Hz, glc-1 H-4), 5.38 (1H, ddd, *J* = 1.2, 4.8, 9.6 Hz, glc-1 H-5), 4.92 (1H, dd, *J* = 2.4, 12.6 Hz, glc H-6), 3.73 (1H, d, *J* = 12.6 Hz, glc-1 H-6), 4.75 (1H, d, *J* = 1.8 Hz, glc-2 H-1), 5.01 (1H, t, *J* = 1.8 Hz, glc H-2), 4.50 (1H, dd, *J* = 1.2, 6.6 Hz, glc-2 H-3), 5.12 (1H, t, *J* = 7.2 Hz, glc-2 H-4), 5.55 (1H, dt, *J* = 1.8, 7.2 Hz, glc-2 H-5), 4.93 (1H, dd, *J* = 2.4, 12.6 Hz, glc-2 H-6), 3.89 (1H, d, *J* = 12.6 Hz, glc-2 H-6) [13].
- Pedunculagin (**8**): Off-white amorphous powder, ^1^H NMR [600 MHz, (CH_3_)_2_CO-*d*_6_: D_2_O; 9:1] (α and β-anomer mixture) *δ*_H_: 6.64, 6.63, 6,60, 6.59, 6.54, 6.50, 6.323, 6.321 (each, s, 4H in total, HHDP-H), 5.43 (1/H, t, *J* = 9.6 Hz, glc H-3α), 5.41 (1/2H, d, *J* = 3.6 Hz, glc H-1α), 5.24, 5.20 (1H in total, each dd, *J* = 6.6, 12.6 Hz, glc H-6 α, β), 5.19 (1/2H, t, *J* = 9.6 Hz, glc H-3β), 5.033, 5.027 (1H in total, each t, *J* = 9.6Hz, glc H-4 α, β), 5.01 (1/2H, d, *J* = 7.8 Hz, glc H-1β), 4.82 (1/2H, dd, *J* = 7.8, 9.6 Hz, glc H-2β), 4.57, 4.18 (1H in total, each ddd, *J* = 1.8, 6.6, 9.6 Hz, glc H-5α, β), 3.83, 3.76 (each 1H, dd, *J* = 1.2, 12.6 Hz, glc H-6α, β] [13].
- Flosin B (**9**): Off-white amorphous powder, ^1^H NMR [600 MHz, (CH_3_)_2_CO-*d*_6_: D_2_O; 9:1] *δ*_H_: 7.57, 7.16, 7.12 (each 1H, s, valoneoyl dilactone-H), 6.84, 6.45, 6.35 (each 1H, s, HHDP-H), 5.51 (1H, dd, *J* = 2.4, 8.4 Hz, glc H-4), 5.18 (1H, br.dd, *J* = 4.8, 9.6 Hz, glc H-5), 4.98 (1H, t, *J* = 2.4 Hz, glc H-3), 4.97 (1H, d, *J* = 2.4 Hz, glc H-1), 4.78 (1H, t, *J* = 1.8 Hz, glc H-2), 4.70 (1H, dd, J = 3.6, 13.2 Hz, glc H-6), 3.71 (1H, d, J = 12.6 Hz, glc H-6) [13].
- Lagerstroemin (**10**): Off-white amorphous powder, ^1^H NMR [600 MHz, (CH_3_)_2_CO-*d*_6_: D_2_O; 9:1] *δ*_H_: 7.57, 7.16, 7.13 (each 1H, s, valoneoyl dilactone-H), 6.76, 6.53, 6.36 (each 1H, s, HHDP-H), 5.57 (1H, d, *J* = 4.8 Hz, glc H-1), 5.38 (1H, t, *J* = 1.8 Hz, glc H-3), 5.36 (1H, dd, *J* = 2.4, 9 Hz, glc H-5), 5.19 (1H, dd, *J* = 3.6, 9 Hz, glc H-4), 4.64 (1H, dd, *J* = 3, 12.6, glc H-6), 4.61 (1H, dd, *J* = 1.8, 4.8, glc H-2), 3.72 (1H, d, *J* = 12.6, glc H-6) [13].

### 2.5. Cytotoxicity

#### 2.5.1. Cell Culture

Four oral squamous cell carcinoma (OSCC) cell lines (Ca9-22 derived from gingiva; HSC-2, HSC-3, and HSC-4 derived from tongue) were purchased from RIKEN Cell Bank, Tsukuba, Japan. Three human oral mesenchymal cell types, [gingival fibroblast (HGF), periodontal ligament fibroblast (HPLF), and pulp cell (HPC)], at 10–18 population doubling level were cultured at 37 °C in DMEM supplemented with 10% heat-inactivated FBS (56 °C, 30 min), 100 U/mL penicillin G, and 100 µg/mL streptomycin sulfate under a humidified 5% CO_2_ atmosphere [20].

It is best to use human epithelial normal oral cells, if possible, for the comparison of drug-sensitivity with OSCC. However, when two human normal epithelial cells (human oral keratinocyte (HOK) and human gingival epithelium progenitors (HGEP)) were cultured in the regular culture medium (DMEM +10% heat-inactivated FBS), their growths were immediately stopped. Therefore, in order to maintain their growth, it was necessary to culture them in the commercially available special media supplemented with growth factors. However, we found that such stimulated epithelial (HOK/HGEP) cells began to grow like cancer cell lines, showing extremely high sensitivity against many anticancer drugs (camptothecin, SN-38 (active principal of irinotecan), doxorubicin, daunorubicin, etoposide, mitomycin C, 5-FU, docetaxel, melphalan and even molecular-targeted drug, gefitinib) [21]. At present, normal human epithelial cells cannot be used as controls. Based on this background, we used three normal oral mesenchymal cells (HGF, HPLF, HPC) in the present study. Further studies are necessary to establish the optimization of the culture condition of normal epithelial cells for use as controls and the composition of HPC cells.

#### 2.5.2. Cytotoxicity Assay

Cells were detached from the culture dishes using 0.25% trypsin, inoculated at 2×10^3^ cells per 100 μL in a 96-microwell plate and incubated for 48 h to ensure complete cell attachment to the well. The culture medium was replaced with 100 μL of fresh medium containing different concentrations of the test compounds (**7** (0.9, 1.7, 3, 6.7, 13, 27, 54, 107 μM); **8** (2, 4, 8, 16, 32, 64, 128, 255 μM); **9** (1.3, 2.5, 5.10, 20, 40, 81, 162 μM); **10** (1.3, 2.5, 5.10, 20, 40, 81, 162 μM); DXR (0.08, 0.16, 0.31, 0.63, 1.25, 2.5, 5, 10 μM); 5-FU (8, 16, 31, 63, 125, 250, 500, 1000 μM); and DMSO vehicle (0.008, 0.016, 0.031, 0.063, 0.125.0.25, 0.5, 1%)). Cells were further incubated for 48 h, and the number of viable cells relative to the control was determined via the MTT assay, which is based on MTT metabolism to formazan by viable cells, as described previously [20]. In brief, cells were incubated in the microplate for 2 h with 0.2 mg/mL MTT, the purple formazan precipitate was solubilized by adding 100 μL DMSO, OD560 was recorded on a microplate reader (Infinite F50R; TECAN, Männedorf, Switzerland), and the readings were adjusted for cytotoxicity caused by the vehicle. The concentration of test compound that produced cytotoxicity by 50% (CC_50_) was determined in triplicate from the dose–response curves.

CC_50_ values against three normal human oral mesenchymal cell lines (HGF, HPLF, and HPC) and four OSCC cell lines (Ca9-22, HSC-2, HSC-3, and HSC-4) were determined. The mean CC_50_ values for the normal and tumor cell lines were calculated and the ratio of the means gives the tumor specificity (TS):

TS = mean CC_50_ normal cell lines/mean CC_50_ OSCC cell lines

#### 2.5.3. Statistical Analysis

All analyses were carried out in triplicate to ensure robustness and reliability. The data are presented as mean ± standard deviation (SD). Graph Pad Prism 7 and Microsoft Excel 2010 were used for the statistical and graphical evaluations.

## 3. Results and Discussions

### 3.1. Structure Determination of the Isolated Compounds

**Figure 2:**
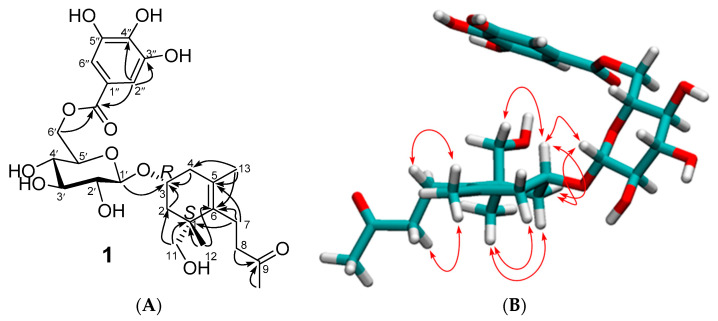
Structure of new megastigmane (**1**). (**A**) Key HMBC correlations. (**B**) Important NOESY correlations.

A 70% aq acetone extract of *L. inermis* leaves was fractionated using a Diaion HP-20 gel (2.3. Extraction and Isolation). The multistep isolation using gel chromatography combined with preparative HPLC purification afforded a new megastigmane glucoside gallate (**1**, Figure 2), along with the known benzyl 6′-O-galloyl-β-D-glucopyranoside (**2**) from the Diaion 75% aq MeOH fraction, and benzoic acid (**3**) and ellagic acid (**4**) from the Diaion MeOH fraction. In addition, two O-glycosidic ellagitannins, heterophylliin A (**5**) and gemin D (**6**), and four C-glycosidic ellagitannins, lythracin D (**7**), pedunculagin (**8**), flosin B (**9**), and lagerstroemin (**10**) were purified from the Diaion 50% aq MeOH eluate.

#### 3.1.1. Structure of the New Megastigmane (**1**)

Compound **1** was isolated as a colorless gummy solid, and its molecular formula, C_26_H_36_O_12_, was established from the HRESIMS molecular ion peak at *m*/*z* 563.2072 [M + Na]^+^ (calculated for C_26_H_36_O_12_Na, 563.2099) and *m*/*z* 539.2128 [M − H]^−^ (calculated for C_26_H_35_O_12_, 539.2134), as well as the ^13^C NMR data (Table 1). The ^1^H NMR spectrum of **1** (Figures S3–S5) displayed spectroscopic features typical of a galloyl moiety [22]; an aromatic 2H singlet (*δ*_H_ 7.13, H-2″/H-6″) that exhibits HSQC correlation with a non-oxygenated aromatic carbon (*δ*_C_ 109.6, 2C, C-2″/C-6″) and HMBC correlations with oxygenated aromatic carbon (*δ*_C_ 146, 2C, C-3″/C-5″, and *δ*_C_ 137.7, C-4″), a quaternary aromatic carbon (*δ*_C_ 120.5, C-1″), and a carbonyl carbon (*δ*_C_ 167.2, C-7″). The ^1^H NMR and ^1^H–^1^H COSY spectra of **1** (Table 1, Figures S6–S8) exhibited a resonating system of seven aliphatic proton spins at *δ*_H_ 4.49–3.19 (Table 1), with a large coupling pattern of the glucose H-2′–H-5′ (*J*_H-1′–H-2′_ = 7.8, *J*_H-2′–H-3′_ = *J*_H-3′–H-4_ = *J*_H-4′–H-5′_ = 9.6 Hz), highlighting the ^4^*C*_1_ conformation of the glucopyranose moiety [23]. A large coupling constant of the glucose H-1′ proton signal (*δ*_H_ 4.49, *J* = 7.8 Hz) indicates the *β*-configuration of the glucose’s anomeric center [22]. The HSQC correlations of the glucose proton signals (Figure S10) enabled the assignments of the ^13^C chemical shifts (*δ*_C_ 102.2, 74.5, 77.5, 71.3, 74.8, 64.8) to the glucose carbons C-1′–C-6′, respectively (Table 1). The ^13^C NMR spectrum of **1** (Figure S9) also exhibited a system of thirteen carbons (Table 1), characteristic of the megastigmane moiety [24]. Aided by the ^1^H–^1^H COSY and HSQC spectroscopic data, the system is separated into two components. The first is composed of nine carbons, recognized by their 1D NMR and the HSQC data: one oxygenated methine carbon *δ*_C_ 72.3 (C-3) (correlated in the HSQC spectrum to a 1H multiplet signal at *δ*_H_ 4.14 (H-3)), two non-oxygenated methylene carbons at *δ*_C_ 41.6 (C-2) (correlated in the HSQC spectrum to the H_2_-2 proton signals at *δ*_H_ 1.21 (1H, t, *J* = 8.4 Hz, H-2ax) and 2.25 (1H, ddd, *J* = 2.4, 5.4, 14.4 Hz, H-2eq) and at *δ*_C_ 39.2 (C-4) (correlated in the HSQC spectrum to the H_2_-4 proton signals at *δ*_H_ 1.94 (1H, br.dd, *J* = 9.6, 16.2 Hz, H-4ax) and *δ*_H_ 2.30 (1H, dd, *J* = 5.4, 16.2 Hz, H-4eq)), one oxygenated methylene carbon at *δ*_C_ 68.6 (C-12) (correlated in the HSQC spectrum to *δ*_H_ 3.39 and 3.28 (each 1H, d, *J* = 11 Hz)), two tetrasubstituted olefinic carbons at *δ*_C_ 128.8 (C-5) and 136.6 (C-6), and a quaternary carbon at *δ*_C_ 43.5 (C-1). The remaining two methyl carbons of this component appear at *δ*_C_ 24.3 and 19.8, and one of them is correlated in the HSQC with a 3H-singlet at an up-field shift of 0.91 (H3-11), and the other is correlated with the proton signal at a relatively low-field shift *δ*_H_ 1.54 (3H, brs, H_3_-11). The ^1^H chemical shift of the latter corresponds to the methyl group on an olefenic carbon, and the broadening of the signal is explained by the W-shaped ^1^H–^1^H coupling with the H-4 proton signal (*δ*_H_ 1.94, 1H, br.dd) [25]. The second component of the megastigmane moiety was found to be composed of a chain of four carbons: the 1D together with the 2D (^1^H–^1^H COSY, Figures S6–S8) and HSQC (Figures S10–S12) spectra substantiated the presence of two methylene groups (*δ*_H_ 2.14 and 2.2 (2H, dd, H_2_-7), correlated with a carbon signal at *δ*_C_ 22.3 (C-7) and *δ*_H_ 2.48 (2H, t, H_2_-8), correlated with a carbon signal at *δ*_C_ 44.2 (C-8)). The other two carbons were identified as methyl and ketone carbons: the methyl proton signal at *δ*_H_ 2.08 (3H, s, H_3_-10) exhibits an HSQC correlation with the carbon peak at *δ*_C_ 29.8 (C-10) and an HMBC correlation with a ketonic carbonyl carbon peak at *δ*_C_ 209.3. The same carbonyl carbon is also correlated with the H_2_-8 signal at *δ*_H_ 2.48 in the HMBC spectrum. This four-carbon segment, proposed as –CH_2_–CH_2_–CO–CH_3_, was placed at the olefenic carbon C-6 (*δ*_C_ 133.6), as deduced from the HMBC correlations of the H-7 signal at *δ*_H_ 2.14 with C-6 (*δ*_C_ 133.6) and the neighboring carbons C-1 (*δ*_C_ 43.5) and C-5 (*δ*_C_ 128.8). The connectivity of the structural components (galloyl, glucosyl, and megastigmane moieties) of **1** was then substantiated based on the HMBC correlations (Figures S13 and S14).

The galloyl unit was positioned on the glucose C-6′; this was based on the low-field shift of the glucose C-6′ signal (*δ*_C_ 64.8) in addition to a weak HMBC correlation of glucose-H-6 (*δ*_H_ 4.16) with the galloyl carbonyl carbon peak (*δ*_C_ 167.2). The down-field shift of the glucose anomeric carbon (*δ*_C_ 102.2, C-1′) indicated the presence of an *O*-glucosidic linkage between the megastigmane aglycone and glucose C-1′, which was confirmed by the HMBC correlation of the glucose H-1′ (*δ*_H_ 4.49) and the megastigmane moiety C-3 (*δ*_C_ 72.8).

The ^1^H NMR spectrum showed the H-3 signal at *δ*_H_ 4.14 as a multiplet in the ^1^H NMR spectrum, where the coupling constants of *J*_2ax-3_, *J*_2eq-3_, *J*_4ax-3_, and *J*_4eq-3_ with the adjacent proton signals were assigned to be 12.6 Hz, 8.4 Hz, 9.6 Hz, and 5.4 Hz, respectively. These NMR data indicated the equatorial orientation of the substituted hydroxyl group at C-3. This was further evidenced by the ^1^H-^1^H nuclear Overhauser effect spectroscopy (NOESY) correlations between the axially oriented glucose H-1′ proton (*δ*_H_ 4.49) and both H-3 (*δ*_H_ 4.14) and H-4eq (*δ*_H_ 2.30) (Figures S15–S17) corresponding to the *R* chirality of C-3 in compound **1** [26]. In the skeleton of megastigmanes, it has been documented, thus far, that the hydroxyl group at the C-3 position frequently has an equatorial orientation [27]. The NOESY spectrum exhibited a key correlation of H-3 with H_2_-11 and NOESY correlations of both H-2ax (*δ*_H_ 1.21, t) and H-4ax (*δ*_H_ 1.94) with H_3_-12 (*δ*_H_ 0.91, 3H, s, methyl), indicating the *S* chirality of C-1. Among the four possible stereoisomers (1*S*3*S*, 1*R*3*R*, 1*S*3*R*, and 1*R*3*S,* Figure S41) of **1**, these NOESY correlations were consistent with the 1*S*3*R* isomer. The H_3_-12 methyl signal, as well as the H_3_-13 methyl signal (*δ*_H_ 1.54, 3H, s), exhibited NOESY correlations with H_2_-7 (*δ*_H_ 2.14 and 2.20) and H_2_-8 (*δ*_H_ 2.48). The NOESY correlations between each pair of protons of H_2_-2 and H_2_-4 and H_2_-7 were also detected (Figures S15–S17). Importantly, the practically recoded ECD spectrum of **1** was compared with the computationally calculated ECD (see procedures in the Supplementary Materials) of the four possible stereoisomers. Despite the noisy Cotton effects of the practically measured ECD, the overall spectrum was consistent with that calculated for the 1*S*3*R* isomer (Figure S42). According to these findings, the new structure of **1** was concluded to be a gallate derivative of a megastigmane glucoside, as shown in Figure 2, and given the name lawsoiononoside (**1**).

**Figure 3:**
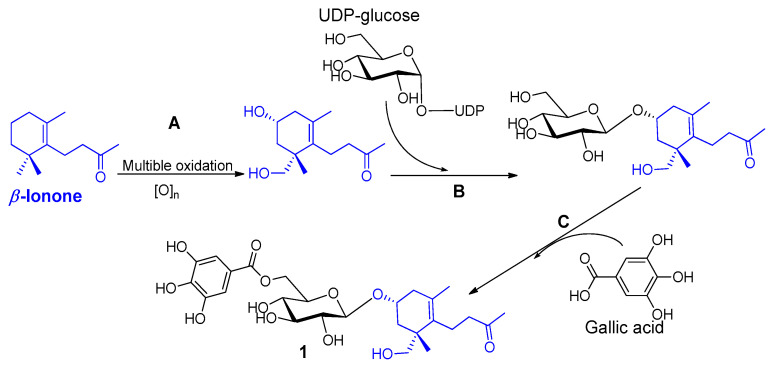
Suggested biosynthesis of compound **1** from β-ionone and gallic acid.

Except for the megastigmane aglycone *β*-ionone, this is the first report on the isolation of a megastigmane from henna. Megastigmanes are oxygenated isonorterpenoids with a C13 carbon skeleton and frequently referred to as oxidative by-products from *β*-carotenoids [28]. The biosynthesis of compound **1** from *β*-ionone and gallic acid is assumed to be as shown in Figure 3. This biotransformation involves the oxidation of C-3 and C-11 of the megastigmane basic skeleton (Step A), followed by the glucosylation (Step B) and esterification of the glucose OH-6 with gallic acid (Step C). Direct glycosylation of the megastigmane with a 6-*O*-galloyl glucose is also possible.

Numerous megastigmane glucosides have been shown to have antibacterial, anti-inflammatory, anticancer, and hepatoprotective effects, which are strongly correlated with their antioxidant capacity [29,30,31]. The antioxidant activity of megastigmane glycosides has been demonstrated through their capacity to scavenge DPPH free radicals and suppress the process of lipid peroxidation [31]. The bioactivities of this class of metabolites, coupled with their antioxidant power, suggests the need for further studies in the future on these intriguing bioactive small molecules as a scaffold of drug discovery.

#### 3.1.2. Structure of Known Compounds (**2**–**10**)

##### Structure of Compounds **2**–**4**

**Figure 4:**
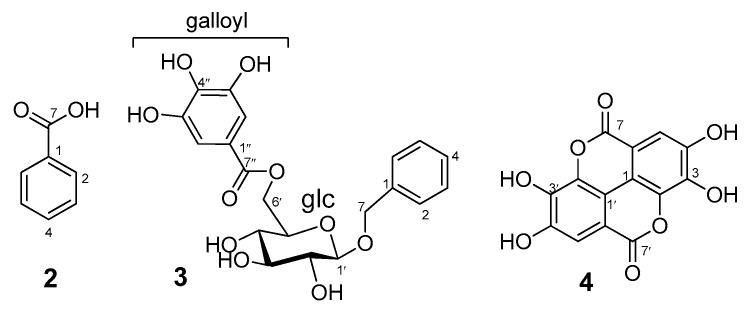
Structures of the compounds **2**–**4**, first isolated from henna.

- Benzoic acid (**3**), together with the tannin-related metabolite benzyl 6′-*O*-galloyl-β-D-glucopyranoside (**2**), and ellagic acid (**4**) are isolated for first time from henna, and their structures (Figure 4) were determined from the NMR and ESIMS data referenced to the literature values as follows:

- Compound **2** was isolated as **a** colorless crystalline solid. Its structure was identified from the ^1^H NMR pattern of a monosubstituted benzene [*δ*_H_ 7.95 (2H, dd, *J* = 1.8, 7.2 Hz, H-2/H-6), 7.62 (1H, tt, *J* = 1.8, 7.2 Hz, H-4), and 7.50 (2H, dd, *J* = 7.2, 7.2 Hz, H-3/H-5)] (Figure S18) and the ^13^C NMR data *δ*_C_ 132.89 (C-4), 130.7 (C-1), 129.2 (C-2/C-6), and 128.5 (C-3/C-5) (Figure S20), which are consistent with a monosubstituted benzene as well. The presence of a carboxyl group was recognized by a broad proton signal in the low-field region (*δ*_H_ 12.9) and a carbonyl carbon peak at *δ*_C_ 167.23 (C-7). The ESIMS molecular ion peak at *m*/*z* 121 [M–1]^–^ confirmed the identification of **2** as a benzoic acid [15].
- Compound **3** was isolated as a white amorphous powder. Its 1H NMR spectrum (Figure S22) exhibited proton signals of a ^1^*C*_4_ *β*-D-glucopyranose core {*δ*_H_ 4.55 [1H, dd, *J* = 1.8, 12 Hz, glc H-6], 4.39 (1H, d, *J* = 8.4 Hz, glc H-1), 4.34 (1H, dd, *J* = 6.6, 12.6 Hz, glc H-6), 3.29 (1H, dd, *J* = 8, 9 Hz, glc H-2), 3.44 (1H, t, *J* = 9 Hz, glc H-3), 3.44 (1H, t, *J* = 9 Hz, glc H-4), and 3.57 (1H, ddd, *J* = 1.8, 6, 9 Hz, glc H-5)} [12,13,22]. The spectrum also exhibited a 2H singlet at *δ*_H_ 7.13 (2H, s, galloyl H-2/H-6), which exhibited a HSQC correlation with the 2C peak at *δ*_C_ 109.7 (2C, galloyl C-2/C-6). These are characteristic of a galloyl moiety [12,13,22]. The spectrum also exhibited a proton signal of a benzyl moiety at *δ*_H_ 7.33 (2H, dd, *J* = 1.2, 7.8 Hz, H-2/H-6), 7.26 (2H, dt, *J* = 1.2, 7.8 Hz, H-3/H-5), 7.20 (1H, dt, *J* = 1.2, 7.8 Hz, H-4), and 4.79, 4.59 (each 1H, d, *J* = 12 Hz, H-7). The ^13^C NMR spectrum (Figure S24) exhibited carbon peaks corresponding to the structural components of **2** (*viz*, benzyl, galloyl, and glucose). The HMBC correlations between the galloyl 2H singlet (*δ*_H_ 7.13) and the glucose H_2_-6 (*δ*_H_ 4.55 and 4.34) with the galloyl carbonyl carbon (*δ*_C_ 167.2) evidenced the placement of the galloyl moiety at C-6 of the glucose core. The HMBC correlations of the benzyl H_2_-7 at *δ*_H_ 4.79 and 4.59 (each 1H, d, *J* = 12 Hz) with the glucose anomeric carbon (*δ*_C_ 102.7), as well as the HMBC correlation of the glucose anomeric proton (*δ*_H_ 4.39) with the benzylic carbon C-7 (*δ*_C_ 70.9), indicated the placement of the benzyl moiety at the anomeric center of the glucose, as shown by the structural formula of **2** (Figure 4). The ESIMS negative ion peak at *m*/*z* 421 [M − H]^−^ confirmed the identification of **2** as benzyl 6′-*O*-galloyl-*β*-D-glucopyranoside [16]. Compound **4** was isolated as a pale yellow amorphous powder. Its ^1^H NMR spectrum (Figure S27) exhibited a 2H singlet signal at *δ*_H_ 7.50 (H-2/H-2′) and a broad singlet at *δ*_H_ 10.69 (br. s, OH). The ^13^C NMR spectrum exhibited seven carbon peaks, each equivalent to two carbons [*δ*_C_ 159.5 (2C, C-7/C-7′), 148.5 (2C, C-4/C-4′), 140.0 (2C, C-2/C-2′), 136.8 (2C, C-3/C-3′), 112.7 (2C, C-6/C-6′), 110.6 (2C, C-5/C-5′), and 108.0 (2C, C-1/C-1′). These are characteristics for an ellagic acid. Based on the molecular ion peak at *m*/*z* 301 [M − H]^−^ in the ESIMS spectrum, we confirmed the structure of **4** to be ellagic acid [17].

It is worth noting that Ye et al., 2007, have reported two resonances (*δ*_H_ 7.14 and 7.47) for the equivalent ellagic acid protons (H-5 and H-5′, respectively), which are incorrect and misleading NMR data [32].

##### Structure of the *O*-Glycosidic Ellagitannins **5** and **6**

**Figure 5:**
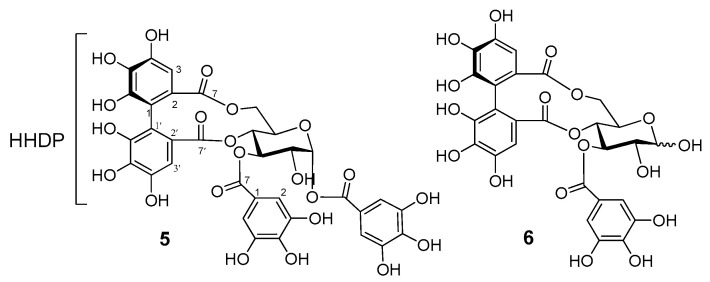
Structure of the ellagitannins **5** and **6**.

Heterophylliin A (**5**) and gemin D (**6**) were isolated first from the plant, and hence, their structures were determined from the following spectroscopic data and comparison with the literature values:

- Compound **5** was isolated as an off-white amorphous powder. Its ^1^H NMR spectrum (Figure S29) exhibited aromatic proton signals at *δ*_H_ 7.23, 7.03 (each 2H, s, galloyl H-2/H-6) of two galloyl units and two 1H singlets (*δ*_H_ 6.61 and 6.48), indicative of the presence of a hexahydroxydiphenoyl (HHDP) unit [22]. A spin system of seven aliphatic proton sets, as evident from the ^1^H–^1^H COSY correlations (Figure S30), were assigned for the ^4^*C*_1_ D-glucopyranose core as follows: *δ*_H_ 6.39 (1H, d, *J* = 4.2 Hz, glc H-1), 5.64 (1H, t, *J* = 10.2 Hz, glc H-3), 5.22 (1H, dd, *J* = 13.2, 6.6 Hz, glc H-6), 5.05 (1H, t, *J* = 10.2 Hz, glc H-4), 4.55 (1H, br. dd, *J* = 6.6, 10.2 Hz, glc H-5), 4.20 (1H, dd, *J* = 4.2, 10.2 Hz, glc H-2), and Hz, 3.75 (1H, br. d, *J* = 13.2 Hz, glc H-6). The up-field shift of the glucose H-2 signals (*δ*_H_ 4.20) indicated the deacylation of the glucose 2-OH, whereas the small coupling constant (*J* = 4.2 Hz) of the anomeric proton signal indicated the alpha-oriented C-O bond at the glucose’s anomeric center [14,18]. The large difference in the chemical shifts of the glucose H_2_-6 signals (*δ*_H_ 4.20 and 3.75) indicated the placement of the HHDP group at O-4/O-6 of the glucose, and therefore, the remaining O-1 and O-3 should be acylated by the galloyl units [14]. These data, together with the comparison with the literature values [18], led to the identification of **5** as heterophylliin A (**5**, Figure 5).

- Compound **6** was isolated as an off-white amorphous powder. Its ^1^H NMR spectrum exhibited double resonances of all proton signals (Section 2.4. Spectroscopic Data of Isolated Compounds), which indicated the existence of **6** as a mixture of α- and β-anomer. The aromatic proton signals [*δ*_H_ 7.01, 7.00 (each s, 2H in total), *δ*_H_ 6.59, 6.58 (each s, 1H in total), and *δ*_H_ 6.44, 6.43 (each s, 1H in total) are consistent with the galloyl H-2/H-6, HHDP H-3, and HHDP H-3ʹ, respectively [22]. Proton signals of a glucose core [*δ*_H_: 5.46, 5.28 (1H in total, each t, *J* = 10.2 Hz, glc H-3α, β), 5.24 (1/2H, d, *J* = 4.2 Hz, glc H-1α), 5.21, 5.18 (1H in total, each dd, *J* = 6.6, 10.2 Hz,), 4.95, 4.92 (1H in total, each t, *J* = 10.2 Hz, H-4α, β), 4.72 (1/2H, d, *J* = 7.2 Hz, H-1β), 4.52, 4.06 (1H in total, each ddd, *J* = 1.2, 6.6, 10.2 Hz, H-5α, β), 3.81 (1/2H, dd, *J* = 4.2, 10.2 Hz, H-2α), 3.78, 3.71 (1H in total, each dd, *J* = 1.2, 13.2 Hz, H-6α, β), and 3.57 (1/2H, dd, *J* = 7.2, 10.2 Hz, H-2β) were detected in the NMR spectrum of **6**. These double resonances indicate the presence of the unacylated OH group of the anomeric center [14]. Resonances of the glucose H-2 signals with the up-field shifts (*δ*_H_ 3.81 and 3.57) also evidenced the existence of the free OH-2 on the glucose core. Similar to heterophylliin A (**5**), the wide difference in the chemical shifts of the glucose H_2_-6 signals (*δ*_H_ 5.21, 5.18, H-6α, β, and *δ*_H_ 3.78, 3.71, H-6α, β) indicated the bridging of the HHDP moiety at O-4/O-6 of the glucose core, leaving the glucose O-3 for the galloyl moiety. These spectroscopic data, which are reasonably identical with those previously published, confirmed the identity of **6** as gemin D (Figure 5) [19].

##### Structure of *C*-Glycosidic Ellagitannins (**7**–**10**)

**Figure 6:**
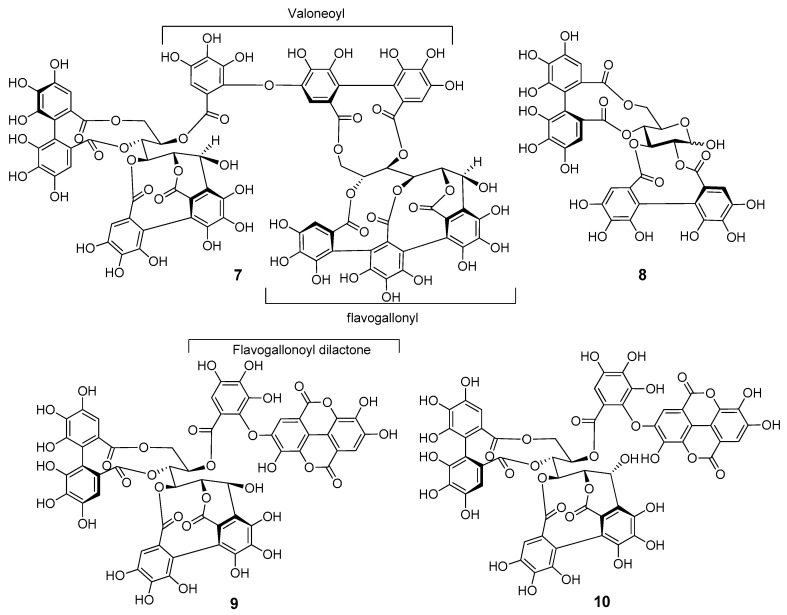
Structures of the known ellagitannins **7**–**10**.

The *C*-glycosidic ellagitannins **7**–**10** (Figure 6) are known compounds which were previously isolated from the same plant resource. Their structures were identified from NMR data (Section 2.4. Spectroscopic Data of Isolated Compounds and Figures S33–S40) and compared with the published NMR data as lythracin D (**7**), pedunculagin (**8**), flosin B (**9**), and lagerstroemin (**10**) [13].

### 3.2. Cytotoxicity of Ellagitannins against Oral Cancer Cell Lines

The anticancer properties of *L. inermis* leaf, root, flower, and bark extracts have been thoroughly studied using animal models and cancer cell lines [33]. Water extracts of *L. inermis* leaves inhibited the growth of various cancer cell lines to varying degrees [34,35].

In the current study, four *C*-type glycosidic ellagitannins, which are typical of lythraceae plants, were among the isolated groups of phytochemicals that were detected in high concentrations in the aqueous acetone extract of henna leaves. Lately, it has become more common to investigate the applicability of ellagitannins to treat illnesses brought on by oxidative stress, such as cancer and neurodegenerative diseases [36]. Ellagitannins from various plants have demonstrated prominent multi-mechanistic antitumor properties [37]. The selective cytotoxicity against human oral squamous cell carcinoma (OSCC) vs. normal oral cells was examined for four ellagitannins: lythracin D (**7**), flosin B (**9**), lagerstroemin (**10**), of unknown cytotoxicity, and pedunculagin (**8**), a known antitumor agent [38,39,40]. All four ellagitannins, as well as doxorubicin (DXR), were cytotoxic toward the OSCC cell lines, whereas 5-FU was cytostatic. Pedunculagin and lythracin D demonstrated significant tumor-specific cytotoxicity (TS = 2.8 and 2.3, respectively), while flosin B and lagerstroemin exhibited low specificity (TS = 1.7 and 1.5, respectively) (Table 2). The primary mechanism by which ellagitannins and their derivatives, including ellagic acid, exhibit anticancer activities is through their antioxidant capacity, which is dependent on both iron chelation activity and direct radical scavenging and varies with the degree of hydroxylation [41]. According to the literature, ellagitannins’ antitumor properties are primarily influenced by their antioxidant activity and their ability to reduce inflammation; research-based evidence suggested that they have the ability to regulate secretory growth factors and proinflammatory molecules like IL-6, TGF-β, TNF-α, IL-1β, and IFN-γ [42]. Research has demonstrated that a number of oligomeric ellagitannins have in vivo anticancer effects against mouse models of sarcoma 180 and MM2, which was linked to a strengthened host immune response. In vitro research using tumor cell lines has shown that a number of ellagitannins, as well as their constituent acids gallic and ellagic, showed a strong cytotoxicity against carcinoma cell lines and a low cytotoxicity toward normal cells [20]. Our findings in the present study are consistent with the previous reports on ellagitannin’s cytotoxic activity [20,42] and highlight the potential of the development of *L. inermis* ellagitannins as anti-oral cancer drugs. Furthermore, the occurrence of *C*-type glycosidic ellagitannins in abundance is significant from a chemotaxonomic perspective as well as for explaining the multiple biological benefits of henna, such as its anti-inflammatory, antioxidant, and anticancer properties.

### 3.3. Previously Reported Bioactivities of the Known Compounds **2**–**10**

*L. inermis* is a medicinal plant that is popular in Unani medicine and ancient Egyptian medical papyri, with promising biological attributes: strong fungicidal, antibacterial, virucidal, antiparasitic, antiamoebiasis, astringent, and antihemorrhagic [4,5]. In addition, henna’s plant extracts were found to be effective sedative, hypotensive, anti-Alzheimer’s, antioxidant, hepatoprotective, immunomodulatory, and anticancer agents [3,6,7,8,10,43]. In this study, we report on the isolation and structural identification of a megastigmane (**1**) as well as several ellagitannins and tannin-related phenolics. Reviewing biological studies conducted on these compounds (Table 3) emphasized the above-mentioned biological qualities of henna.

## 4. Conclusions

To further our search for the exploration of new phytomolecules from polyphenolic-rich plants, we isolated ten compounds with various characteristics from *L. inermis* leaves. The novel megastigmane structure lawsoiononoside (**1**) is also reported here based on intensive spectroscopic data. Given the interesting bioactivities of megastigmanes [29,30,31], our discovery of **1** will spur an additional exploration of the unique megastigmane structures from *L. inermis*. The cytotoxicity of the ellagitannins lythracin D (**7**) and pedunculagin (**8**) against the OSCC cell lines indicates the potential development of anti-oral cancer therapeutics based on *L. inermis*. According to recent studies, ellagitannins’ antioxidant, and anti-inflammatory properties, which include the ability to modulate proinflammatory mediators including IL-6, TGF-β, TNF-α, IL-1, and IFN-γ, are mostly involved in their anticancer effects [42]. The tannins **7**–**10**, albeit having a relatively moderate TS, may be an appropriate radiosensitizer to diminish tumor resistance in cancer radiotherapy, as recently shown to be the case for pentagalloyl glucose and a gallotannin-rich extract from *Bouea macrophylla* seed [55]. The different identified chemicals discussed here also draw attention to the numerous biological benefits of henna and encourage additional clinical research in order to benefit from the antibacterial, anti-inflammatory, antioxidant, hepatoprotective, and anticancer properties of this promising ancient plant.

## Figures and Tables

**Table 1:** The 1D (^1^H, ^13^C NMR) and 2D (HMBC and NOESY) spectroscopic data of compound **1**.

Position	*δ*_H_	*δ*_C_	HMBC	NOESY
1		43.5		
2ax	1.21, 1H, t (12.6)	41.6	C-2, C-3, C-4, C-11, C-12	H-11, H-2ex
2eq	2.25, 1H, br.dd (8.4, 14.4)		C-1	H-2aq
3	4.14, 1H, m	72.8		H-1′, H-4eq, H-12
4ax	1.94, 1H, br.dd (9.6, 16.8)	39.2	C-3, C-5, C-6	H-11, H-4eq
4eq	2.30, 1H, br. dd (5.4, 16.8)		C-2, C-5, C-6	H-4ax
5		128.8		
6		133.6		
7	2.14, 1H, dd (8.4, 14.4) ^a^, 2.20, 1H, dd (8.4, 14.4) ^a^	22.3	C-5, C-6, C-8, C-9	H-11, H-13
8	2.48, 2H, t (8.4)	44.2	C-7, C-9	H-11, H-13
9		209.3		
10	2.08, 3H, s	29.8 ^b^	C-8, C-9	H-7
11 11	3.39, 1H, d (11), 3.28, 1H, d (11)	68.6	C-1, C-2, C-11	H-3
12	0.91, 3H, s	24.3	C-1, C-2, C-6, C-12	H-2ax, H-4ax, H-7, H-8
13	1.54, 3H, br.s	19.8	C-4, C-5, C-6	H-7, H-8
1′	4.49, 1H, d (7.8)	102.2	C-3	H-3, H-4eq
2′	3.19, 1H, dd (7.8, 9.6)	74.5	C-1′, C-3′	
3′	3.44,1H, t (9.6)	77.5	C-2′, C-4′	
4′	3.35,1H, t (9.6)	71.3	C-3′, C-5′, C-6′	
5′	3.61, 1H, ddd (1.8, 9.6,	74.8	C-3′, C-4′	
6′	4.61,1H, dd (1.8, 13.8)	64.8	C-5′, C-7″	
	4.16, 1H, dd (7.2, 12.0)			
1″		120.5		
2″/6″	7.13, 2H, s	109.6		
3″/5″		146		
4″		137.7		
7″		167.2		

^a^ Approximate multiplicity because proton signal overlapped with solvent impurities; ^b^ Hidden under the solvent peaks and identified from HSQC correlation with *δ*_H_ 2.08 (H_3_-10), which together with H_2_-8 (*δ*_H_ 2.48, 2H, t) exhibited HMBC correlations with C-9 (*δ*_C_ 209.3).

**Table 2:** Cytotoxicity of ellagitannins **7**–**10** against human OSCC cell lines and normal oral cells ^a^.

	CC_50_ (μM) ^b^				CC_50_ (μM) ^b^				
	Ca9-22	HSC-2	HSC-4	Mean	HGF	HPLF	HPC	Mean	TS ^c^
Lythracin D (7)	89 ± 4	67 ± 8	48 ± 10	68	157 ± 36	152 ± 5	152 ± 4	154	2.3
Pedunculagin (8)	93 ± 5	48 ± 4	48 ± 3	63	178 ± 11	187 ± 4	157 ± 7	174	2.8
Flosin B (9)	180 ± 18	66 ± 8	80 ± 4	109	200	174 ± 22	169 ± 14	181	1.7
Lagerstroemin (10)	185 ± 14	75 ± 11	87 ± 2	115	200	159 ± 9	155 ± 5	171	1.5
Doxorubicin	0.5 ± 0.05	0.16 ± 0.04	0.17 ± 0.01	0.29	10 ± 0	10 ± 0	10 ± 0	10	>35
5-FU	33 ± 12	10 ± 2	35 ± 6	26	1000 ± 0	1000 ± 0	1000 ± 0	1000	>38.0

^a^ Ca9-22, HSC-2, and HSC-4 are oral squamous cell carcinoma (OSCC) cell lines. HGF, HPLF, and HPC are the normal oral cells. ^b^ Results are expressed as the mean ± SD of three independent experiments. ^c^ TS = [CC_50_ (HGF) + CC_50_ (HPLF) + CC_50_ (HPC)]/[CC_50_ (Ca9-22) + CC_50_ (HSC-2) + CC_50_ (HSC-4)].

**Table 3:** Reported pharmacological activities of isolated compounds **2**–**10**.

Compound	Reported Biological Activity
benzyl-6′-*O*-galloyl-β-D-glucopyranoside (2)	Antifungal activity against *C. albicans* clinical isolates and reference strains [44]. Moderate inhibitory effects against lipopolysaccharide-induced nitric oxide production in RAW 264.7 cells [45].
Benzoic acid (3)	Antibacterial and antifungal activity [46].
Ellagic acid (4)	Antioxidant, anti-inflammatory, antimutagenic, antiproliferative, antiallergic, antiatherosclerotic, cardioprotective, hepatoprotective, nephroprotective, and neuroprotective properties [47].
Heterophylliin A (5)	Antidiabetic (moderate inhibitory effect against dipeptidyl peptidase IV and α-glucosidase) activity [48]. Antioxidant, antiallergic, and anti-inflammatory [49].
Gemin D (6)	Cytotoxic and chemo-preventive, in vitro anti-HIV activity, anti-Leishmania donovani amastigote, a potent growth-inhibitor of sarcoma 180 cells in mice. A potent inhibitory effect as a 3-hydroxy-3-methylglutaryl-coenzyme-A reductase [50]. In vivo antigenotoxic activity [51].
Lythracin D (7)	Anticholinesterase [13], cytotoxic to OSCC cell lines [12].
Pedunculagin (8)	Anti-acne vulgaris (mediated by anti-inflammatory activity and 5α-reductase inhibition) [52], antibacterial, and antihemolytic [53]. Antitumor activity [38,39,40].
Flosin B (9)	Contributes to the antidiabetic activity of *Lagerstroemia speciosa* by increasing glucose uptake of rat adipocytes [54]. Anti-acetylcholinesterase [13].
Lagerstroemin (10)	Increased glucose uptake of rat adipocytes and could be responsible for lowering of blood glucose level, as shown by *Lagerstroemia speciosa* extract [54].

## Data Availability

C:\Users\ASUS\Downloads\NMRSpectroscopic data of the reported compounds are included in the Supplementary Material File and available from the corresponding author upon request.

## Supplementary Materials

The following supporting information can be downloaded at: https://www.mdpi.com/article/10.3390/antiox12111951/s1, Figure S1: Positive-mode HRESIMS spectrum of compound **1**; Figure S2: Negative-mode HRESIMS spectrum of compound **1**; Figure S3: ^1^H NMR spectrum of compound **1** [600 MHz, acetone-*d*_6_ + D_2_O (9+1)]; Figure S4: Expanded ^1^H NMR spectrum of compound **1** [600 MHz, acetone-*d*_6_ + D_2_O (9+1)]; Figure S5: Expanded ^1^H NMR spectrum of compound **1** [600 MHz, acetone-*d*_6_ + D_2_O (9+1)]; Figure S6: ^1^H-^1^H COSY spectrum of compound **1** [600 MHz, acetone-*d*_6_ + D_2_O (9+1)]; Figure S7: Expanded ^1^H-^1^H COSY spectrum of compound **1** [600 MHz, acetone-*d*_6_ + D_2_O (9+1)]; Figure S8: Expanded ^1^H-^1^H COSY spectrum of compound **1** [600 MHz, acetone-*d*_6_ + D_2_O (9+1)]; Figure S9: ^13^C NMR spectrum of compound **1** [151 MHz, acetone-*d*_6_ + D_2_O (9+1)]; Figure S10: HSQC spectrum of compound **1** [600 MHz, acetone-d6 + D2O (9+1)]; Figure S11: Expanded HSQC spectrum of compound **1** [600 MHz, acetone-*d*_6_ + D_2_O (9+1)]; Figure S12: Expanded HSQC spectrum of compound **1** [600 MHz, acetone-*d*_6_ + D_2_O (9+1)]; Figure S13: HMBC spectrum of compound **1** [600 MHz, acetone-*d*_6_ + D_2_O (9+1)]; Figure S14: Expanded HMBC spectrum of compound **1** [600 MHz, acetone-*d*_6_ + D_2_O (9+1)]; Figure S15: NOESY spectrum of compound **1** [600 MHz, acetone-*d*_6_ + D_2_O (9+1)]; Figure S16: Expanded NOESY spectrum of compound **1** [600 MHz, acetone-*d*_6_ + D_2_O (9+1)]; Figure S17: Expanded NOESY spectrum of compound **1** [600 MHz, acetone-*d*_6_ + D_2_O (9+1)]; Figure S18: ^1^H NMR spectrum of compound **2** (600 MHz, DMSO-*d*_6_); Figure S19: ^1^H-^1^H COSY spectrum of compound **2** (600 MHz, DMSO-*d*_6_); Figure S20: ^13^C NMR spectrum of compound **2** (151 MHz, DMSO-*d*_6_); Figure S21: HSQC spectrum of compound **2** (600 MHz, DMSO-*d*_6_); Figure S22: ^1^H NMR spectrum of compound **3** [600 MHz, acetone-*d*_6_ + D_2_O (9+1)]; Figure S23: ^1^H-^1^H COSY spectrum of compound **3** [600 MHz, acetone-*d*_6_ + D_2_O (9+1)]; Figure S24: ^13^C spectrum of compound **3** [151 MHz, acetone-*d*_6_ + D_2_O (9+1)]; Figure S25: HSQC spectrum of compound **3** [600 MHz, acetone-*d*_6_ + D_2_O (9+1)]; Figure S26: HMBC spectrum of compound **3** [600 MHz, acetone-*d*_6_ + D_2_O (9+1)]; Figure S27: ^1^H NMR spectrum of compound **4** (600 MHz, DMSO-*d*_6_); Figure S28: ^13^C NMR spectrum of compound **4** (151 MHz, DMSO-*d*_6_); Figure S29: ^1^H NMR spectrum of compound **5** [600 MHz, acetone-*d*_6_ + D_2_O (9+1)]; Figure S30: ^1^H-^1^H COSY spectrum of compound **5** [600 MHz, acetone-*d*_6_ + D_2_O (9+1)]; Figure S31: ^1^H NMR spectrum of compound **6** [600 MHz, acetone-*d*_6_ + D_2_O (9+1)]; Figure S32: ^1^H-^1^H COSY spectrum of compound **6** [600 MHz, acetone-*d*_6_ + D_2_O (9+1)]; Figure S33: ^1^H NMR spectrum of compound **7** [600 MHz, acetone-*d*_6_ + D_2_O (9+1)]; Figure S34: ^1^H-^1^H COSY spectrum of compound **7** [600 MHz, acetone-*d*_6_ + D_2_O (9+1)]; Figure S35: ^1^H NMR spectrum of compound **8** [600 MHz, acetone-*d*_6_ + D_2_O (9+1)]; Figure S36: ^1^H-^1^H COSY spectrum of compound **8** [600 MHz, acetone-*d*_6_ + D_2_O (9+1)]; Figure S37: ^1^H NMR spectrum of compound **9** [600 MHz, acetone-*d*_6_ + D_2_O (9+1)]; Figure S38: ^1^H-^1^H COSY spectrum of compound **9** [600 MHz, acetone-*d*_6_ + D_2_O (9+1)]; Figure S39: ^1^H NMR spectrum of compound **10** [600 MHz, acetone-*d*_6_ + D_2_O (9+1)]; Figure S40: ^1^H-^1^H COSY spectrum of compound **10** [600 MHz, acetone-*d*_6_ + D_2_O (9+1)]; Experimental procedures of TD-DFT calculation of the ECD spectrum of **1**; Figure S41: Three-dimensional structures of possible stereoisomers of compound **1**; Figure S42: Calculated (**A**) and practically measured (**B**) ECD spectra of compound **1** [56,57,58,59].
