# Supplementary material for: New Megastigmane and Polyphenolic Components of Henna Leaves and Their Tumor-Specific Cytotoxicity on Human Oral Squamous Carcinoma Cell Lines
Source: Antioxidants (Basel). 2023 Nov 1;12(11):1951. doi: 10.3390/antiox12111951 (PMC10669829; doi:10.3390/antiox12111951)
Supplement: Supplementary file 1 [file antioxidants-12-01951-s001.zip › antioxidants-2655999-SI.pdf]

antioxidants

Supplementary materials

New Megastigmane and Polyphenolic Components of Henna Leaves and Their Tumor-Specific Cytotoxicity on Human Oral Squamous Carcinoma Cell Lines

Mohamed A. A. Orabi[1,*], Esam A Orabi[2,*], Ahmed Abdullah Al Awadh[3], Mohammed Merae Alshahrani[3], Basel A. Abdel-Wahab[4], Hiroshi Sakagami[5] and Tsutomu Hatano[6]

1 Department of Pharmacognosy, College of Pharmacy, Najran University, Najran 66454, Saudi Arabia; mohamedorabi@azhar.edu.eg; maorabi@nu.edu.sa.
2 Department of Chemistry and Biochemistry, Concordia University, 7141 Sherbrooke Street West, Montréal, Québec H4B 1R6, Canada; e_orabi@live.concordia.ca.
3 Department of Clinical Laboratory Sciences, Faculty of Applied Medical Sciences, Najran University, Najran 66454, Saudi Arabia; aaalawadh@nu.edu.sa (AAAA), mmalshahrani@nu.edu.sa (MMA)
4 Department of Pharmacology, College of Pharmacy, Najran University, Najran 64462, Saudi Arabia; babdelnaem@nu.edu.sa
5 Meikai University Research Institute of Odontology (M-RIO), 1-1 Keyakidai, Sakado, Saitama 350-0283, Japan; sakagami@dent.meikai.ac.jp
6 Graduate School of Medicine, Dentistry and Pharmaceutical Sciences, Okayama University, Tsushima, Okayama 700-8530, Japan; hatano-t@cc.okayama-u.ac.jp
* Correspondence: mohamedorabi@azhar.edu.eg (M.A.A.O.; e_orabi@live.concordia.ca (E.A.O.)

Table of content

List of content	Page
Figure S1: Positive mode HRESIMS spectrum of compound **1**	S4
Figure S2: Negative mode HRESIMS spectrum of compound **1**	S5
Figure S3: ^{1}H NMR spectrum of compound **1** [600 MHz, acetone-d_6 + D_2O (9+1)]	S6
Figure S4: Expanded ^{1}H NMR spectrum of compound **1** [600 MHz, acetone-d_6 + D_2O (9+1)]	S7
Figure S5: Expanded ^{1}H NMR spectrum of compound **1** [600 MHz, acetone-d_6 + D_2O (9+1)]	S8
Figure S6: ^{1}H-^{1}H COSY spectrum of compound **1** [600 MHz, acetone-d_6 + D_2O (9+1)]	S9
Figure S7: Expanded ^{1}H-^{1}H COSY spectrum of compound **1** [600 MHz, acetone-d_6 + D_2O (9+1)]	S10
Figure S8: Expanded ^{1}H-^{1}H COSY spectrum of compound **1** [600 MHz, acetone-d_6 + D_2O (9+1)]	S11
Figure S9: ^{13}C NMR spectrum of compound **1** [151 MHz, acetone-d_6 + D_2O (9+1)]	S12
Figure S10: HSQC spectrum of compound **1** [600 MHz, acetone-d6 + D2O (9+1)]	S13
Figure S11: Expanded HSQC spectrum of compound **1** [600 MHz, acetone-d_6 + D_2O (9+1)]	S14
Figure S12: Expanded HSQC spectrum of compound **1** [600 MHz, acetone-d_6 + D_2O (9+1)]	S15
Figure S13: HMBC spectrum of compound **1** [600 MHz, acetone-d_6 + D_2O (9+1)]	S16
Figure S14: Expanded HMBC spectrum of compound 1 [600 MHz, acetone-d_6 + D_2O (9+1)]	S17
Figure S15: NOESY spectrum of compound **1** [600 MHz, acetone-d_6 + D_2O (9+1)]	S18
Figure S16: Expanded NOESY spectrum of compound **1** [600 MHz, acetone-d_6 + D_2O (9+1)]	S19
Figure S17: Expanded NOESY spectrum of compound **1** [600 MHz, acetone-d_6 + D_2O (9+1)]	S20
Figure S18: ^{1}H NMR spectrum of compound **2** (600 MHz, DMSO-d_6)	S21
Figure S19: ^{1}H-^{1}H COSY spectrum of compound **2** (600 MHz, DMSO-d_6)	S22
Figure S20: ^{13}C NMR spectrum of compound **2** (151 MHz, DMSO-d_6)	S23
Figure S21: HSQC spectrum of compound **2** (600 MHz, DMSO-d_6)	S24
Figure S22: ^{1}H NMR spectrum of compound **3** [600 MHz, acetone-d_6 + D_2O (9+1)]	S25
Figure S23: ^{1}H-^{1}H COSY spectrum of compound **3** [600 MHz, acetone-d_6 + D_2O (9+1)]	S26
Figure S24: ^{13}C spectrum of compound **3** [151 MHz, acetone-d_6 + D_2O (9+1)]	S27
Figure S25: HSQC spectrum of compound **3** [600 MHz, acetone-d_6 + D_2O (9+1)]	S28

Figure S26: HMBC spectrum of compound **3** [600 MHz, acetone-d_6 + D_2O (9+1)] S29

Figure S27: ^{1}H NMR spectrum of compound **4** (600 MHz, DMSO-d_6) S30

Figure S28: ^{13}C NMR spectrum of compound **4** (151 MHz, DMSO-d_6) S31

Figure S29: ^{1}H NMR spectrum of compound **5** [600 MHz, acetone-d_6 + D_2O (9+1)] S32

Figure S30. ^{1}H-^{1}H COSY spectrum of compound 5 [600 MHz, acetone-d_6 + D_2O (9+1)] S33

Figure S31: ^{1}H NMR spectrum of compound **6** [600 MHz, acetone-d_6 + D_2O (9+1)] S34

Figure S32: ^{1}H-^{1}H COSY spectrum of compound 6 [600 MHz, acetone-d_6 + D_2O (9+1)] S35

Figure S33: ^{1}H NMR spectrum of compound **7** [600 MHz, acetone-d_6 + D_2O (9+1)] S36

Figure S34: ^{1}H-^{1}H COSY spectrum of compound 7 [600 MHz, acetone-d_6 + D_2O (9+1)] S37

Figure S35: ^{1}H NMR spectrum of compound **8** [600 MHz, acetone-d_6 + D_2O (9+1)] S38

Figure S36: ^{1}H-^{1}H COSY spectrum of compound **8** [600 MHz, acetone-d_6 + D_2O (9+1)] S39

Figure S37: ^{1}H NMR spectrum of compound **9** [600 MHz, acetone-d_6 + D_2O (9+1)] S40

Figure S38: ^{1}H-^{1}H COSY spectrum of compound **9** [600 MHz, acetone-d_6 + D_2O (9+1)] S41

Figure S39: ^{1}H NMR spectrum of compound **10** [600 MHz, acetone-d_6 + D_2O (9+1)] S42

Figure S40: ^{1}H-^{1}H COSY spectrum of compound **10** [600 MHz, acetone-d_6 + D_2O (9+1)] S43

Experimental procedures of TD-DFT calculation of the ECD spectrum of 1 S44

Figure S41: 3D structures of possible stereoisomers of compound **1** S45

Figure S42: Calculated (A) and practically measured (B) ECD spectra of compound **1** S46

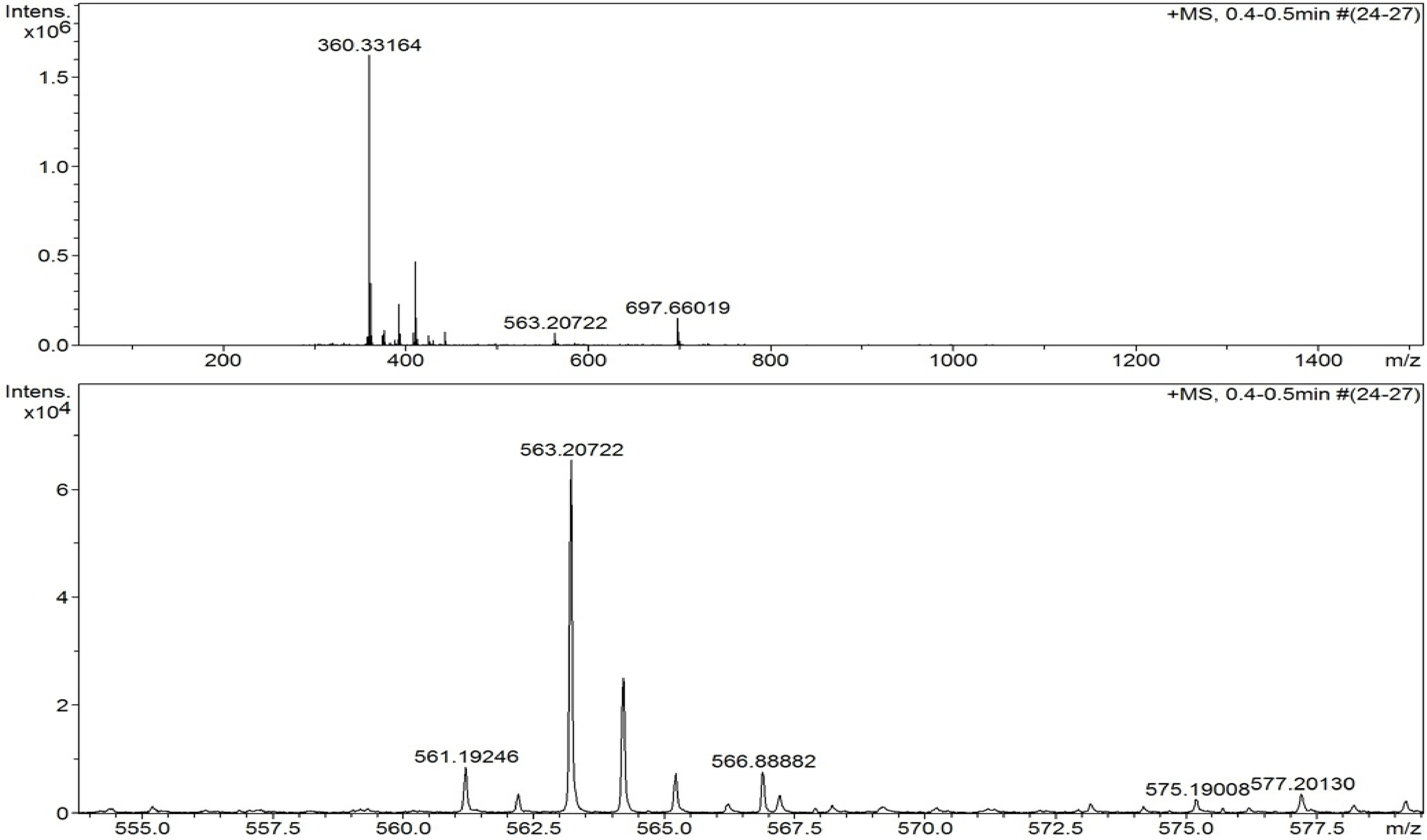

Figure S1. Positive mode HRESIMS spectrum of compound **1**

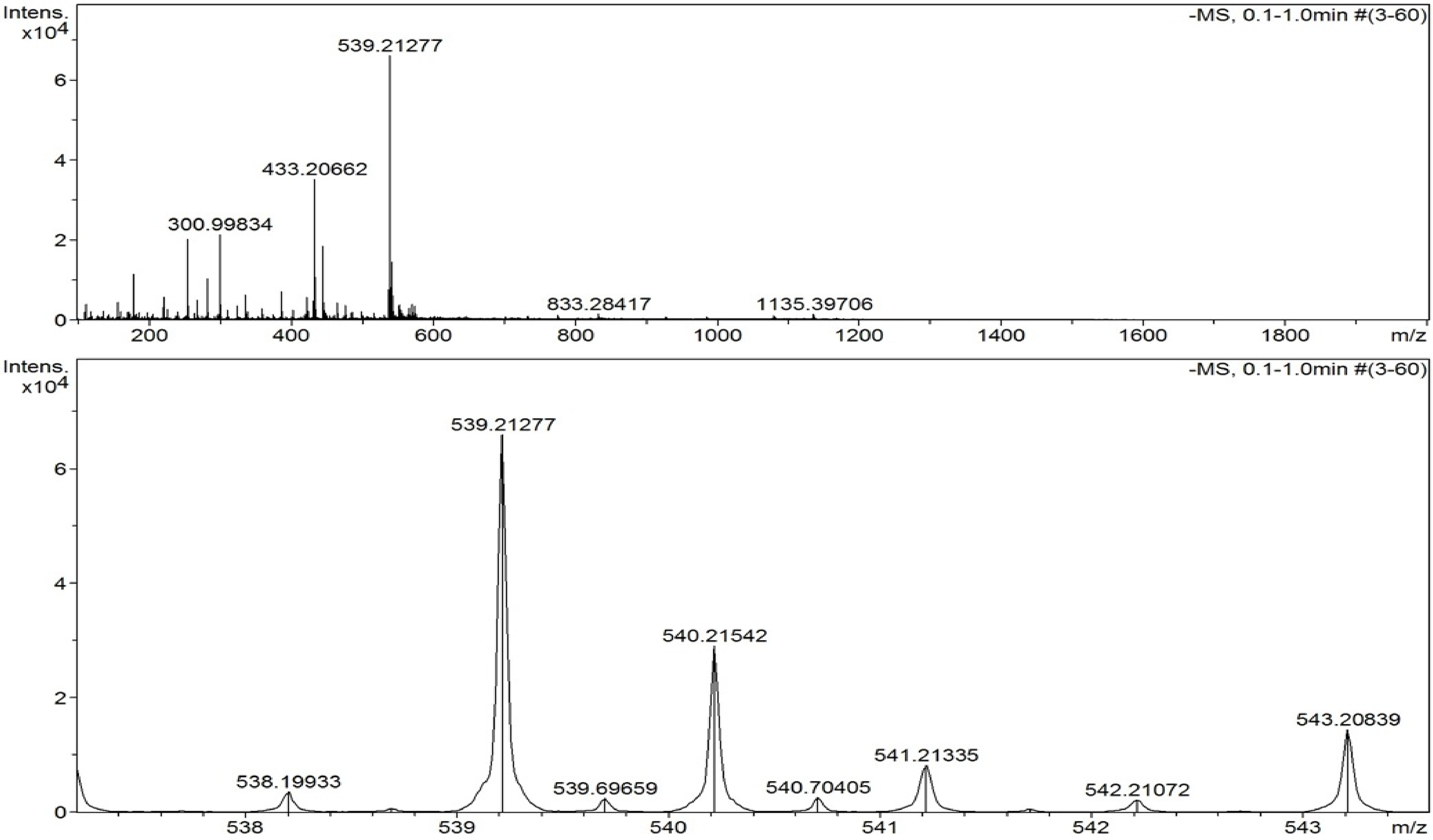

Figure S2. Negative mode HRESIMS spectrum of compound **1**

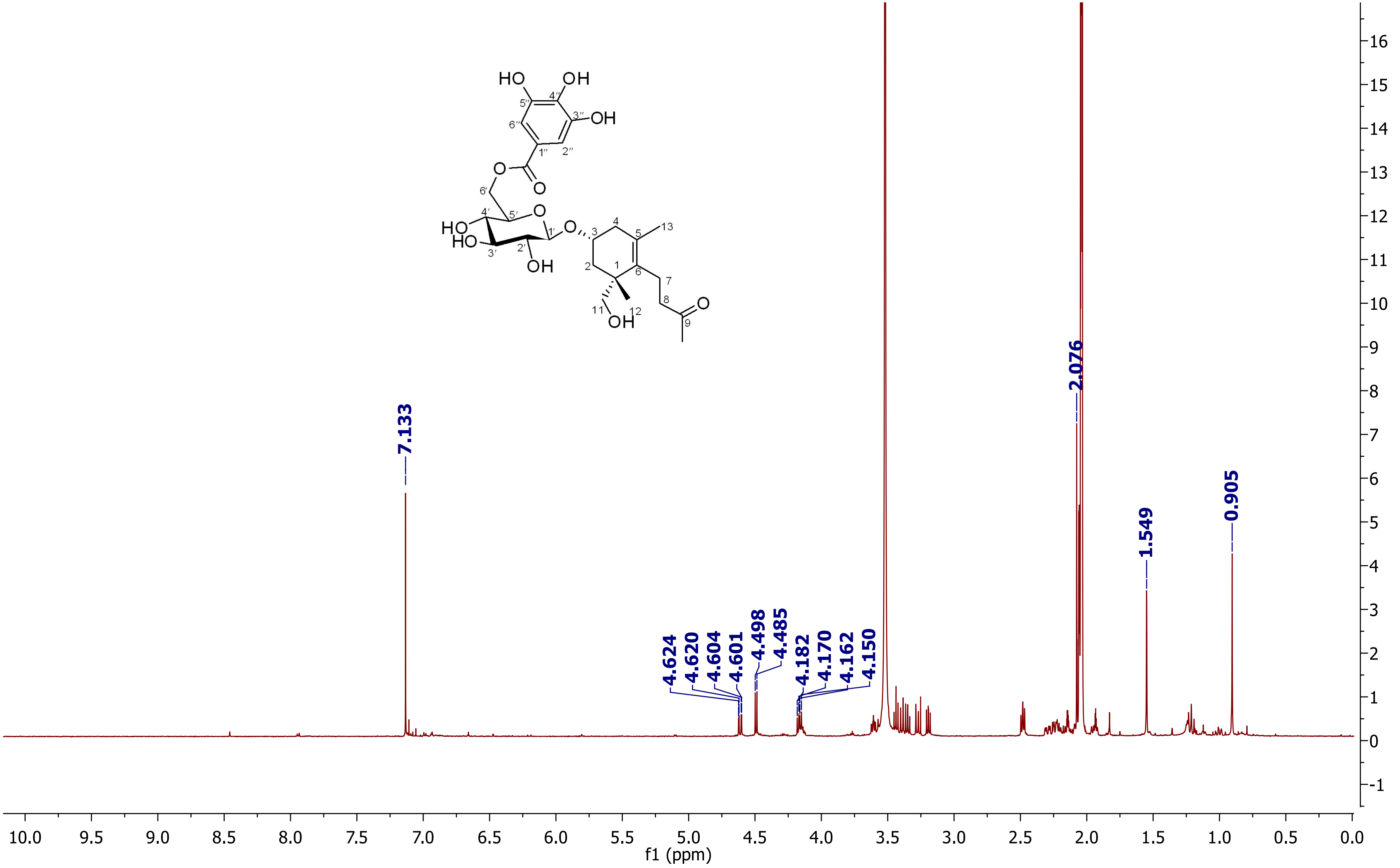

Figure S3. ^{1}H NMR spectrum of compound **1** [600 MHz, acetone-d_6 + D_2O (9+1)]

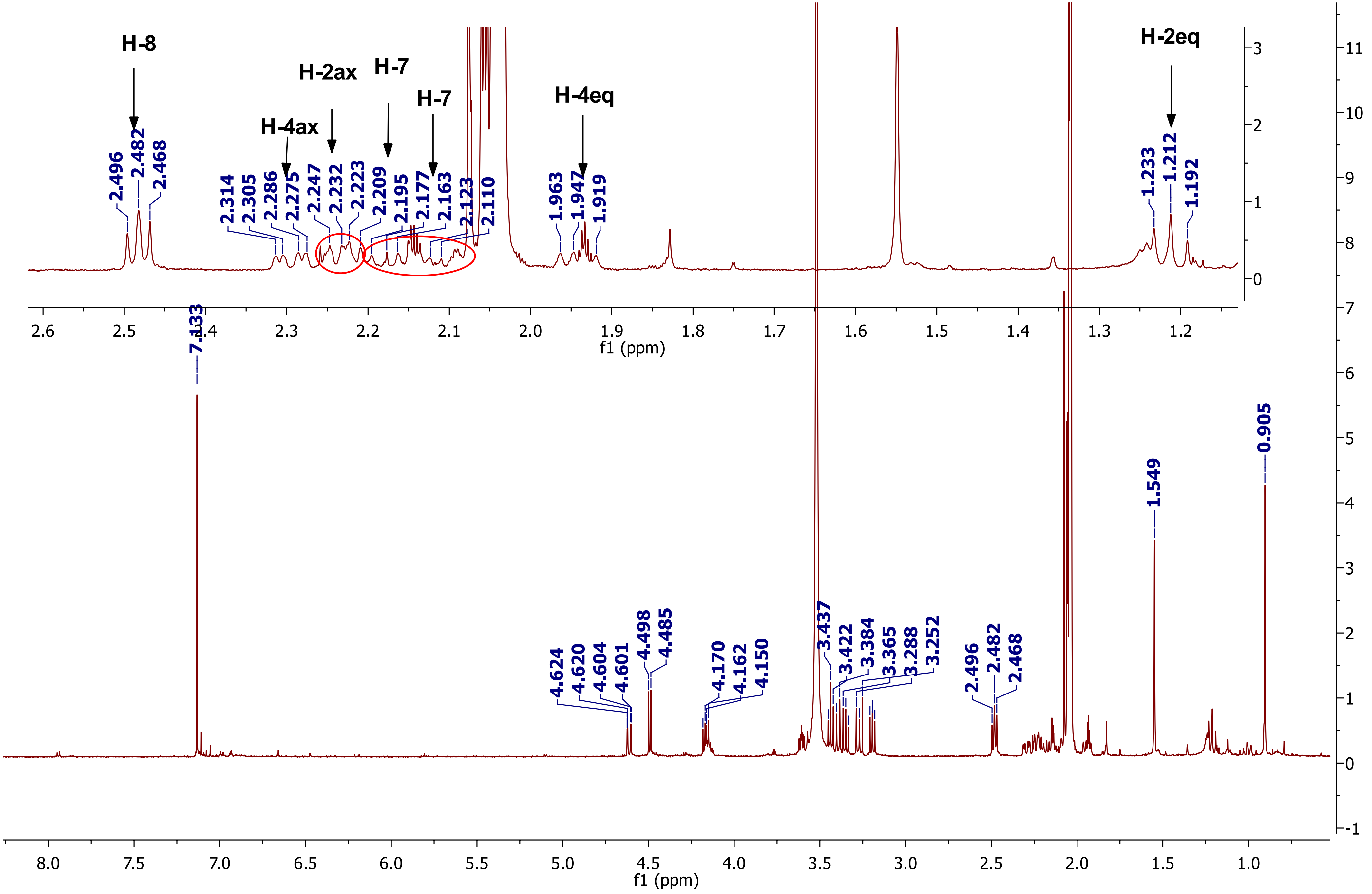

Figure S4. Expanded ^{1}H NMR spectrum of compound **1** [600 MHz, acetone-d_6 + D_2O (9+1)]

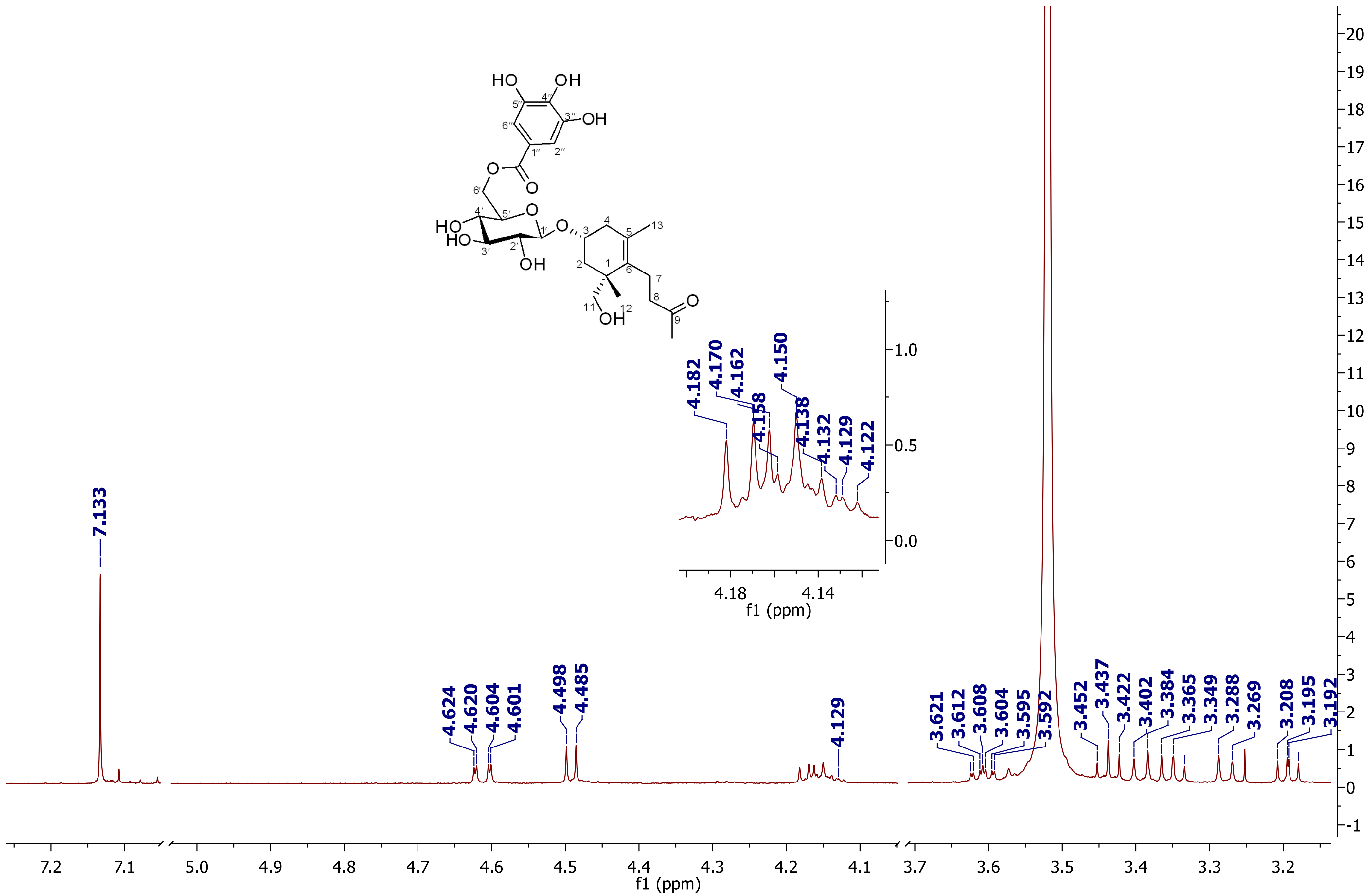

Figure S5. Expanded ^{1}H NMR spectrum of compound **1** [600 MHz, acetone-d_6 + D_2O (9+1)]

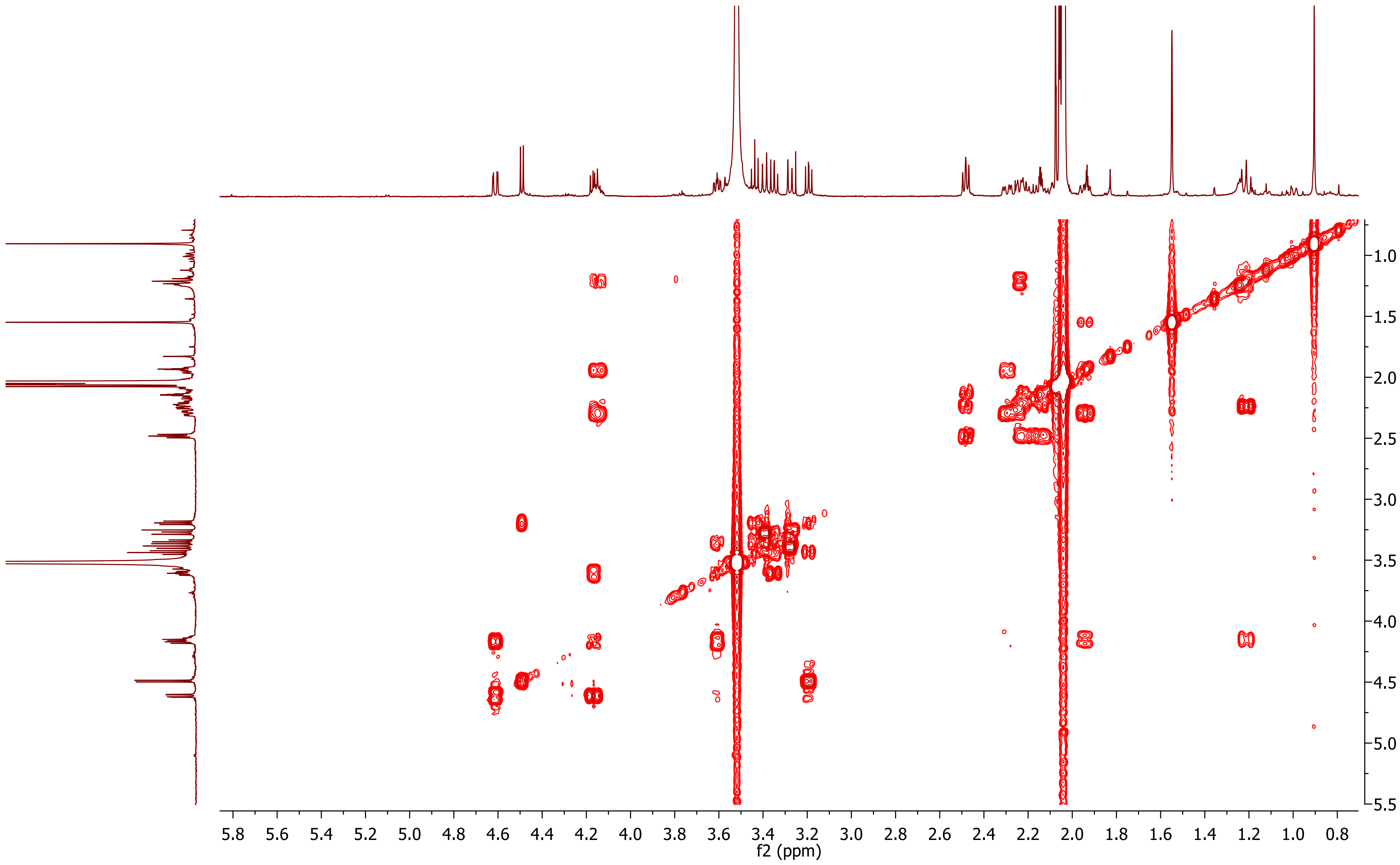

Figure S6. ^{1}H-^{1}H COSY spectrum of compound **1** [600 MHz, acetone-d_6 + D_2O (9+1)]

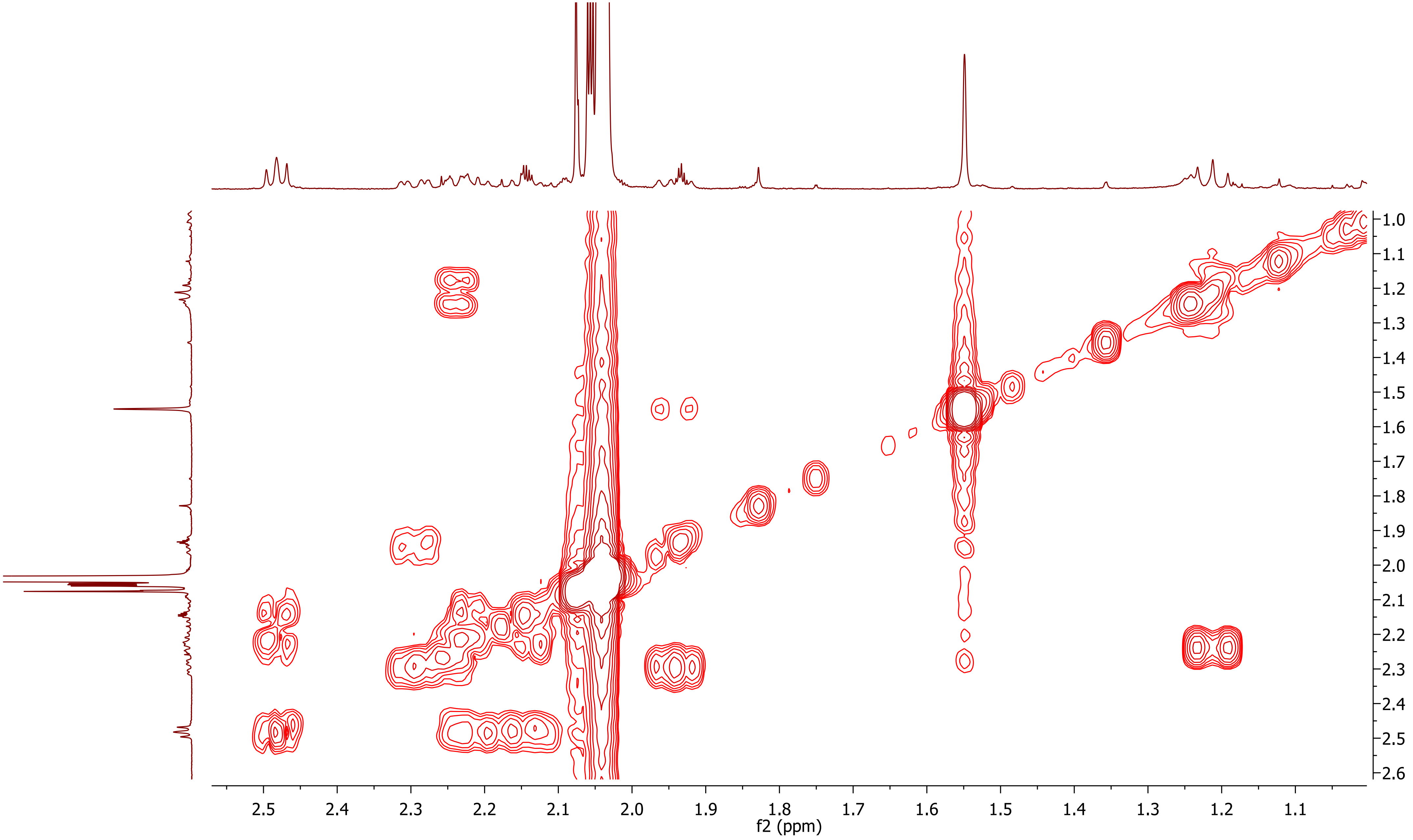

Figure S7. Expanded ^{1}H-^{1}H COSY spectrum of compound **1** [600 MHz, acetone-d_6 + D_2O (9+1)]

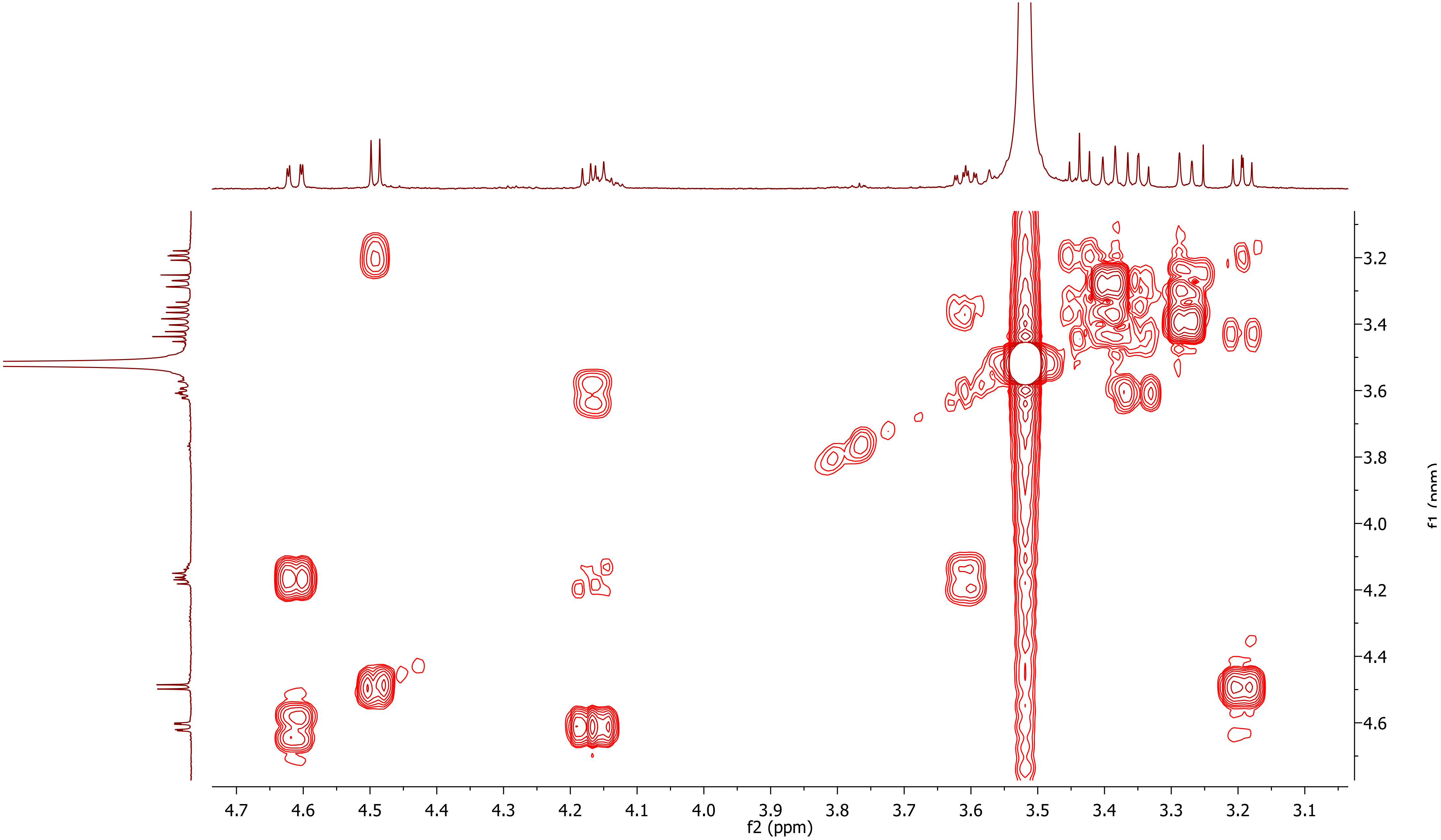

Figure S8. Expanded ^{1}H-^{1}H COSY spectrum of compound **1** [600 MHz, acetone-d_6 + D_2O (9+1)]

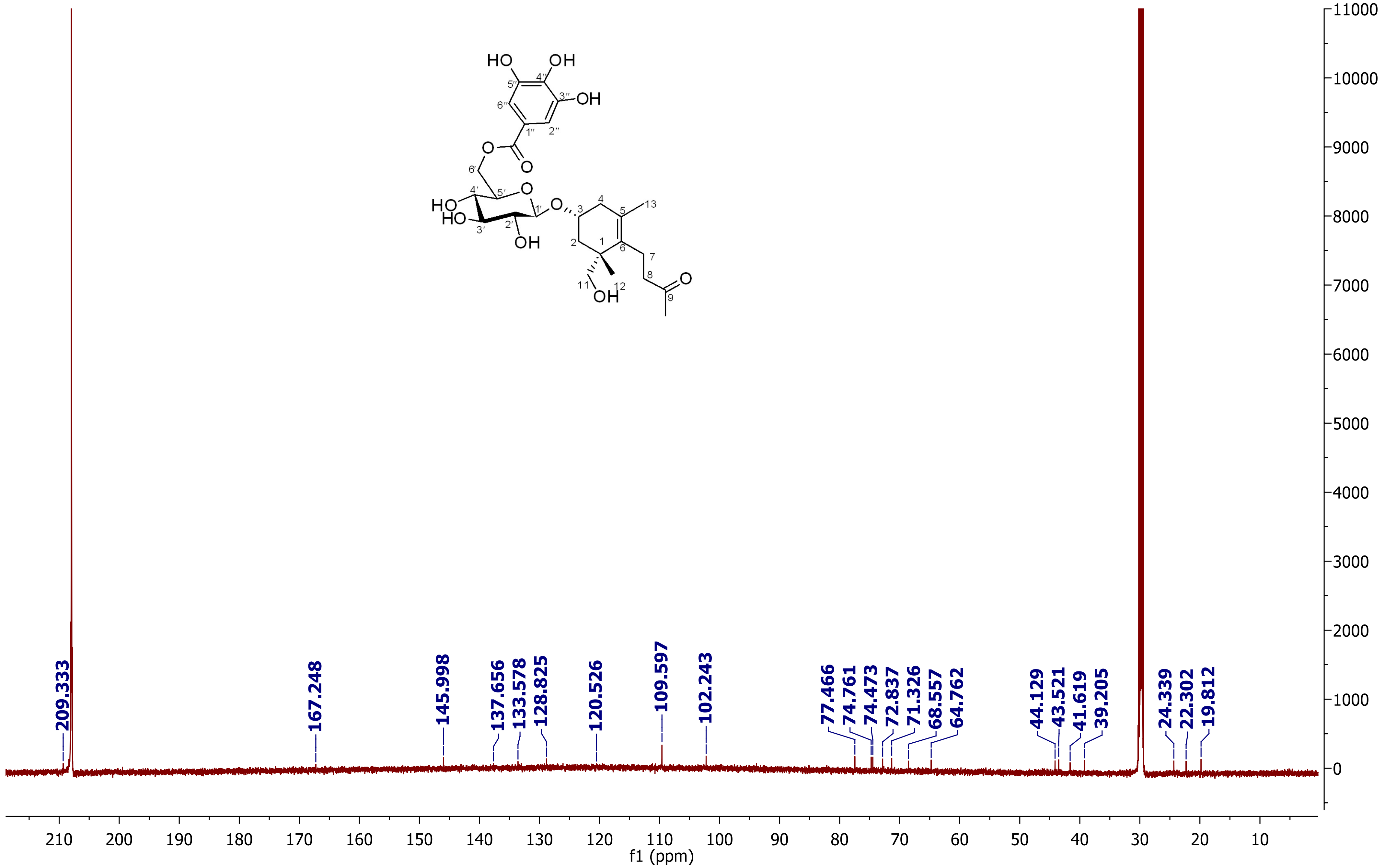

Figure S9. ^{13}C NMR spectrum of compound **1** [151 MHz, acetone-d_6 + D_2O (9+1)]

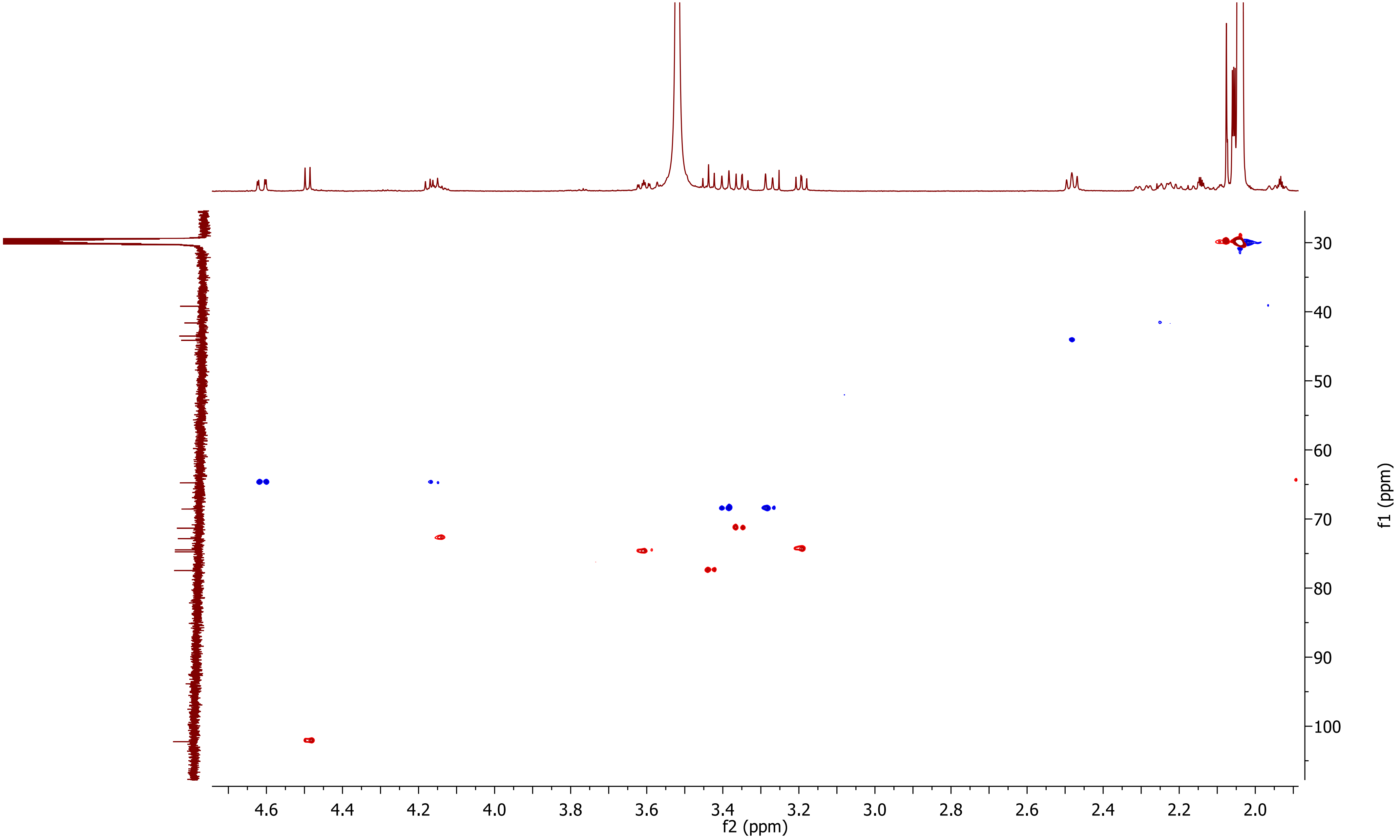

Figure S10. HSQC spectrum of compound **1** [600 MHz, acetone-d_6 + D_2O (9+1)]

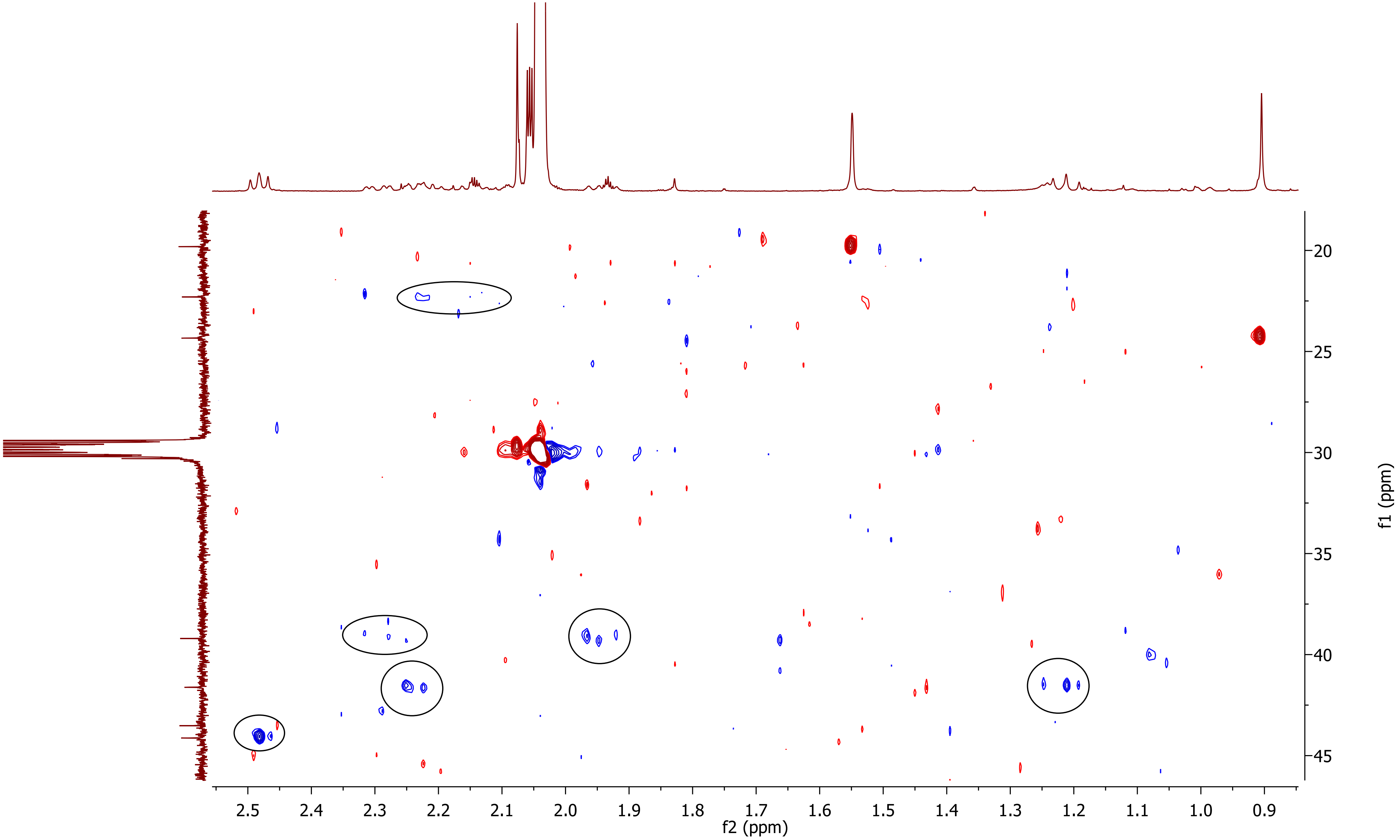

Figure S11. Expanded HSQC spectrum of compound **1** [600 MHz, acetone-d_6 + D_2O (9+1)]

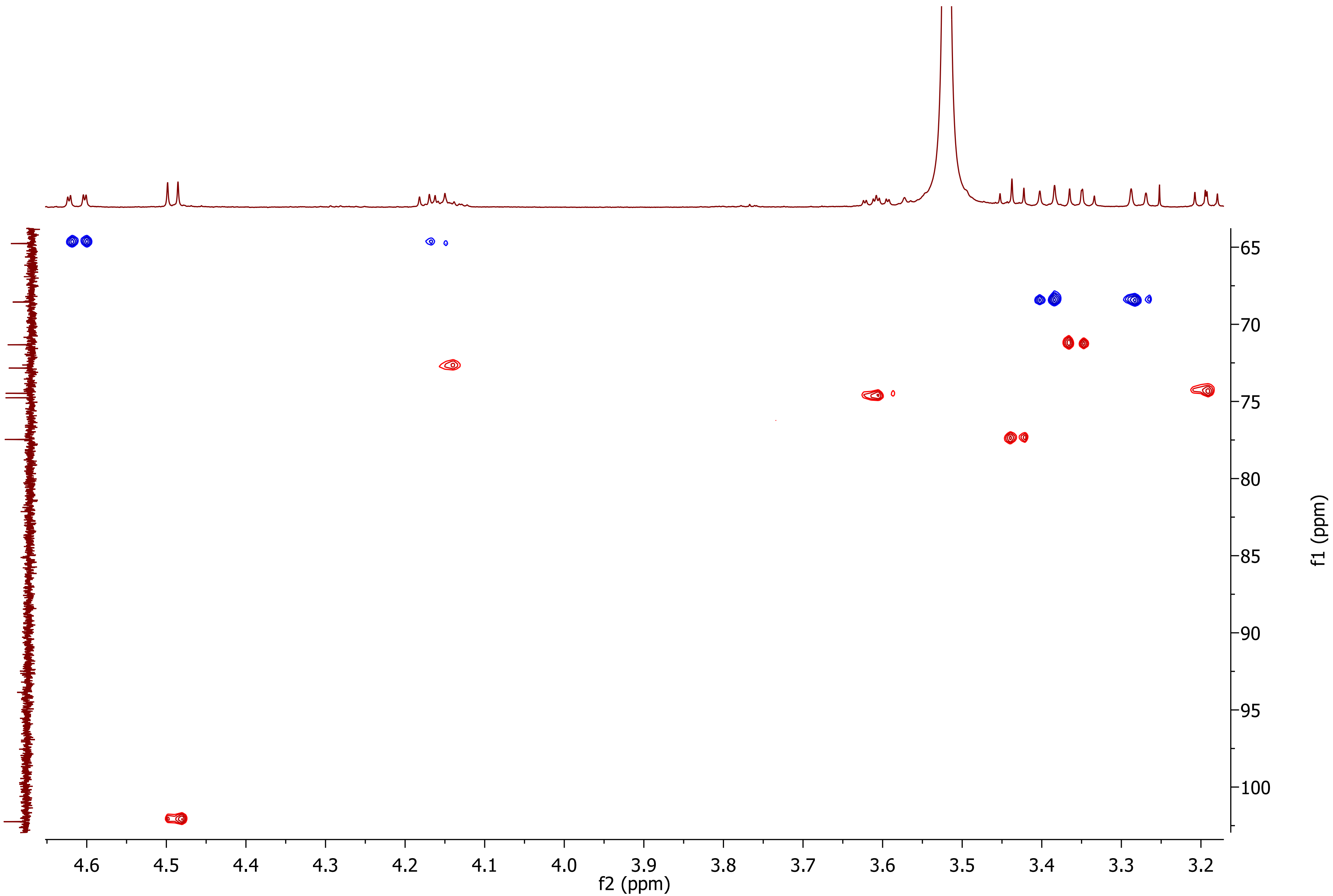

Figure S12. Expanded HSQC spectrum of compound **1** [600 MHz, acetone-d_6 + D_2O (9+1)]

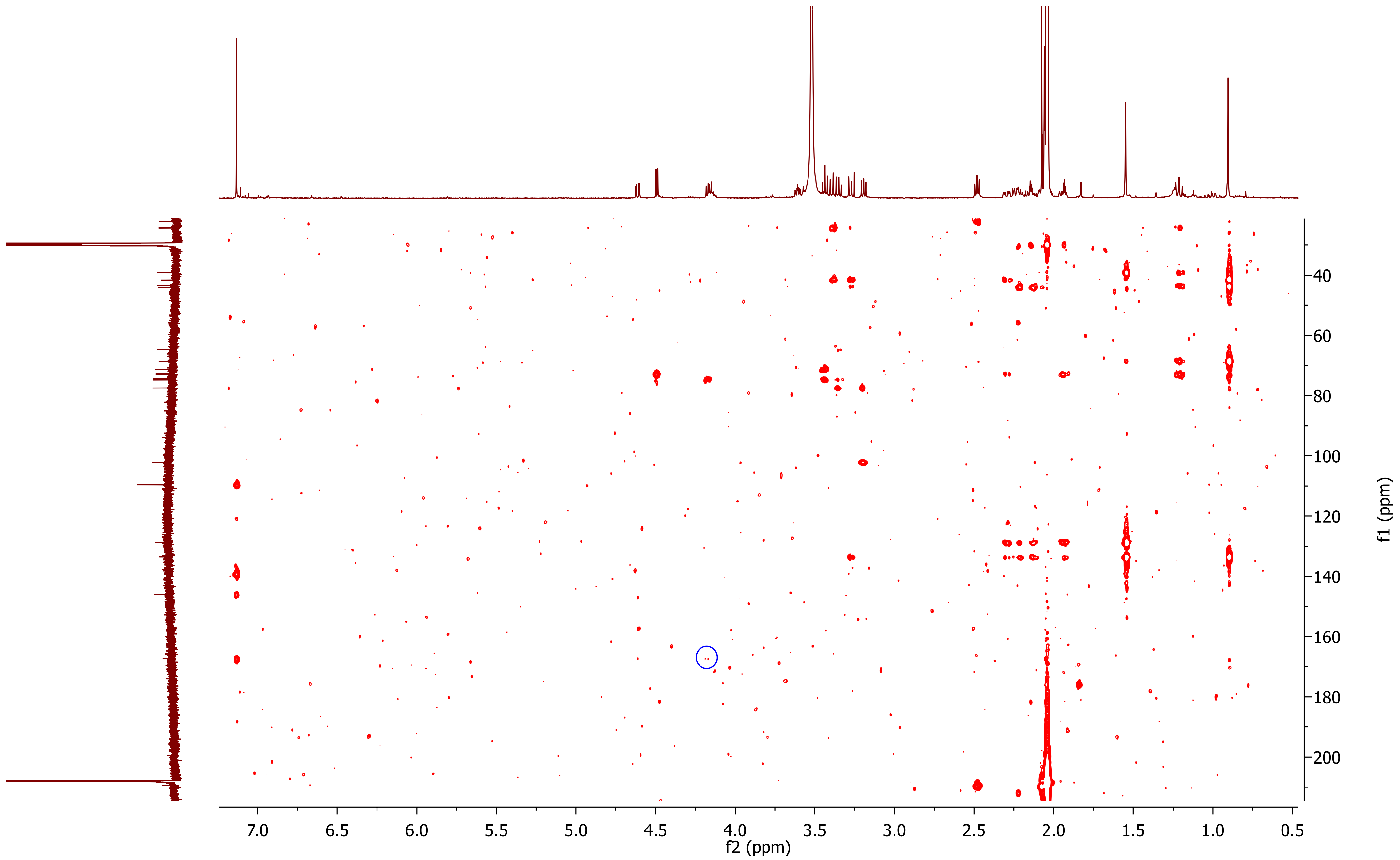

Figure S13. HMBC spectrum of compound **1** [600 MHz, acetone-d_6 + D_2O (9+1)]

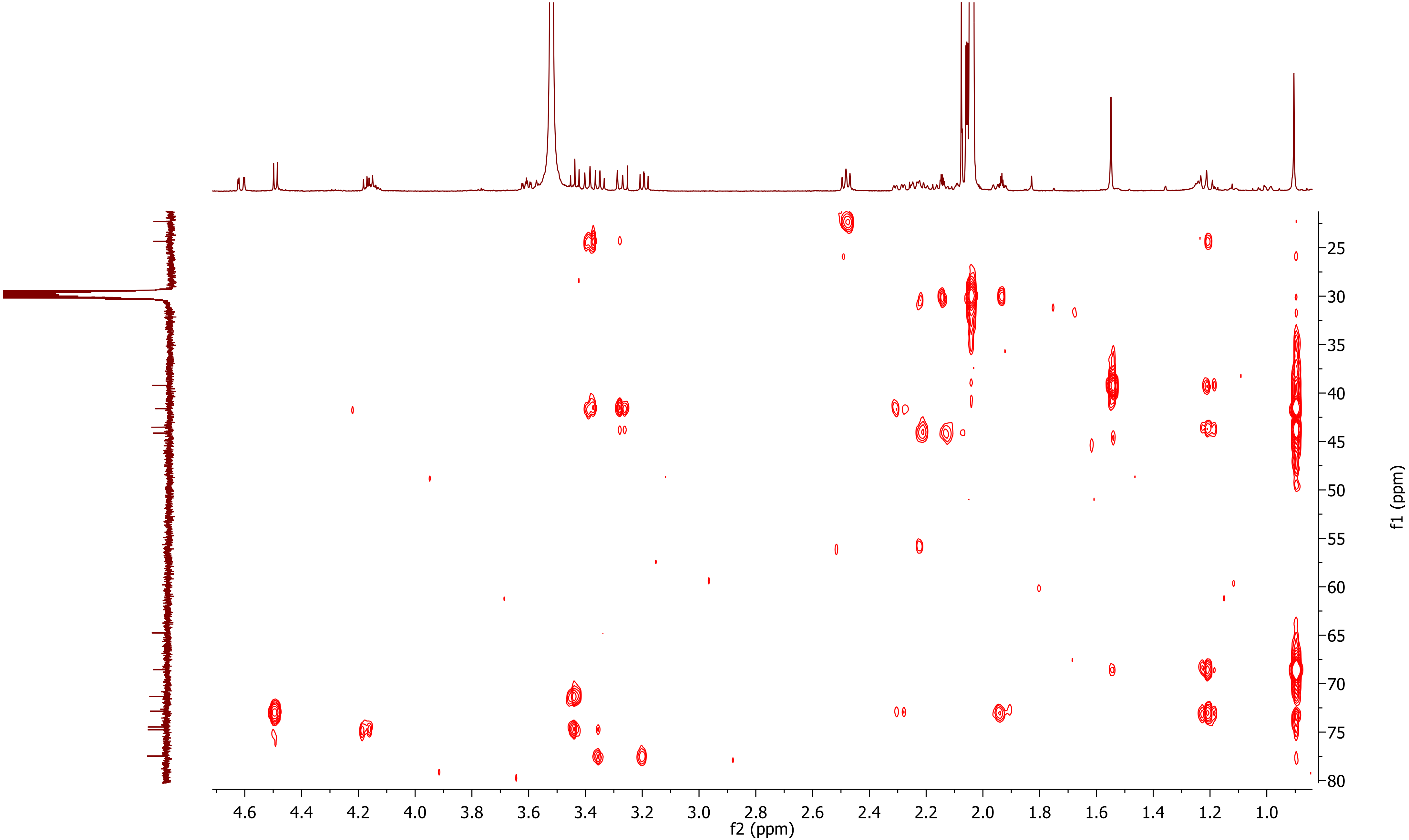

Figure S14. Expanded HMBC spectrum of compound **1** [600 MHz, acetone-d_6 + D_2O (9+1)]

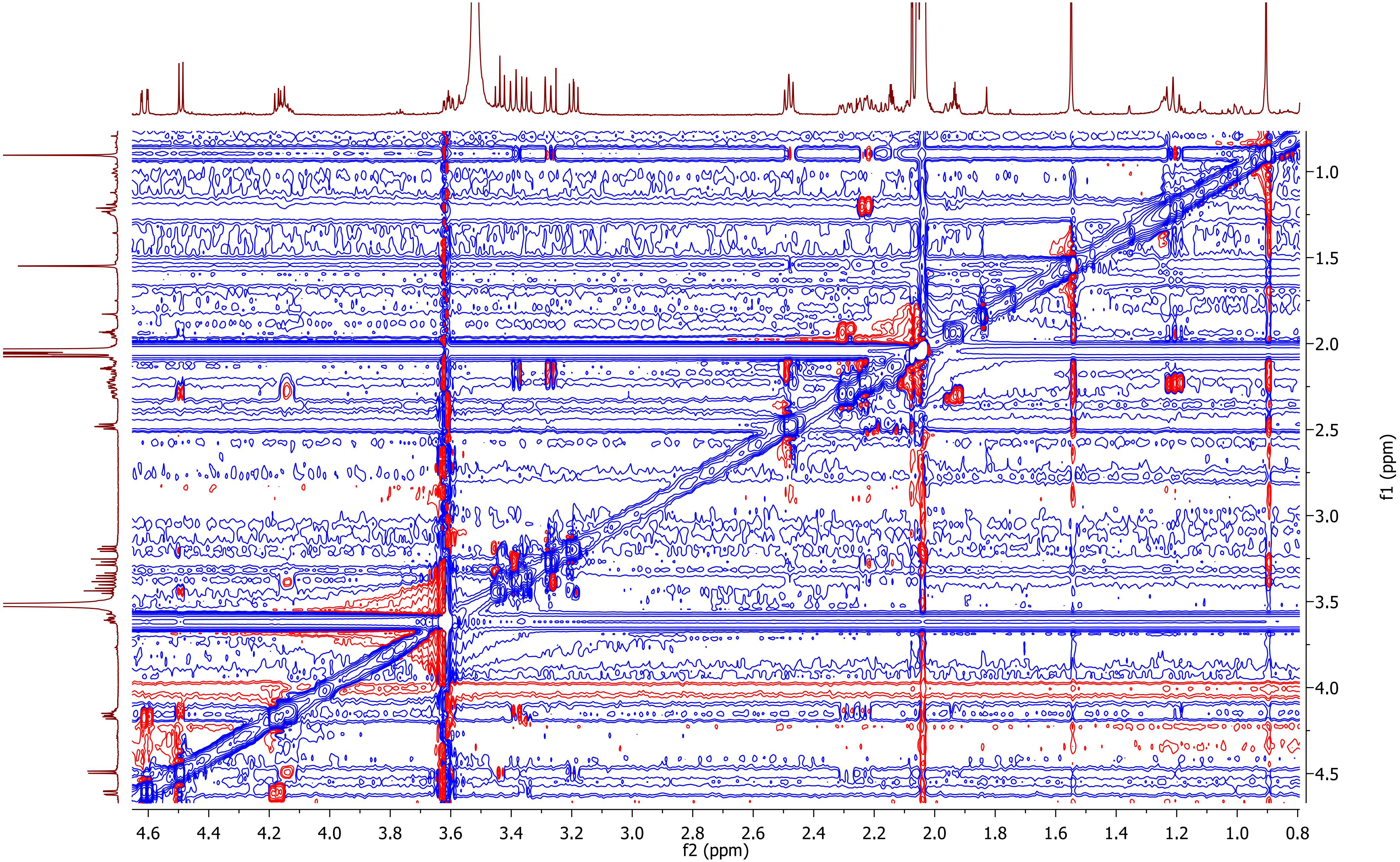

Figure S15. NOESY spectrum of compound **1** [600 MHz, acetone-d_6 + D_2O (9+1)]

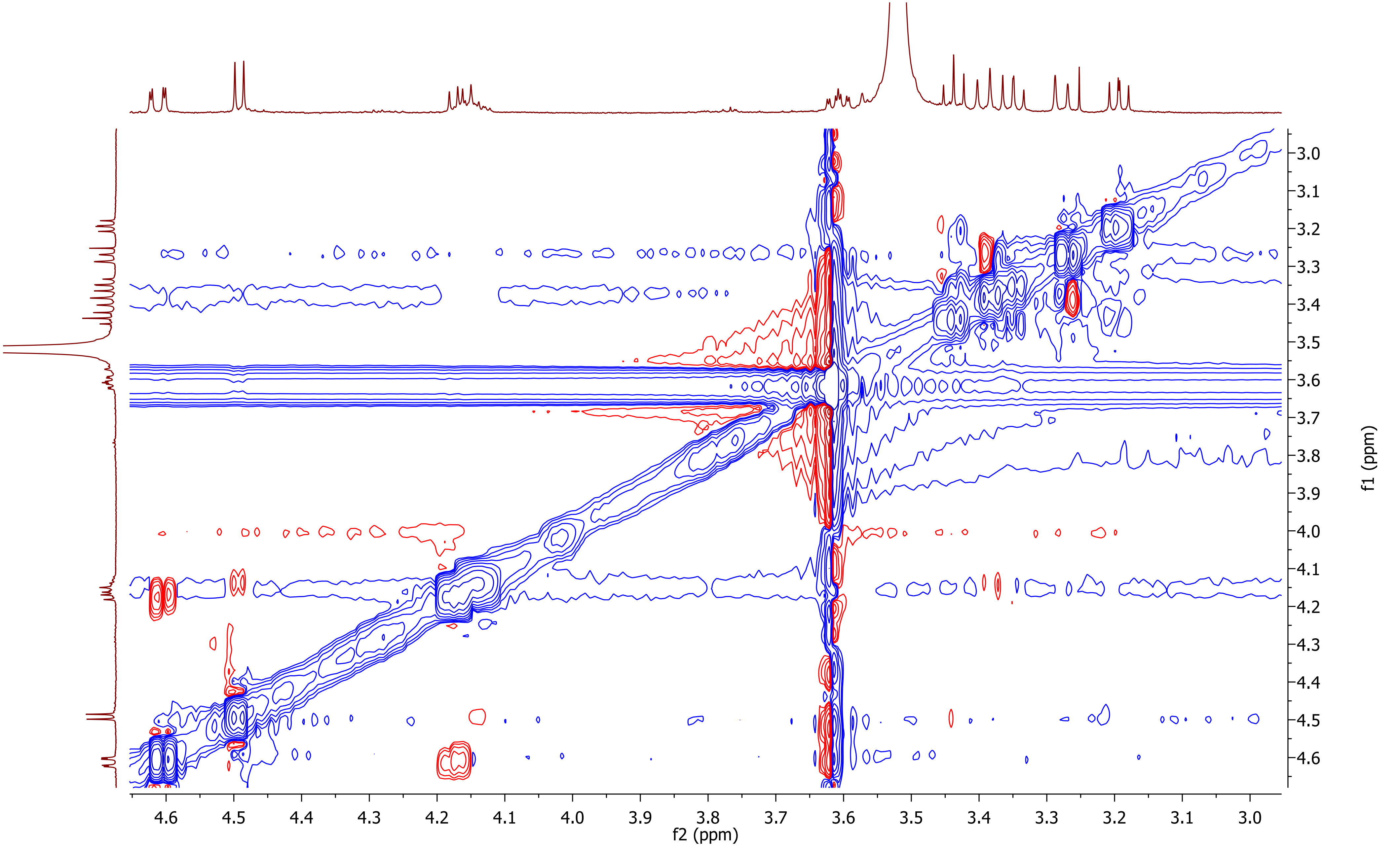

Figure S16. Expanded NOESY spectrum of compound **1** [600 MHz, acetone-d_6 + D_2O (9+1)]

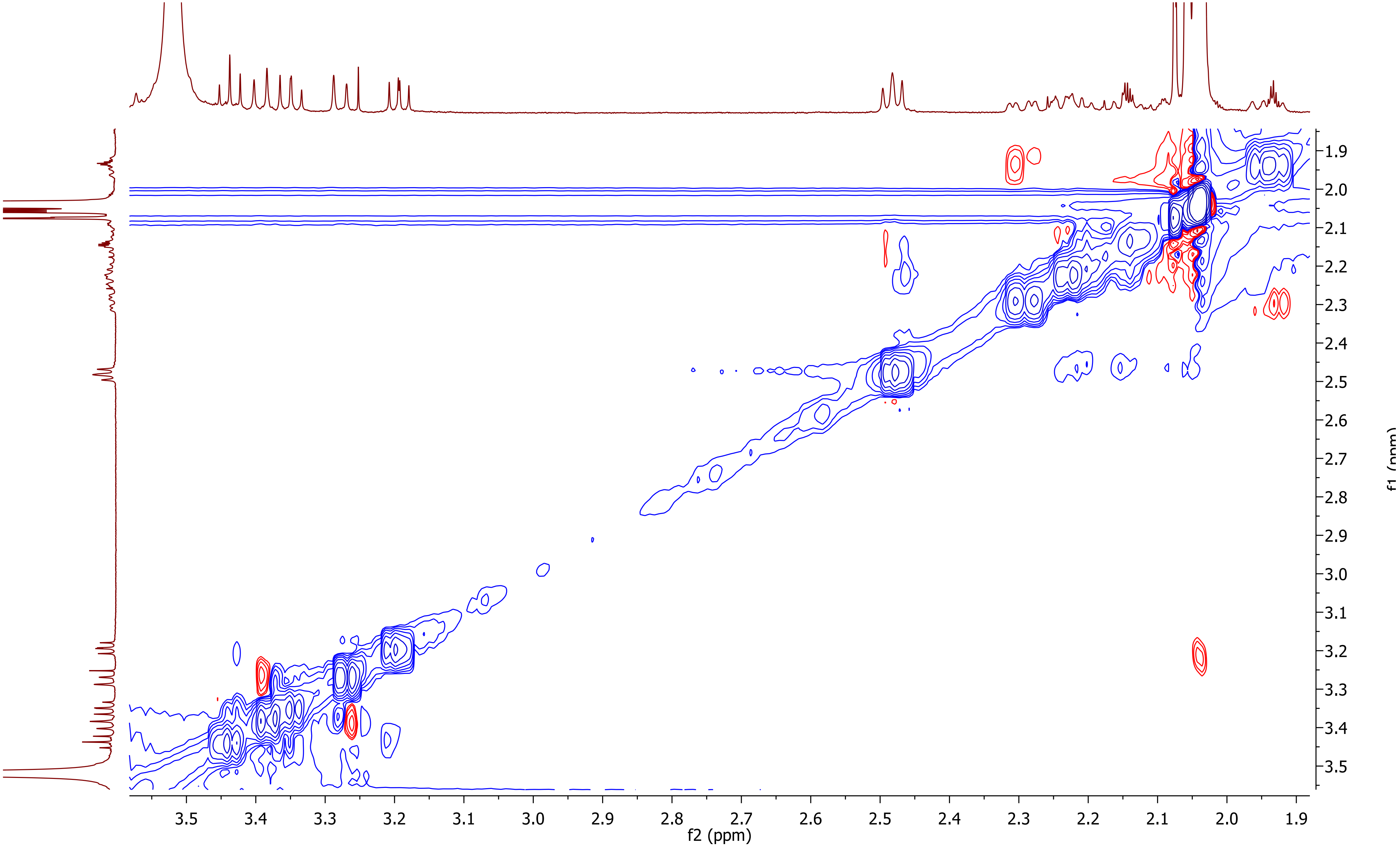

Figure S17. Expanded NOESY spectrum of compound **1** [600 MHz, acetone-d_6 + D_2O (9+1)]

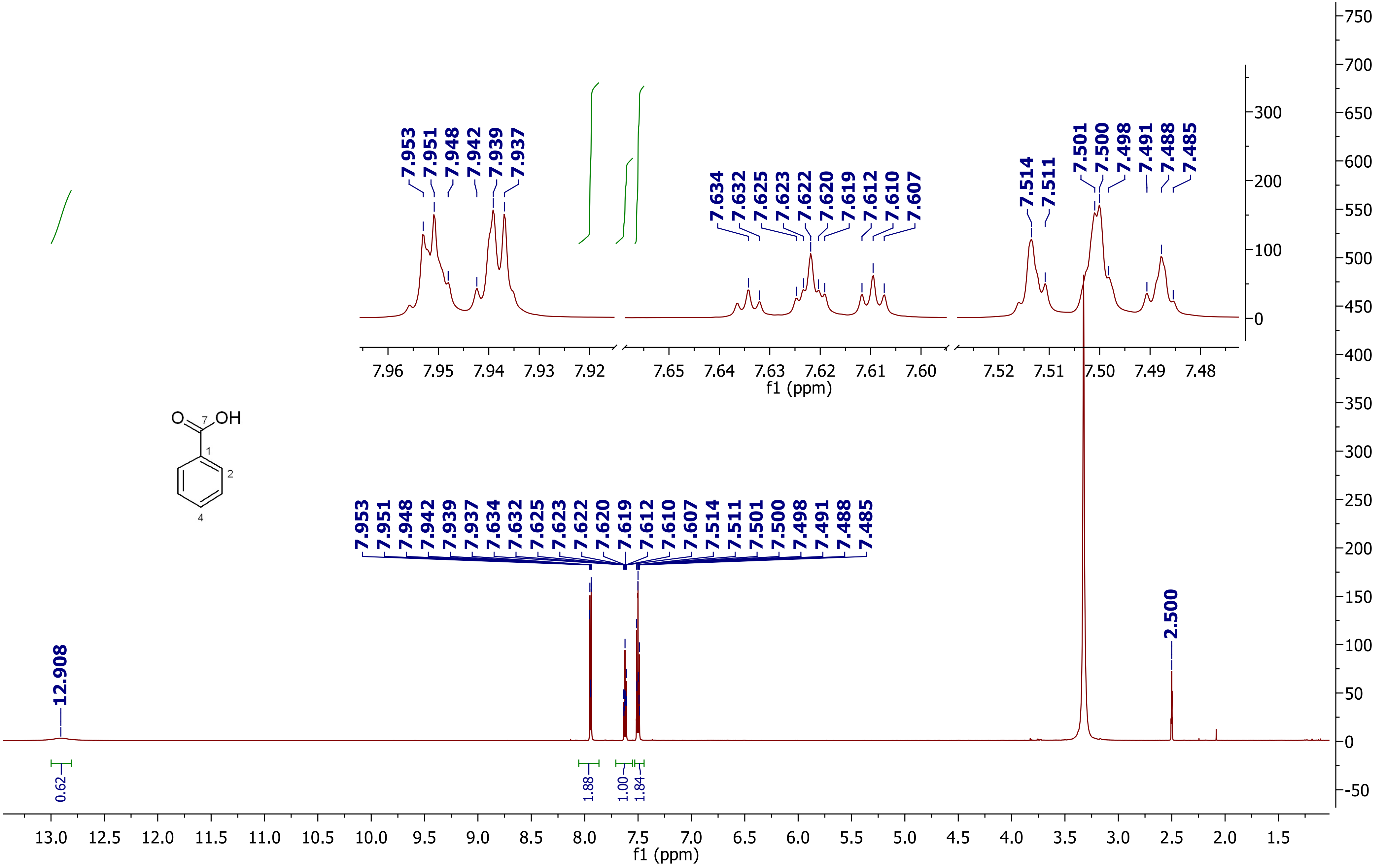

Figure S18. ^{1}H NMR spectrum of compound **2** (600 MHz, DMSO-d_6)

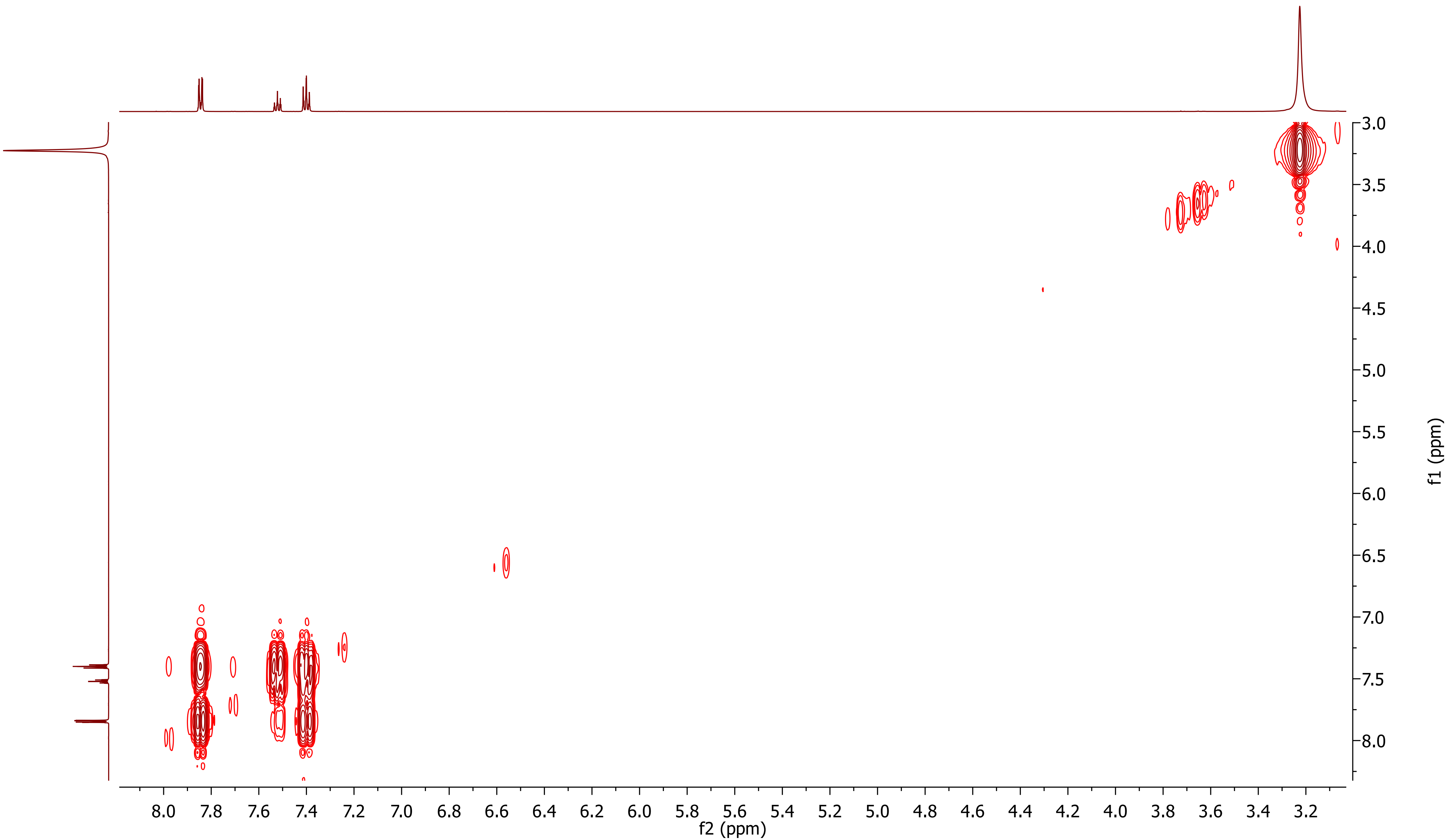

Figure S19. ^{1}H-^{1}H COSY spectrum of compound **2** (600 MHz, DMSO-d_6)

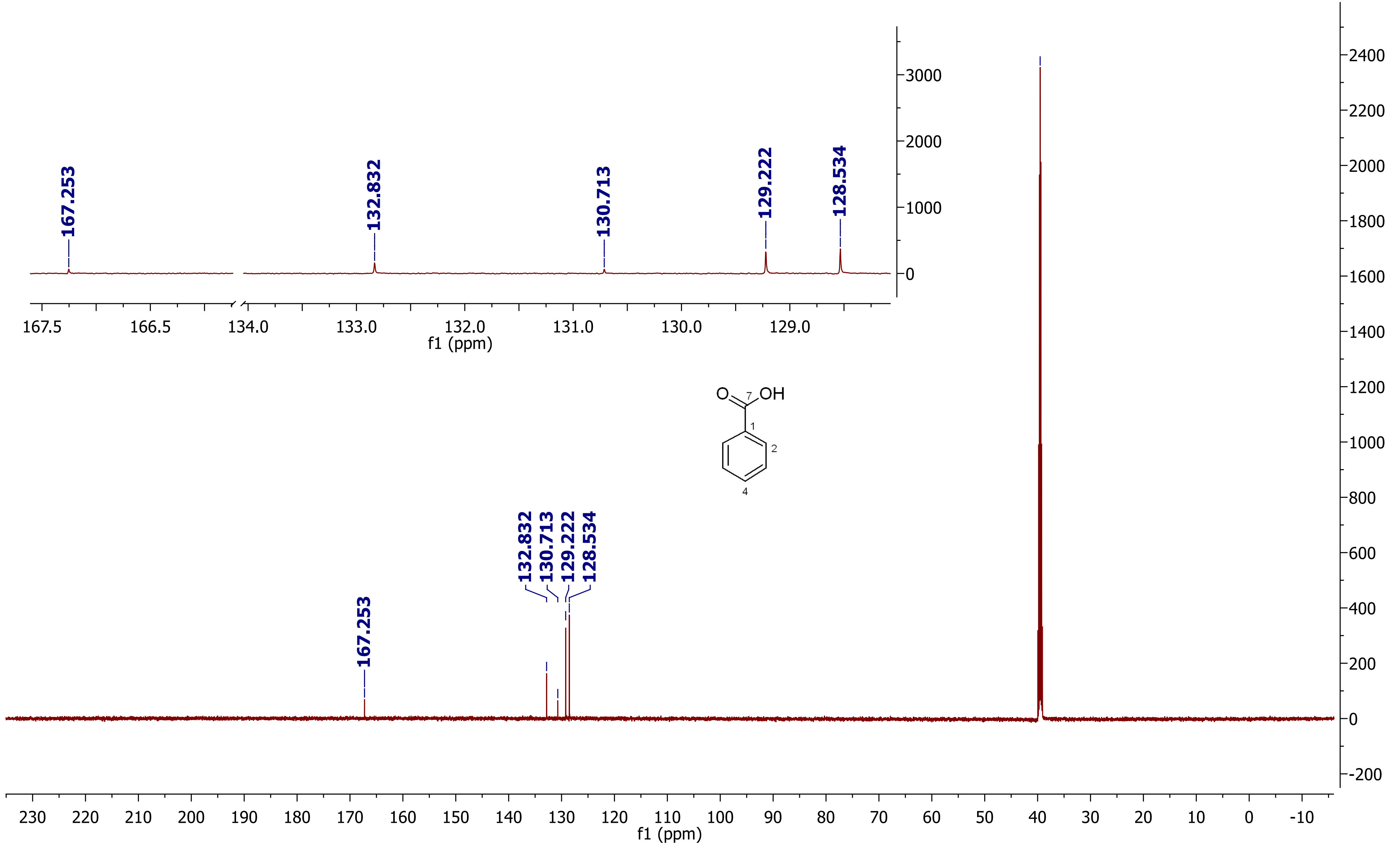

Figure S20. ^{13}C NMR spectrum of compound **2** (151 MHz, DMSO-d_6)

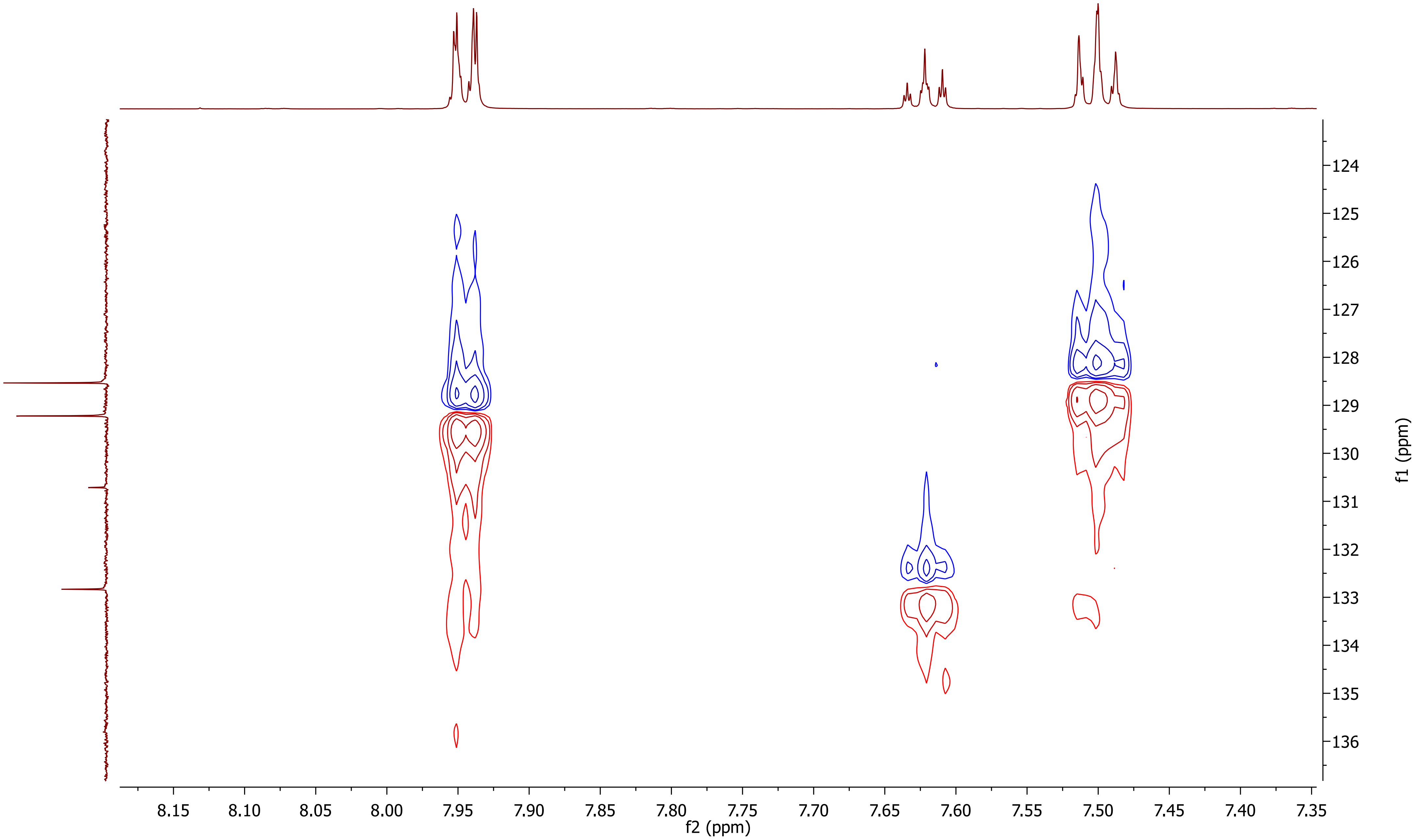

Figure S21. HSQC spectrum of compound **2** (600 MHz, DMSO-d_6)

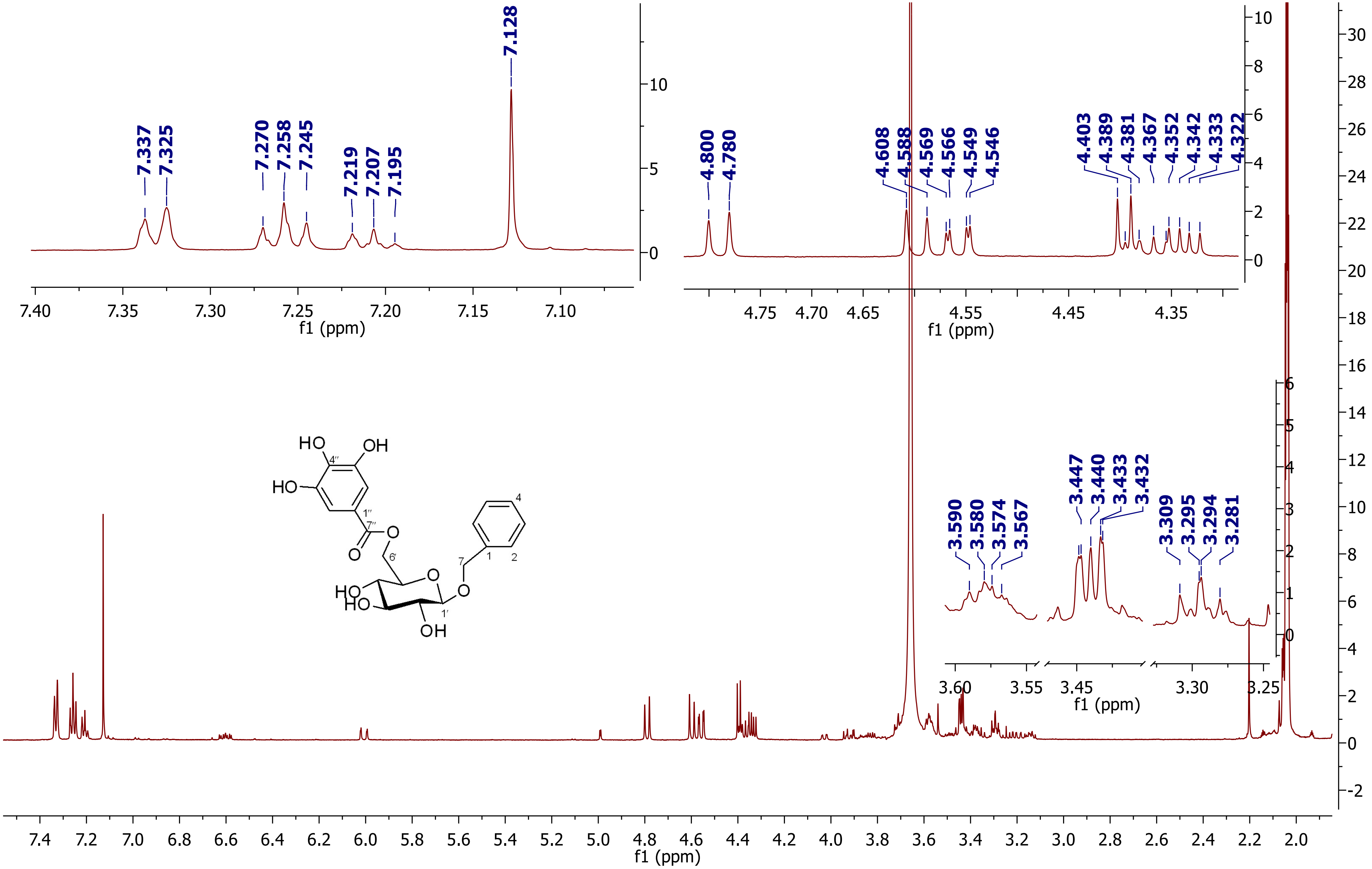

Figure S22. ^{1}H NMR spectrum of compound **3** [600 MHz, acetone-d_6 + D_2O (9+1)]

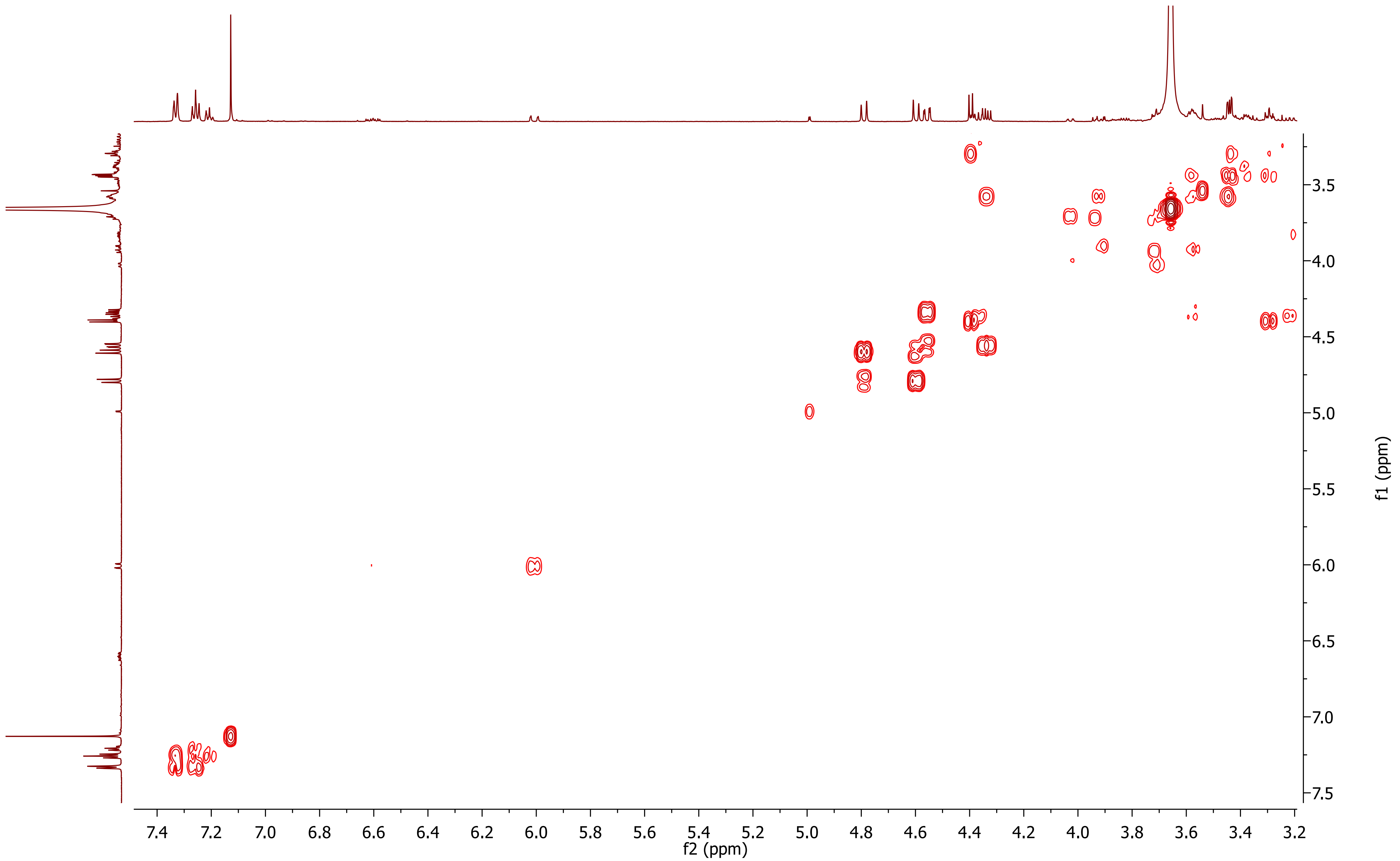

Figure S23. ^{1}H-^{1}H COSY spectrum of compound **3** [600 MHz, acetone-d_6 + D_2O (9+1)]

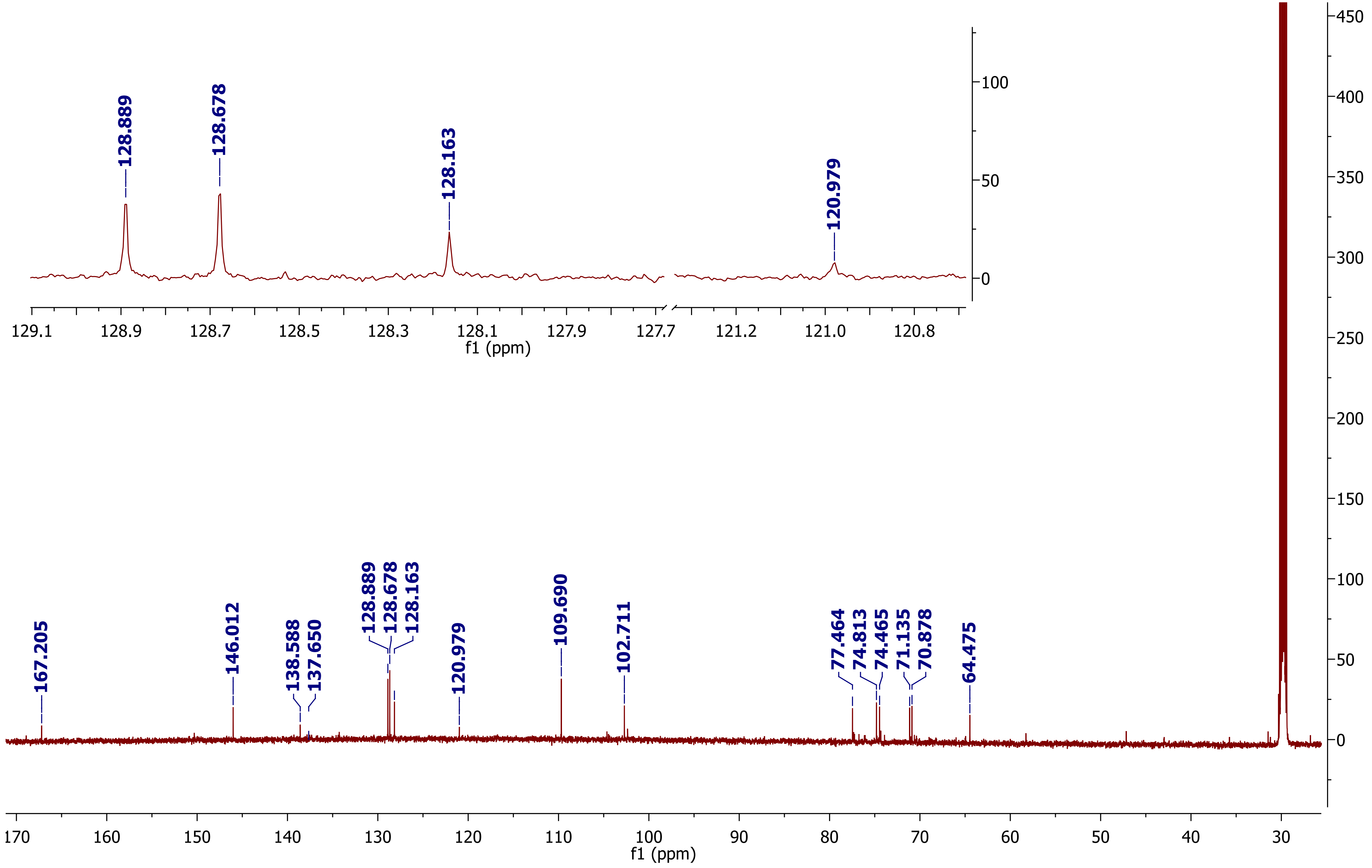

Figure S24. ^{13}C NMR spectrum of compound 3**1** [151 MHz, acetone-d_6 + D_2O (9+1)]

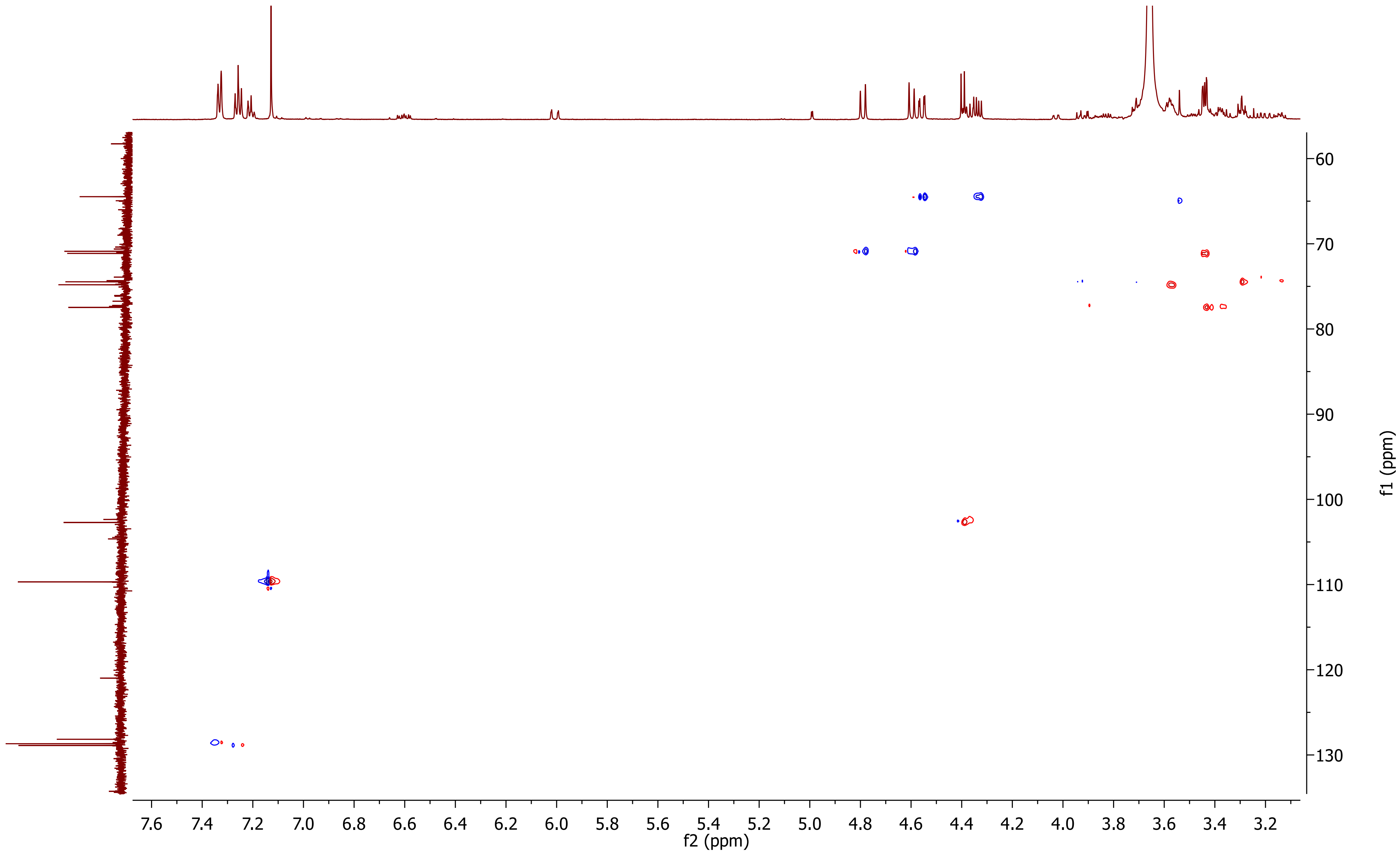

Figure S25. HSQC spectrum of compound **3** [600 MHz, acetone-d_6 + D_2O (9+1)]

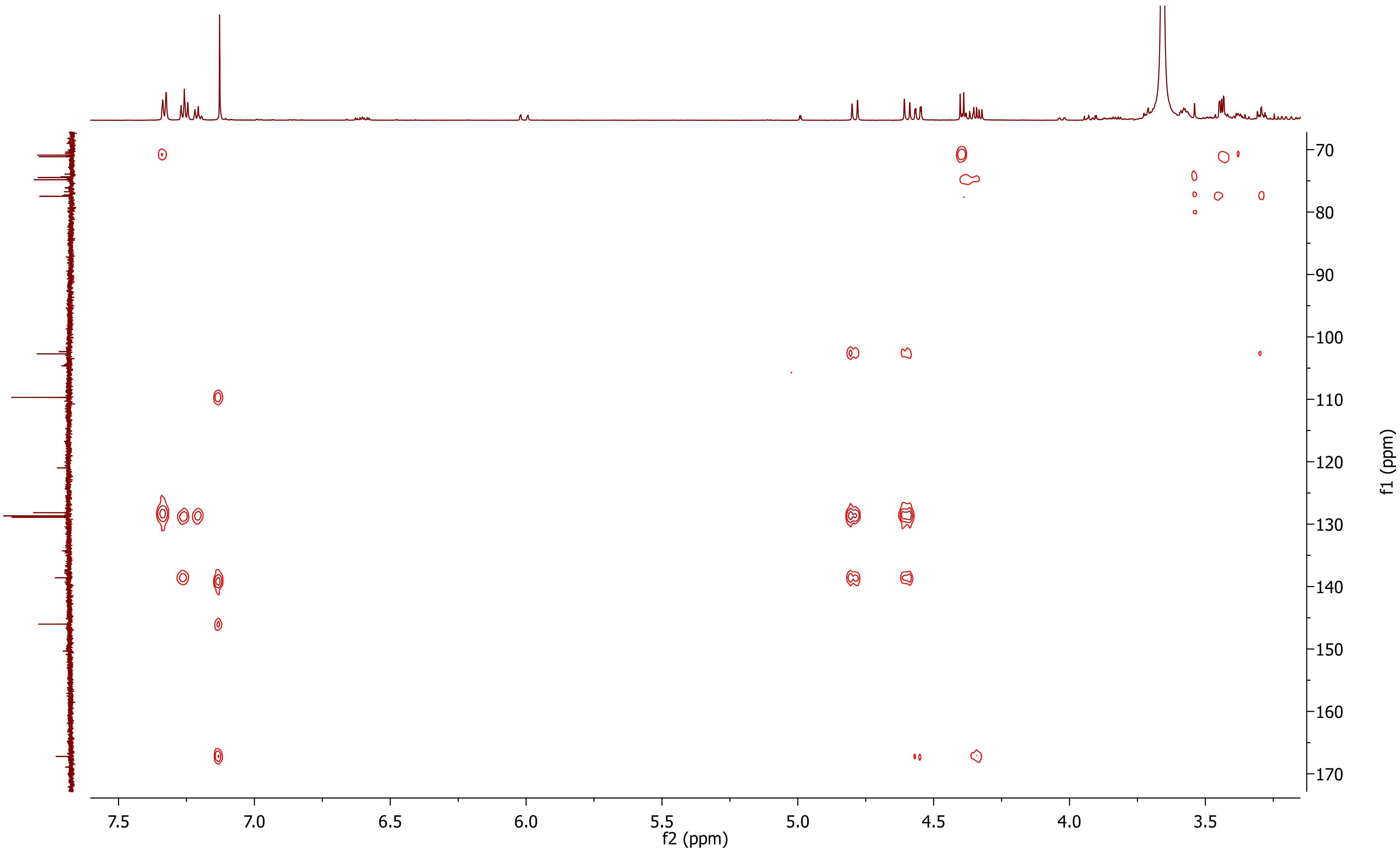

Figure S26. HMBC spectrum of compound **3** [600 MHz, acetone-d_6 + D_2O (9+1)]

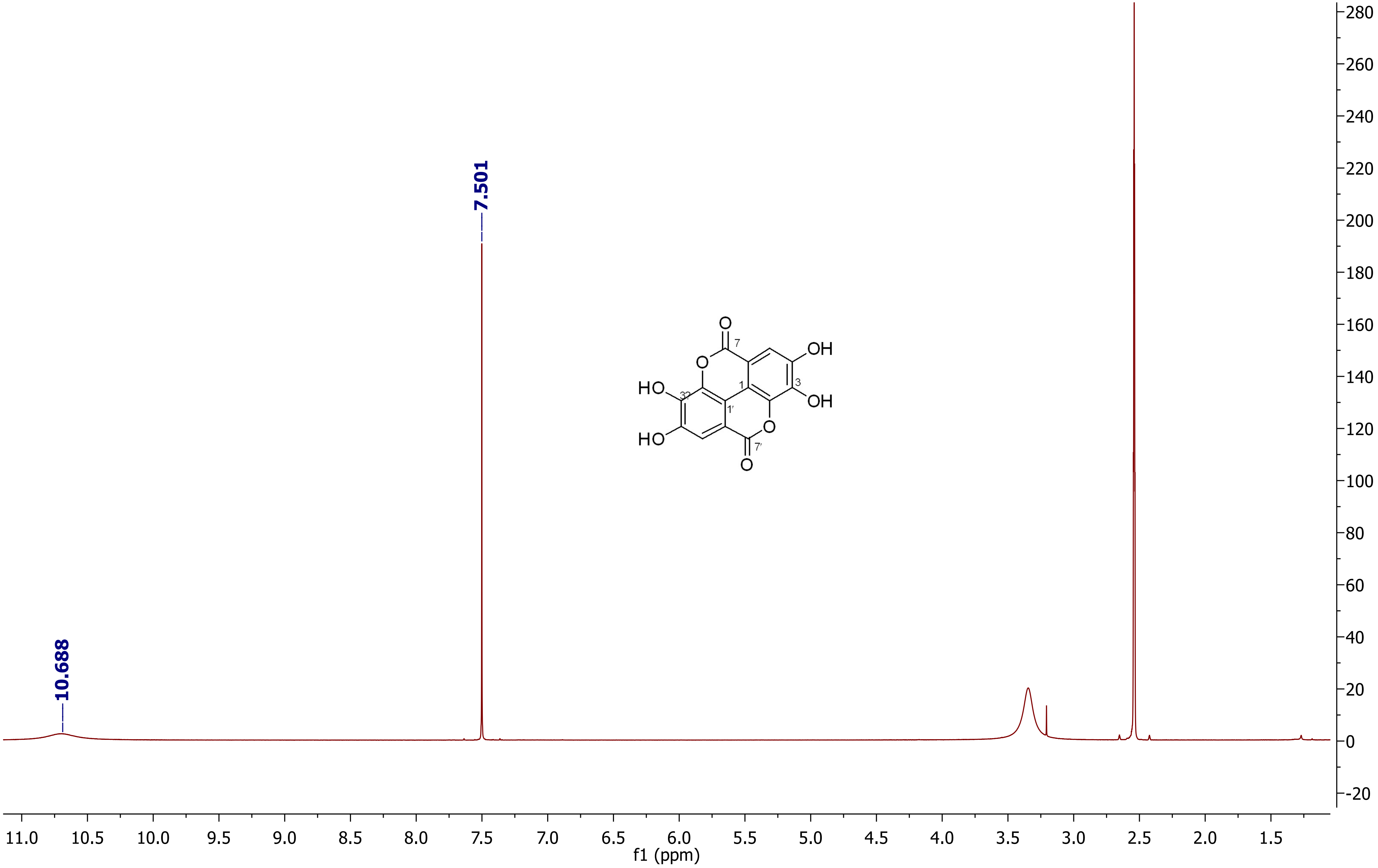

Figure S27. ^{1}H NMR spectrum of compound **4** (600 MHz, DMSO-d_6)

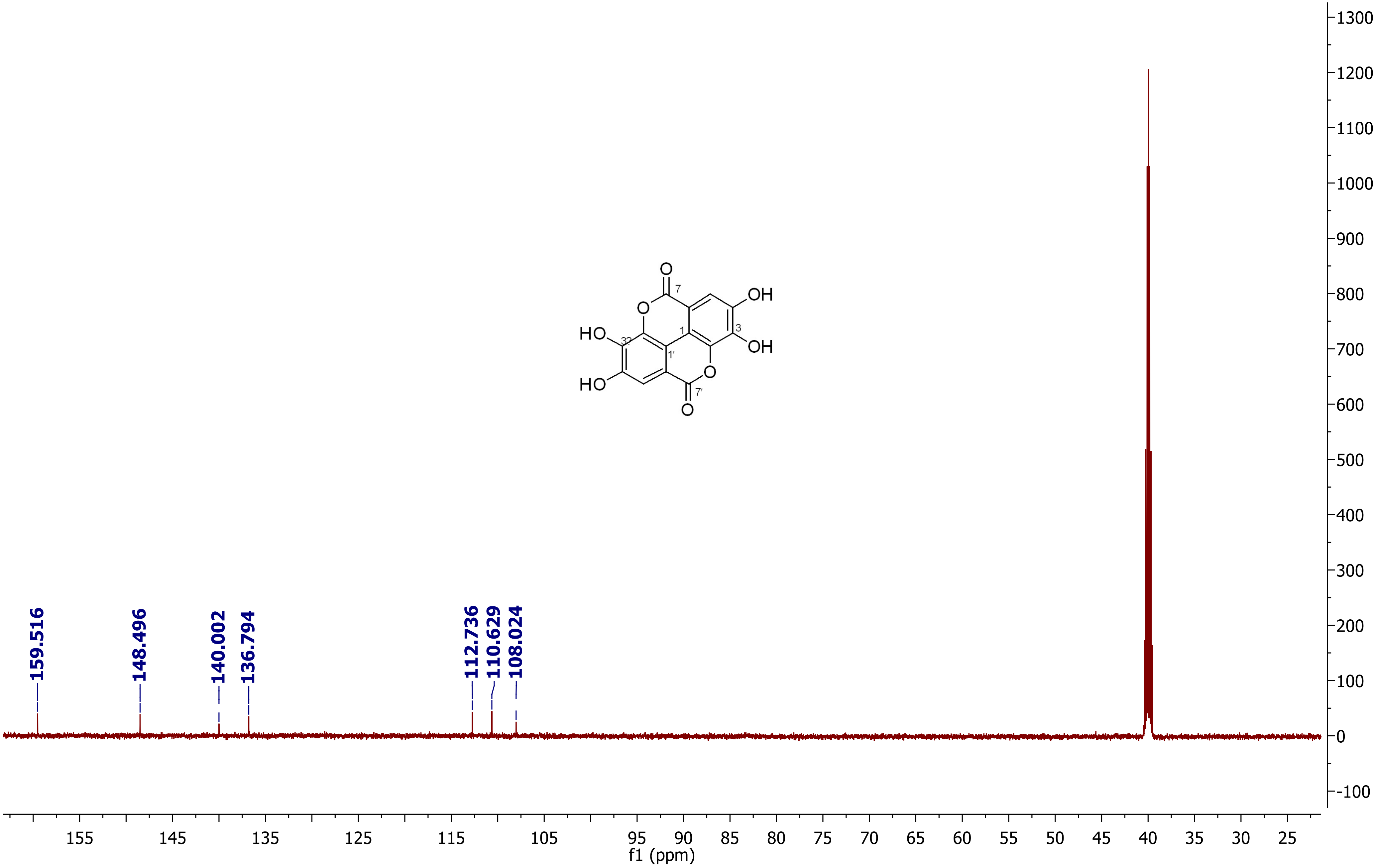

Figure S28. ^{13}C NMR spectrum of compound **4** (151 MHz, DMSO-d_6)

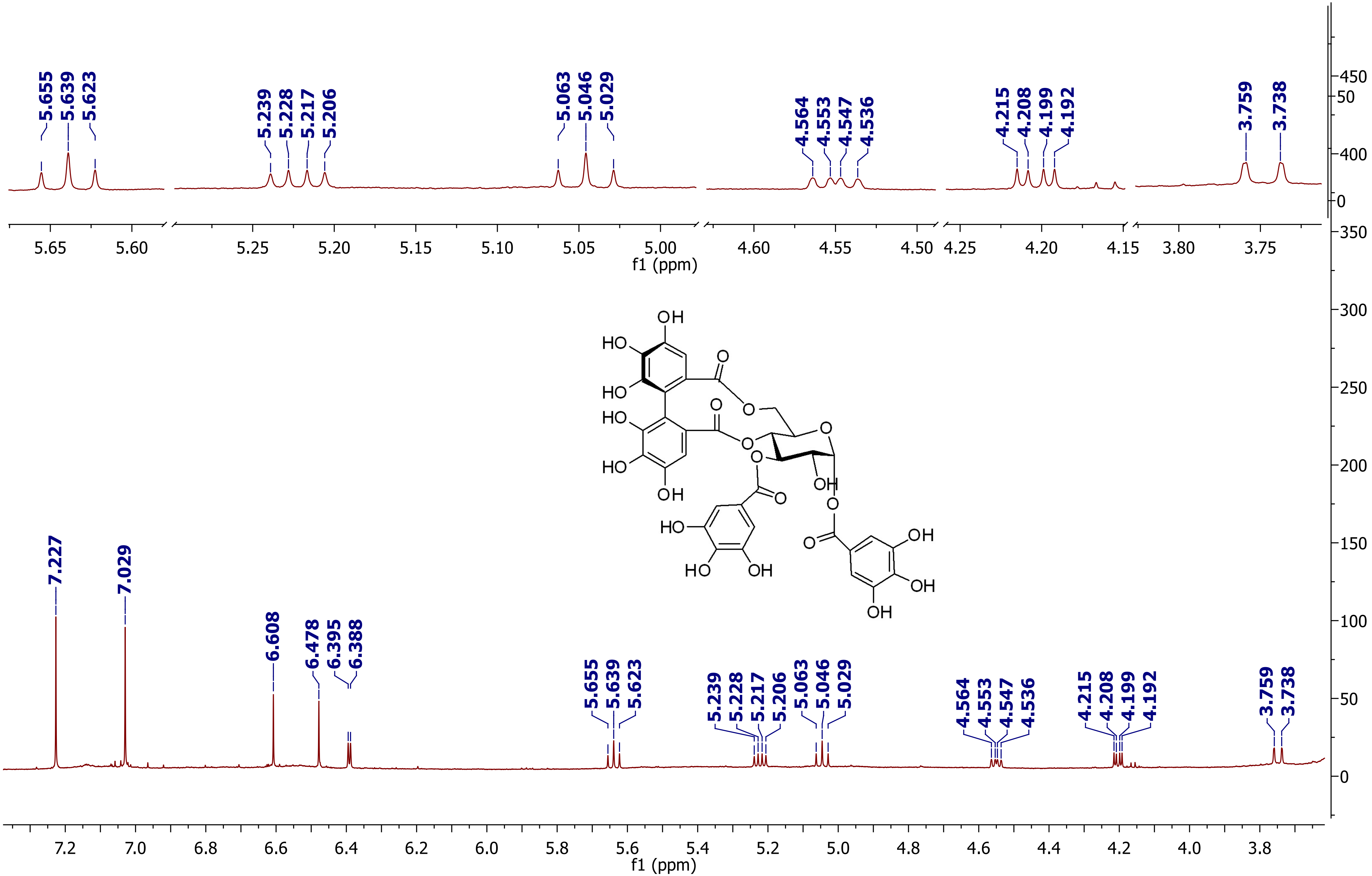

Figure S29. ^{1}H NMR spectrum of compound **5** [600 MHz, acetone-d_6 + D_2O (9+1)]

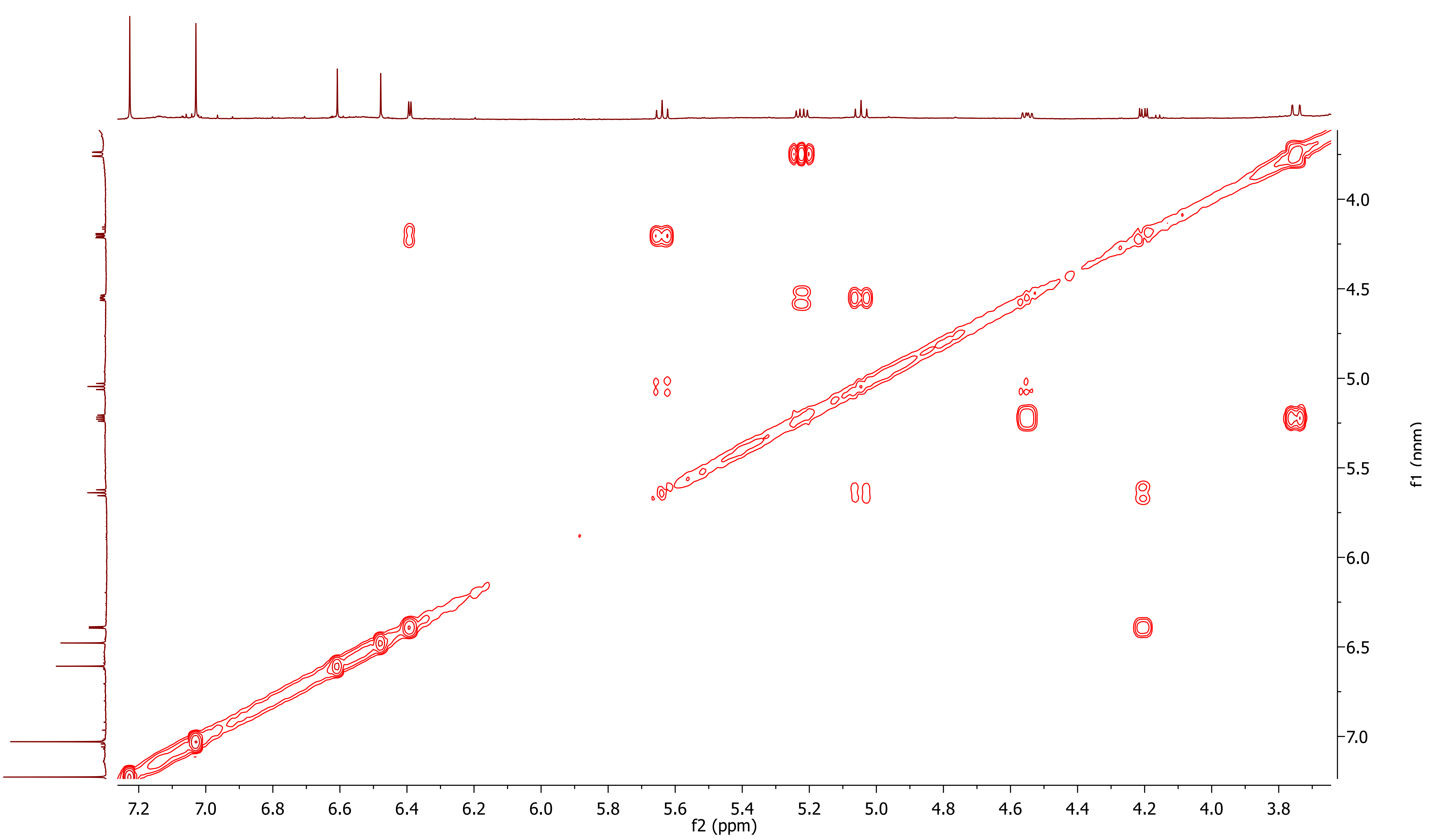

Figure S30. ^{1}H-^{1}H COSY spectrum of compound **5** [600 MHz, acetone-d_6 + D_2O (9+1)]

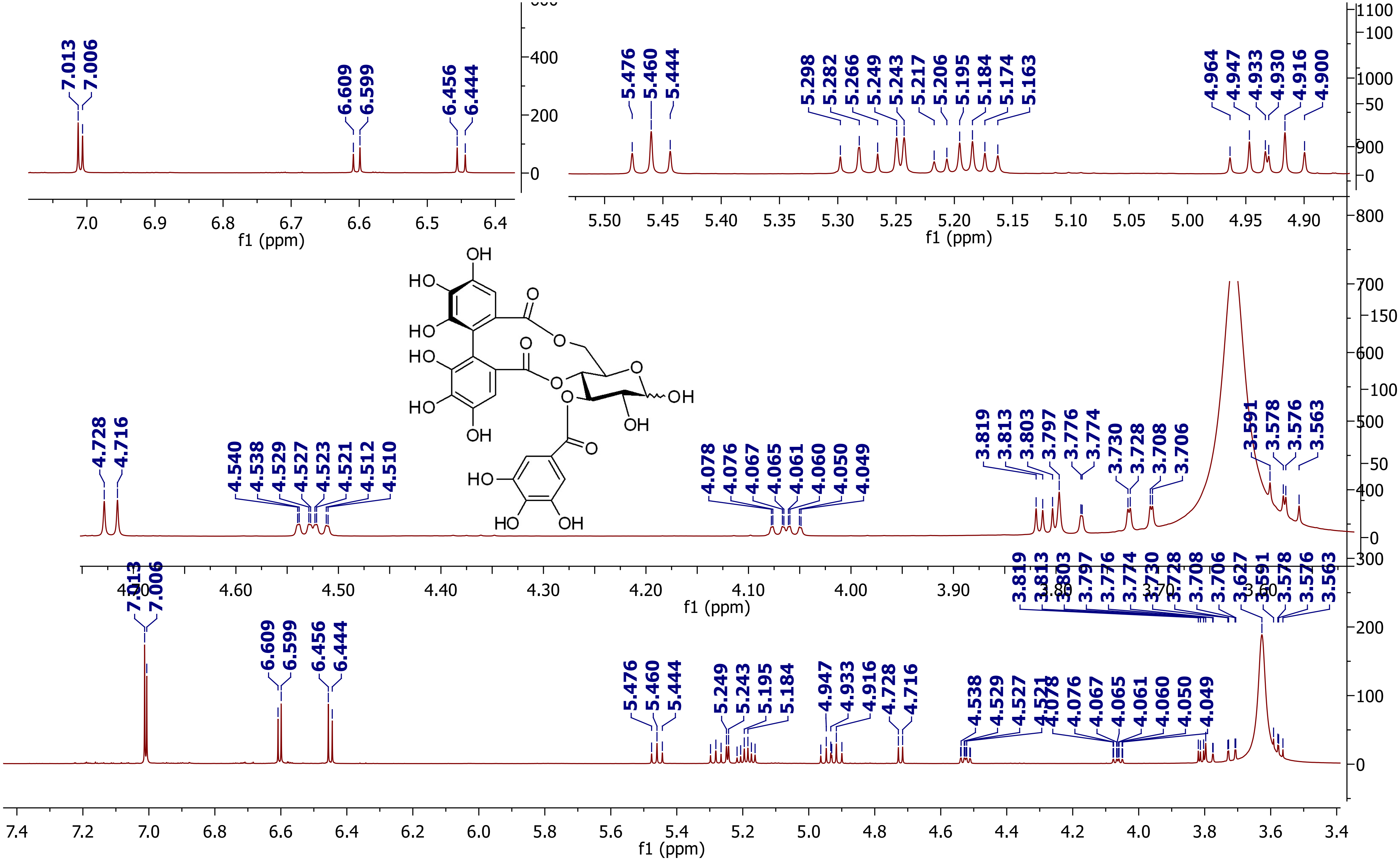

Figure S31. ^{1}H NMR spectrum of compound **6** [600 MHz, acetone-d_6 + D_2O (9+1)]

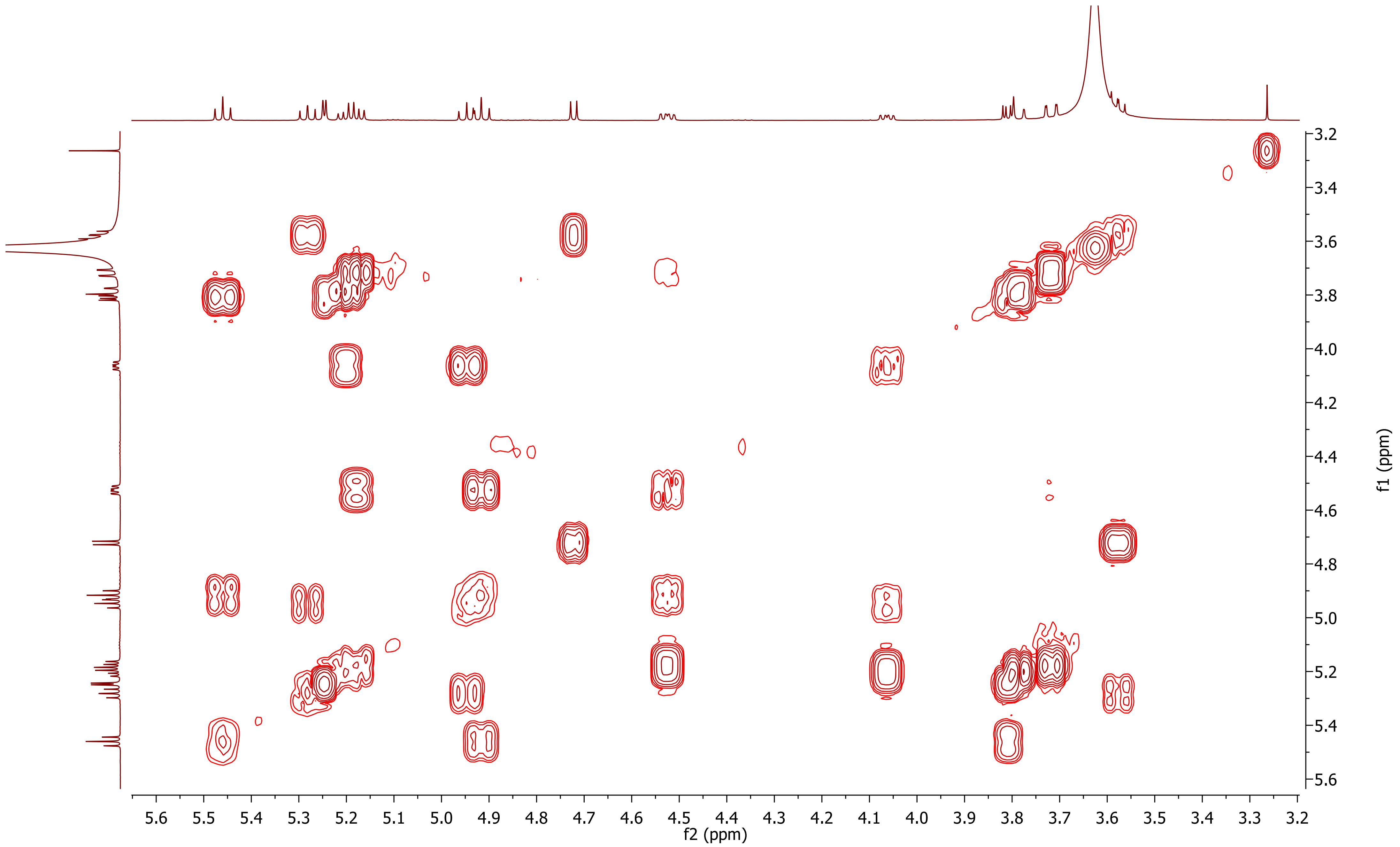

Figure S32. ^1H-^1H COSY spectrum of compound **6** [600 MHz, acetone-d_6 + D_2O (9+1)]

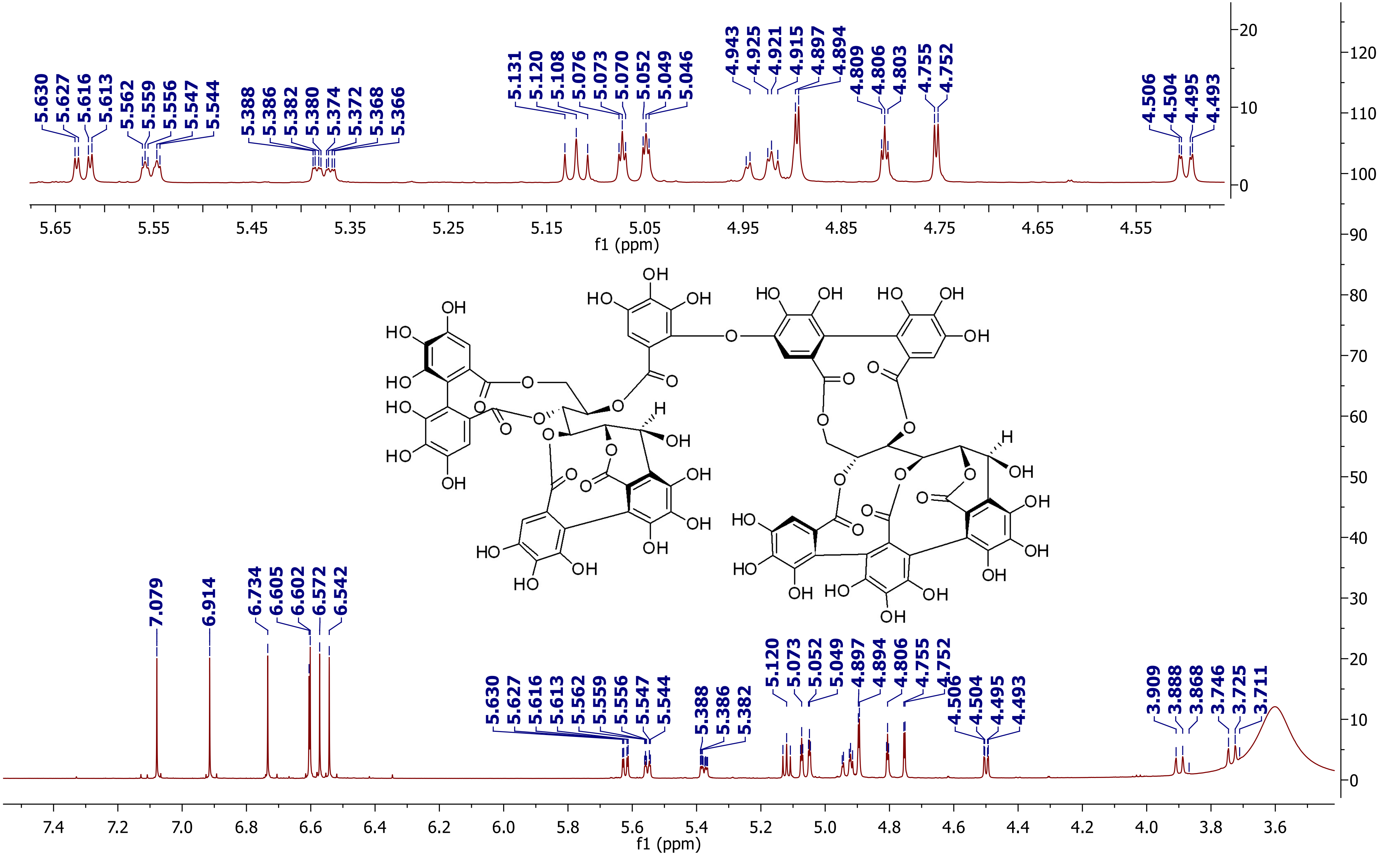

Figure S33. ^{1}H NMR spectrum of compound **7** [600 MHz, acetone-d_6 + D_2O (9+1)]

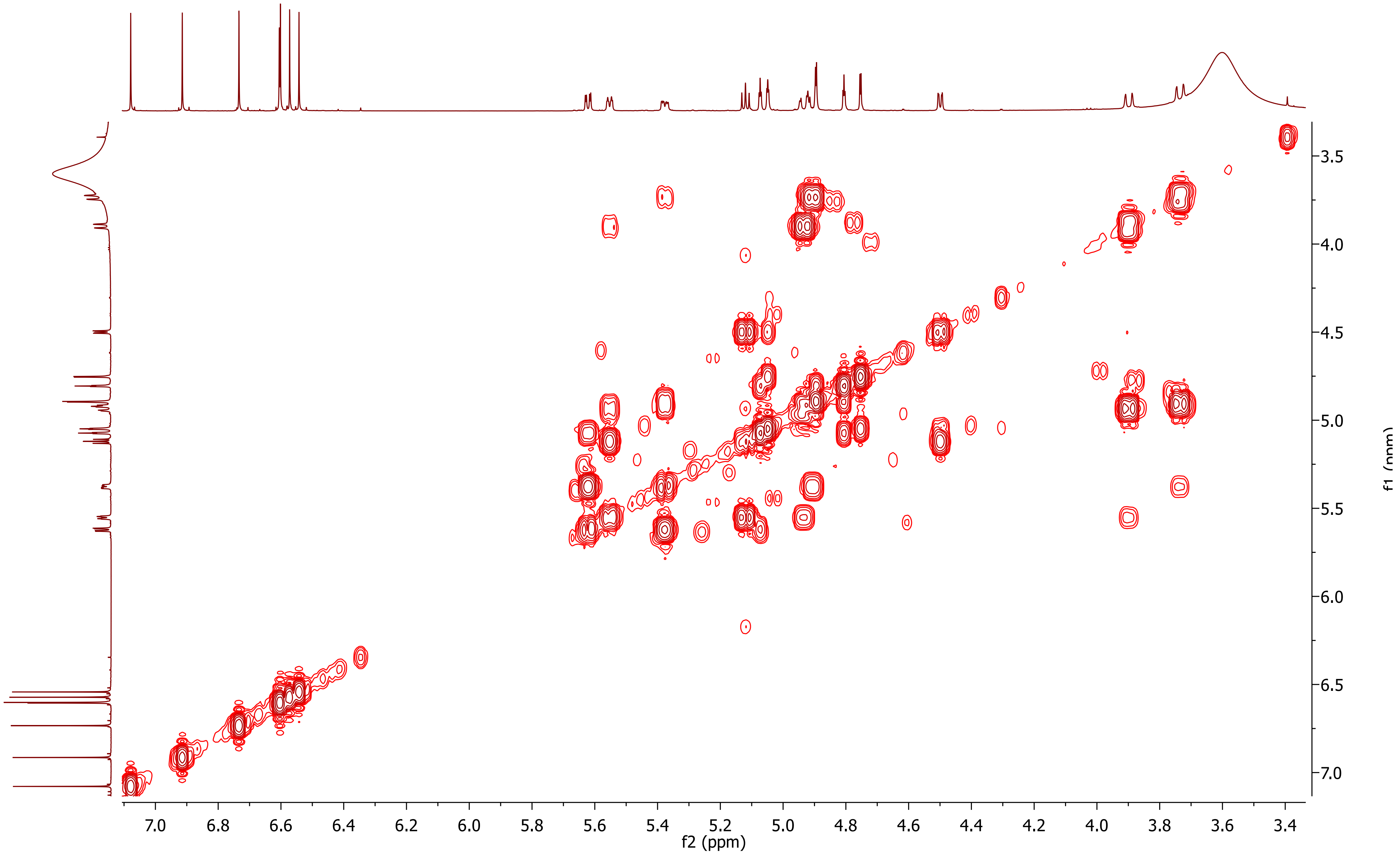

Figure S34. ^{1}H-^{1}H COSY spectrum of compound 7 [600 MHz, acetone-d_6 + D_2O (9+1)]

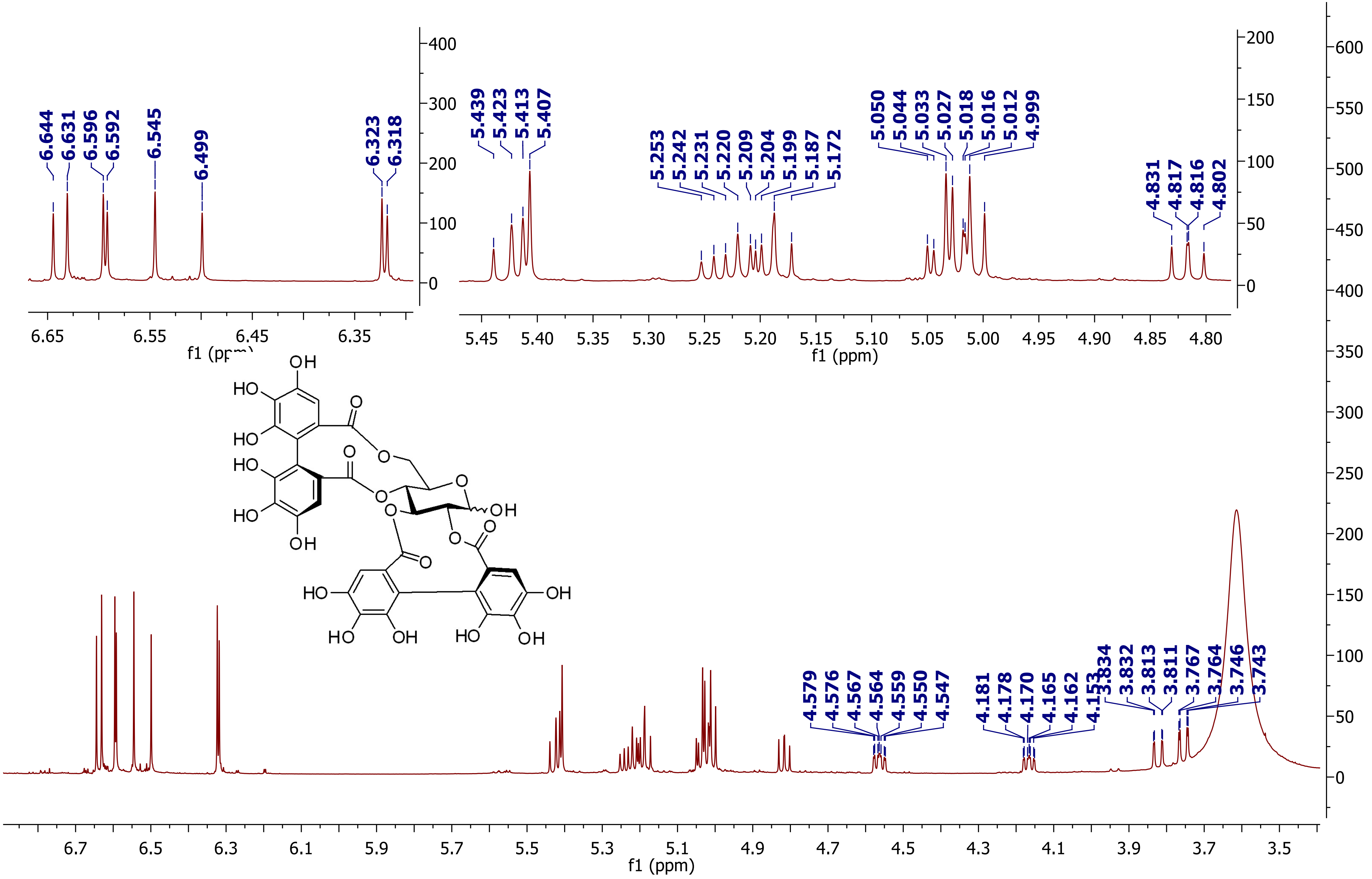

Figure S35. ^{1}H NMR spectrum of compound **8** [600 MHz, acetone-d_6 + D_2O (9+1)]

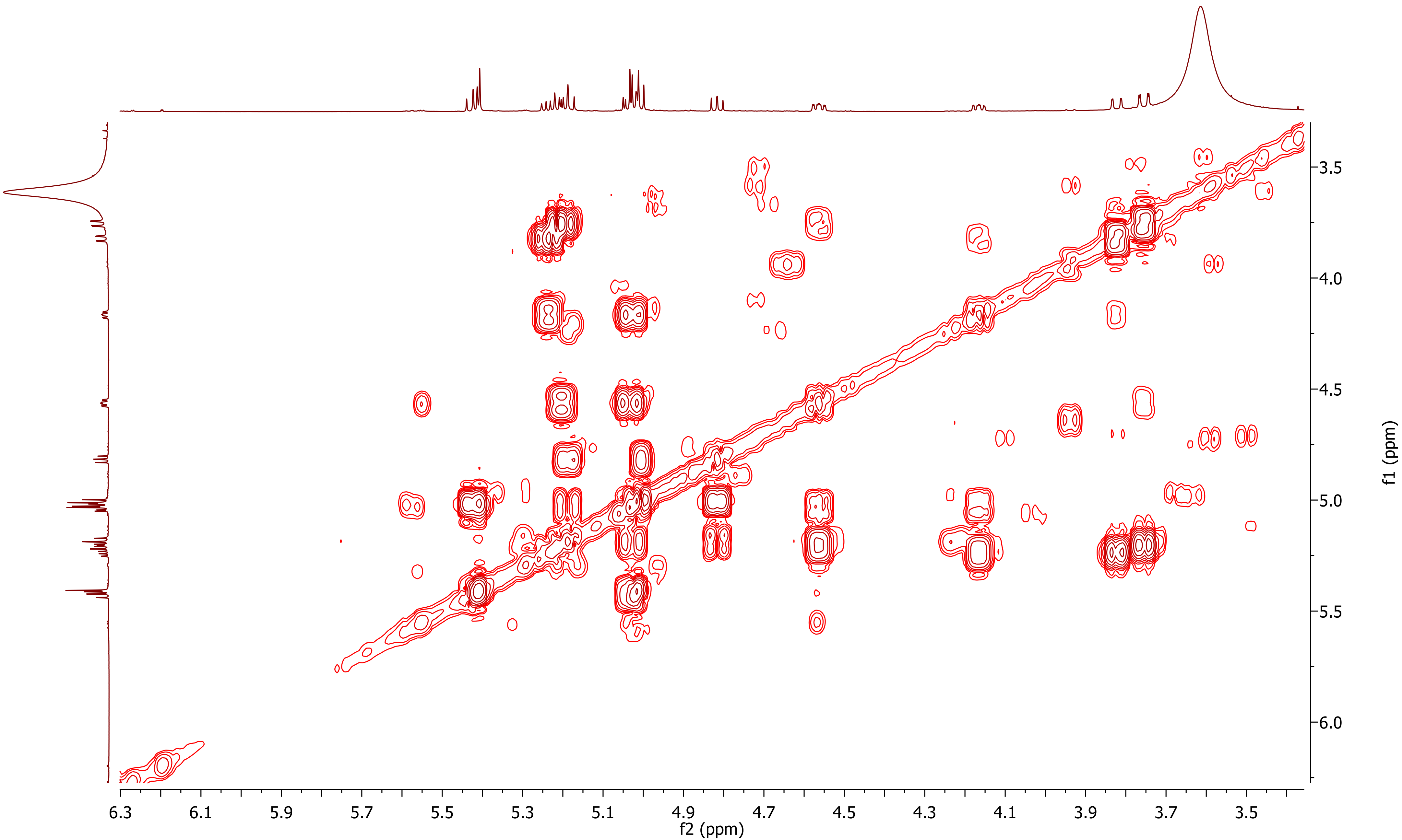

Figure S36. ^1H-^1H COSY spectrum of compound **8** [600 MHz, acetone-d_6 + D_2O (9+1)]

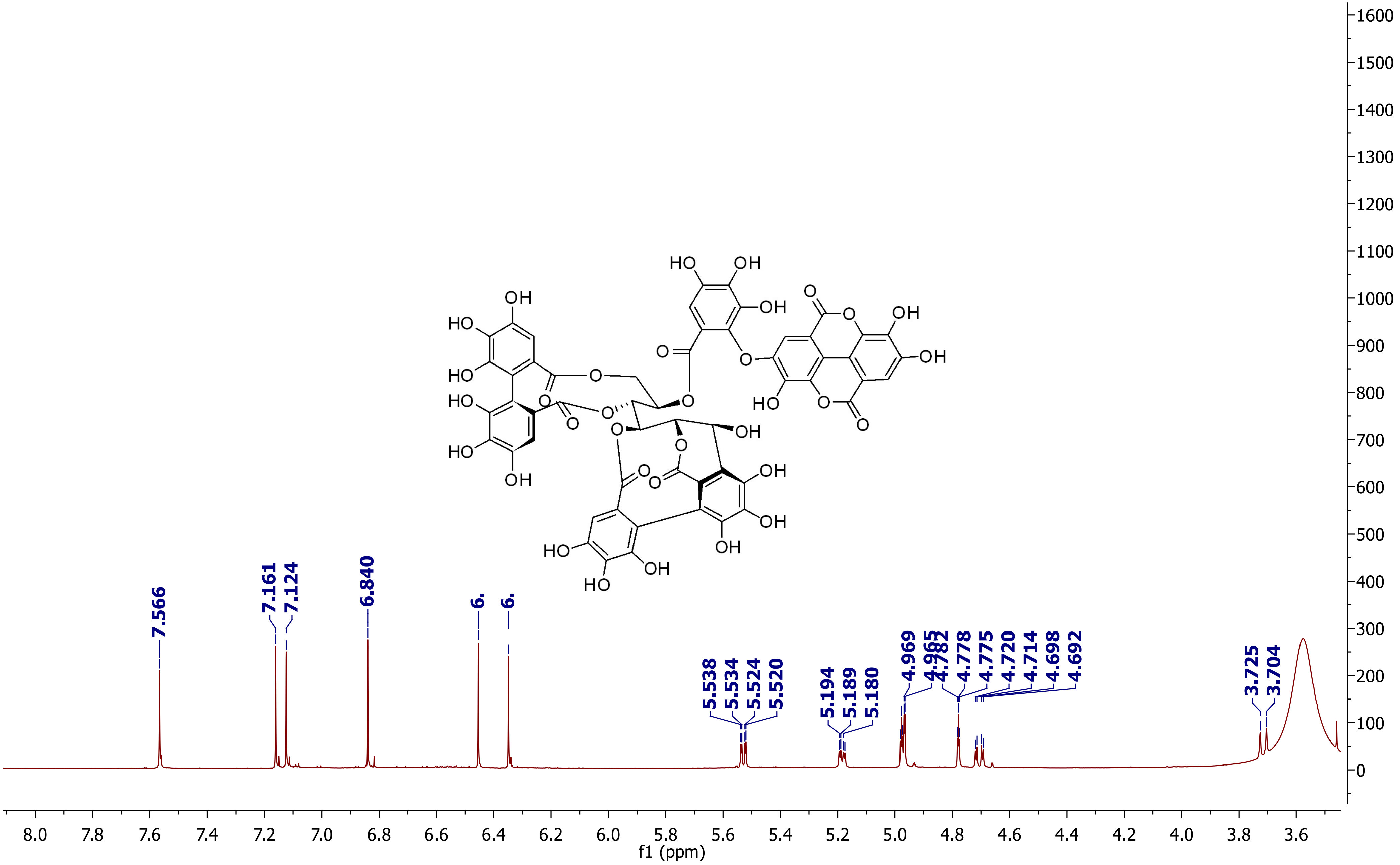

Figure S37. ^{1}H NMR spectrum of compound **9** [600 MHz, acetone-d_6 + D_2O (9+1)]

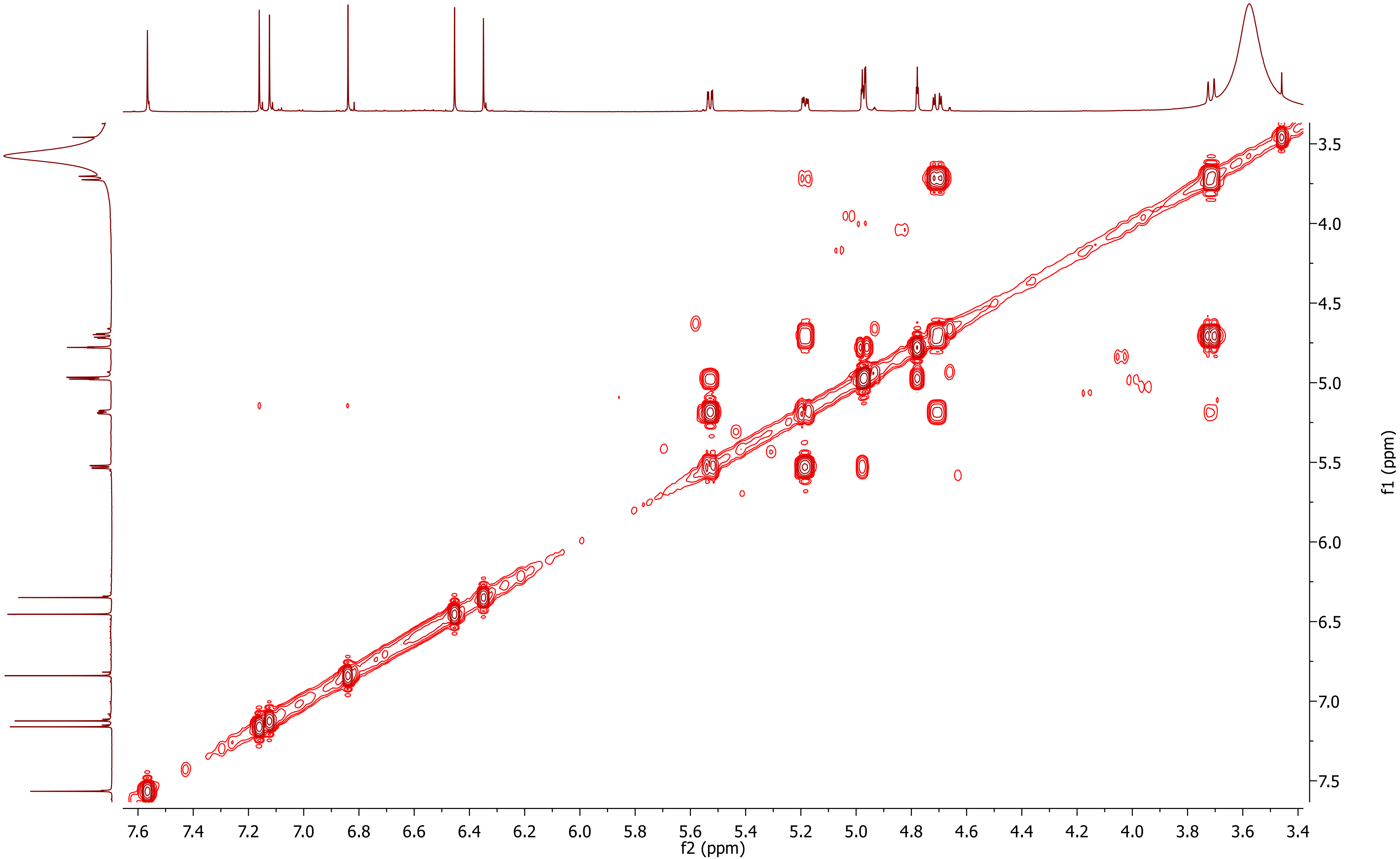

Figure S38. ^{1}H-^{1}H COSY spectrum of compound **9** [600 MHz, acetone-d_6 + D_2O (9+1)]

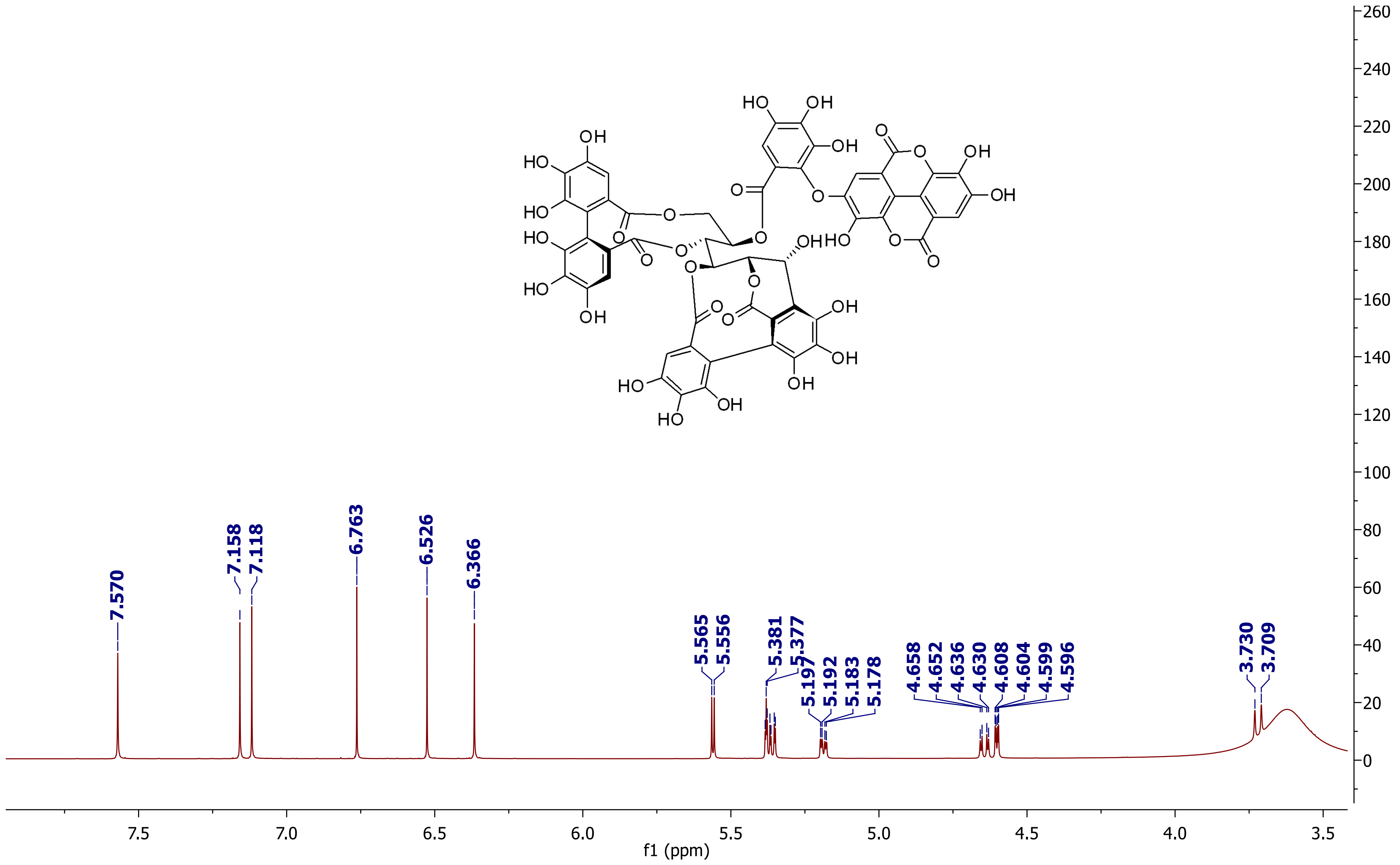

Figure S39. ^{1}H NMR spectrum of compound **10** [600 MHz, acetone-d_6 + D_2O (9+1)]

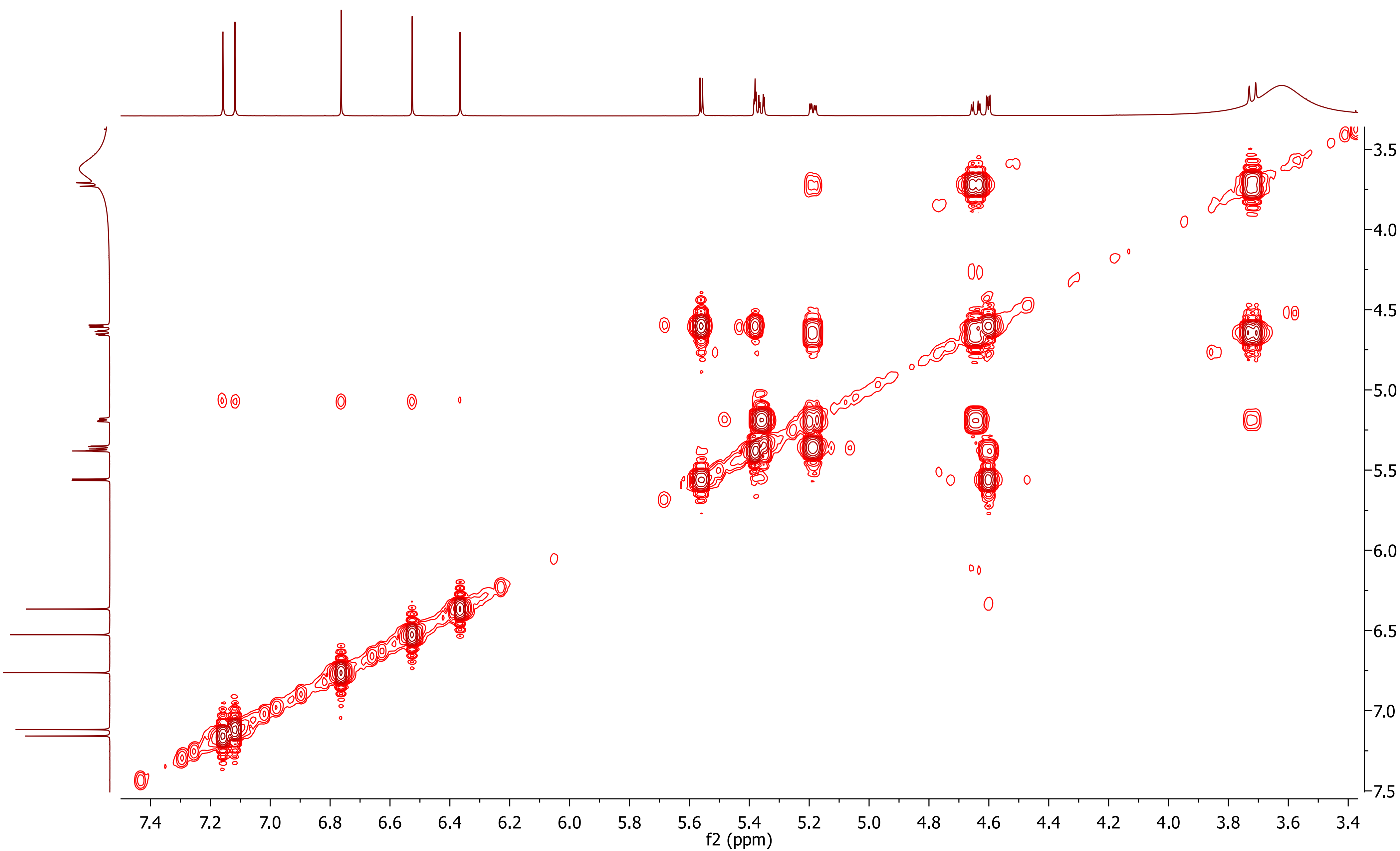

Figure S40. ^{1}H-^{1}H COSY spectrum of compound **10** [600 MHz, acetone-d_6 + D_2O (9+1)]

Experimental procedures of TD-DFT calculation of the ECD spectrum of 1

Quantum chemical calculations were performed with Gaussian16 [1]. The geometry of the various stereoisomers of compound **1** (1r3r, 1r3s, 1s3r, and 1s3s) were optimized in the gas phase with B3LYP/6-31+G and the resulting were confirmed to be stable structures (no imaginary frequencies). The resulting structures were then optimized with CAM-B3LYP/6-31G(d,p) in methanol using the polarizable continuum model (PCM) [2] and the optimized structures were used to calculate the ECD spectra with TD-DFT [3] at the same level. The CAM-B3LYP functional is chosen as it yields spectral properties in good agreement with experiment [4].

References

[1] Frisch, M. J.; Trucks, G. W.; Schlegel, H. B.; Scuseria, G. E.; Robb, M. A.; Cheeseman, J. R.; Scalmani, G.; Barone, V.; Petersson, G. A.; Nakatsuji, H.; Li, X.; Caricato, M.; Marenich, A. V.; Bloino, J.; Janesko, B. G.; Gomperts, R.; Mennucci, B.; Hratchian, H. P.; Ortiz, J. V.; Izmaylov, A. F.; Sonnenberg, J. L.; Williams-Young, D.; Ding, F.; Lipparini, F.; Egidi, F.; Goings, J.; Peng, B.; Petrone, A.; Henderson, T.; Ranasinghe, D.; Zakrzewski, V. G.; Gao, J.; Rega, N.; Zheng, G.; Liang, W.; Hada, M.; Ehara, M.; Toyota, K.; Fukuda, R.; Hasegawa, J.; Ishida, M.; Nakajima, T.; Honda, Y.; Kitao, O.; Nakai, H.; Vreven, T.; Throssell, K.; Montgomery, J. A., Jr.; Peralta, J. E.; Ogliaro, F.; Bearpark, M. J.; Heyd, J. J.; Brothers, E. N.; Kudin, K. N.; Staroverov, V. N.; Keith, T. A.; Kobayashi, R.; Normand, J.; Raghavachari, K.; Rendell, A. P.; Burant, J. C.; Iyengar, S. S.; Tomasi, J.; Cossi, M.; Millam, J. M.; Klene, M.; Adamo, C.; Cammi, R.; Ochterski, J. W.; Martin, R. L.; Morokuma, K.; Farkas, O.; Foresman, J. B.; Fox, D. J. Gaussian 16, revision A.03; Gaussian Inc.: Wallingford, CT, 2016.

[2] Tomasi, J., Mennucci, B. and Cammi, R., 2005. Quantum mechanical continuum solvation models. Chemical reviews, 105(8), pp.2999-3094.

[3] Runge, E. and Gross, E.K., 1984. Density-functional theory for time-dependent systems. Physical Review Letters, 52(12), p.997.

[4] El-Zohry, A.M., Orabi, E.A., Karlsson, M. and Zietz, B., 2021. Twisted Intramolecular Charge Transfer (TICT) Controlled by Dimerization: An Overlooked Piece of the TICT Puzzle. The Journal of Physical Chemistry A, 125(14), pp.2885-2894.

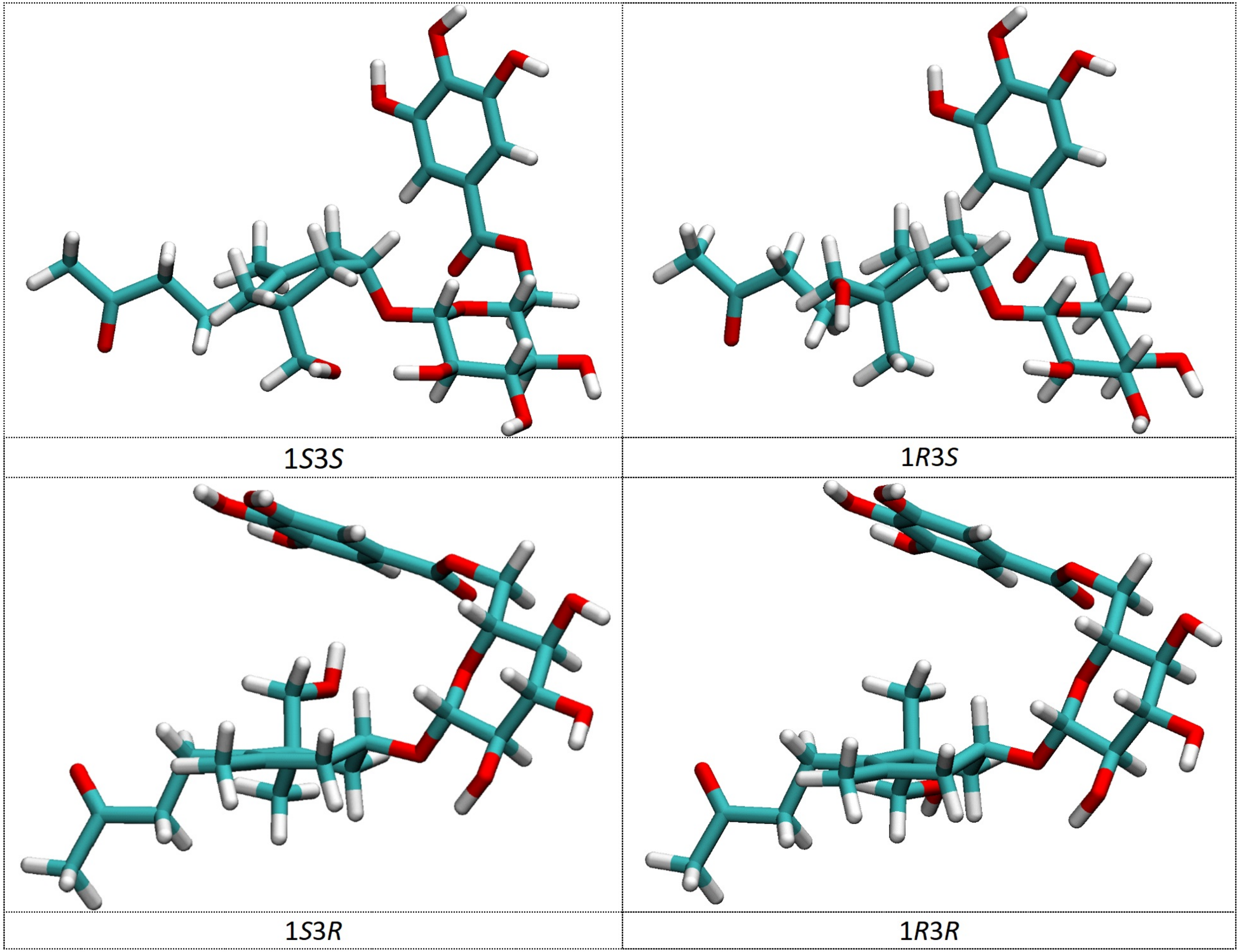

Figure S41. 3D structures of possible stereoisomers of compound **1**

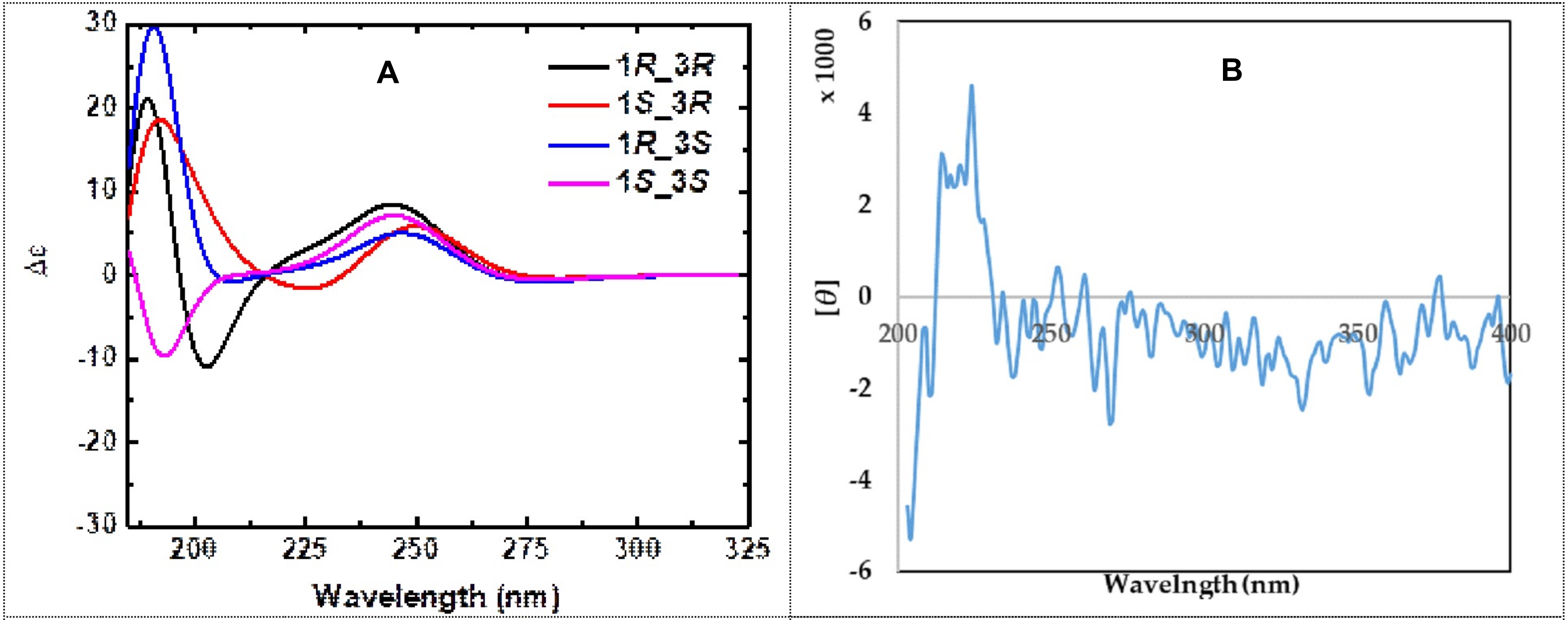

Calculated ECD spectrum of **1**	Practically measured ECD spectrum of **1**

Figure S42. Calculated (**A**) and practically measured (**B**) ECD spectra of compound **1**
